# Comparative efficacy of commercial oral poly-herbal traditional Chinese medicine formulations combined with western medicine in benign prostatic hyperplasia management: a systematic review and network meta-analysis

**DOI:** 10.3389/fphar.2024.1358340

**Published:** 2024-06-05

**Authors:** Hengda Zeng, Ziqiao Wang, Weian Zhu, Xiaoyang Li, Bohao Liu, Chuhao Chen, Peiying Huang, Yun Luo

**Affiliations:** ^1^ Department of Urology, The Third Affiliated Hospital, Sun Yat-sen University, Guangzhou, China; ^2^ The Second Clinical Medical College of Guangzhou University of Chinese Medicine, Guangzhou, China

**Keywords:** benign prostatic hyperplasia, commercial oral poly-herbal TCM formulations, Western medicine, efficacy, network meta-analysis

## Abstract

**Background:** Benign prostatic hyperplasia (BPH) is prevalent among the aging male population and often presents with distressing lower urinary tract symptoms. There is emerging evidence that commercial oral poly-herbal traditional Chinese medicine (TCM) formulation combined with Western medicine (WM) may offer enhanced therapeutic effects compared to WM alone in BPH treatment. Nevertheless, determining the optimal formulations for BPH remains controversial. We aimed to employ a network meta-analysis to compare and assess differences among commonly used and recommended poly-herbal TCM formulations outlined in the Chinese guidelines for BPH treatment, providing clinical medication recommendations and guidance.

**Methods:** We extensively searched for RCTs of BPH patients that had oral poly-herbal TCM formulations and WM treatment, covering both English and Chinese databases up to 31 October 2023. The quality of the included studies was evaluated using the Cochrane risk-of-bias tool Version 2 (ROB2). A Bayesian network meta-analysis was performed to assess the effectiveness of various formulations, followed by sensitivity and subgroup analyses.

**Results:** Our meta-analysis included 107 RCTs involving 11,037 patients across 16 oral poly-herbal TCM formulations. The quality of the selected studies was assessed as “Some concerns”. Most formulations combined with WM demonstrated superior therapeutic efficacy compared to WM alone. For clinical effective rate, Jingui Shenqi pill (JGSQ) + WM had the highest-ranking probability (87.38%). Concerning International Prostate Symptom Score (IPSS) and maximum flow rate of urine, Guizhi Fuling capsule (GZFL) + WM was most effective (91.10% and 98.55%). Regarding the quality of life score and postvoid residual urine, Pulean tablet (PLA) + WM ranked first (86.71% and 91.81%). In controlling prostate volume, Huange capsule (HE) + WM demonstrated the highest efficacy (95.65%). Additionally, among the interventions, Lingze (LZ) + WM capsule exhibited the lowest incidence of adverse drug reactions (2.32%).

**Conclusion:** Combining oral poly-herbal TCM formulations with WM may provide greater therapeutic benefits in BPH treatment compared to WM alone. JGSQ, GZFL, PLA, and HE emerged as promising treatment options. However, further rigorous empirical studies are essential to substantiate these findings.

**Systematic Review Registration:** https://www.crd.york.ac.uk/prospero/display_record.php?RecordID=459651, CRD 42023459651.

## 1 Introduction

Benign prostatic hyperplasia (BPH) is a common disease among older men, causing disturbing lower urinary tract symptoms (LUTS) (Chughtai et al., 2016). Approximately 50% of men aged over 50 years old experience pathological BPH, with this proportion escalating to over 80% in those aged 80 and above (Berry et al., 1984; Madersbacher et al., 2004). BPH is characterized by the excessive proliferation of epithelial and stromal cells in the periurethral region of the prostate (Roehrborn, 2008), frequently leading to noticeable changes in the size and structure of the prostate, resulting in LUTS including voiding symptoms (weak stream, splitting or spraying, intermittency, hesitancy, and straining), storage symptoms (frequency, urgency, nocturia), and post micturition symptoms (incomplete emptying and post micturition dribble) (Abrams et al., 2002; Martin et al., 2011). If left untreated, these symptoms can result in deterioration of bladder and kidney function, significantly impairing patient quality of life (Agarwal et al., 2014).

The management of patients with LUTS caused by BPH should be determined by the disease progression and symptom severity (Oelke et al., 2013; Kim et al., 2016). Mild cases may only require watchful waiting, while bothersome symptoms require lifestyle adjustments and specific treatments. Medication options include alpha 1-blockers for quick symptom relief (Djavan et al., 2004; Michel and Vrydag, 2006), 5 alpha-reductase inhibitors for enlarged prostates (Rittmaster et al., 1996; Naslund and Miner, 2007), antimuscarinics for bladder storage symptoms (Kaplan et al., 2008), phosphodiesterase type 5 inhibitors for both LUTS and erectile dysfunction (Gacci et al., 2012), and desmopressin for nocturia (Lose et al., 2004). In clinical practice, the combined administration of an α1-blocker and a 5-alpha-reductase inhibitor (5-ARI) or an antimuscarinic is a prevalent approach for BPH treatment (McConnell et al., 2003). Surgery, represented by transurethral resection of the prostate, is recommended for severe cases with absolute indications or in drug-resistant patients (Ahyai et al., 2010; Kim et al., 2016). However, surgery may also lead to complications and adverse reactions (Cornu et al., 2015; Miernik and Gratzke, 2020). Prostate stents are an option for patients unfit for surgery (Armitage et al., 2007), while ethanol or botulinum toxin injections remain experimental (Goya et al., 2004; Marberger et al., 2013).

Besides these treatment methods, Chinese clinicians often select commercial oral poly-herbal traditional Chinese medicine (TCM) formulations for the complementary treatment of prostate hyperplasia. Poly-herbal TCM formulations can alleviate LUTS and positively impact the prognosis of targeted populations (Zhang et al., 2016; 2023; Yu, 2022). However, there are numerous types of these formulations available for prostate hyperplasia and determining that which is most effective remains controversial.

Network meta-analysis, distinct from conventional pairwise meta-analysis, integrates direct and indirect comparisons among various interventions and provides ranking for efficacy comparisons between two or more interventions (Li et al., 2011; Mills et al., 2012; Salanti, 2012). We, therefore, aimed to employ a network meta-analysis to compare and assess differences among commonly used and recommended poly-herbal TCM formulations outlined in the Chinese guidelines for BPH treatment (Sun et al., 2017; Li, 2021; Yu, 2022), thus providing clinical medication recommendations and guidance.

## 2 Materials and methods

We conducted this network meta-analysis following the Preferred Reporting Items for Systematic Reviews and Meta-Analyses (PRISMA) Extension Statement. The checklist is detailed in Supplementary Material S1.

### 2.1 Search strategy

We performed a comprehensive search across nine academic databases to identify relevant published studies, including five English databases and four Chinese databases. The search included PubMed, Embase, Web of Science, Cochrane Library, Ovid-Medline, China National Knowledge Infrastructure (CNKI), Wanfang Database, Weipu Journal Database, and the Chinese Biomedical Literature Database. We also searched ClinicalTrials.gov for unpublished studies. The database search was conducted from inception until 31 October 2023 (Supplementary Material S2), and we included only published randomized controlled trials (RCTs) that met the defined inclusion criteria. We applied no restrictions to language, country, publishing entity, or trial phase.

### 2.2 Inclusion and exclusion criteria

We included patients diagnosed with BPH according to the diagnosis criteria, excluding those undergoing surgical treatment. Patients with comorbid urinary system diseases, such as prostatitis or urinary tract stones, as well as those with significant underlying medical conditions (e.g., advanced tumors, severe heart disease, or shock), were also excluded. The experimental group received a combination treatment of one type of poly-herbal TCM formulation and Western medicine (WM) without any additional traditional Chinese medicine interventions, including herbal injections, oral herbal decoctions, acupuncture, or massage. The selection of formulation aligned with the recommendations in Chinese guidelines or commonly used drugs in clinical practice (Sun et al., 2017; Li, 2021; Yu, 2022). The control group comprised patients who received WM treatment alone and those who received an additional type of poly-herbal TCM formulation in combination with WM. WM treatment primarily included alpha blockers, 5-alpha-reductase inhibitors, and other therapeutic regimens commonly used in clinical practice.

The primary outcomes of the included studies comprised one or more of the following measures. Clinical effectiveness was categorized as follows: Markedly Effective, most of the main symptoms and signs disappear, the International Prostate Symptom Score (IPSS) score decreases by more than 60%, or the urinary flow rate increases to 15 mL/s, or the residual urine volume decreases by more than 60%; Effective, the main symptoms and signs are partially alleviated or disappeared, the IPSS score decreases by 30%–59%, or the urinary flow rate increases by 30%, or the residual urine volume decreases by 30%–59%; Invalid, the main symptoms and signs remain unchanged or even worsen. The sum of the markedly effective and effective proportions is the clinical effective rate (National Administration of Traditional Chinese Medicine, 1994; Zheng, 2002).

We included the IPSS, scores measuring quality of life (QoL), maximum flow rate (Qmax), volume of the prostate, postvoid residual urine volume (PVR), and adverse drug reactions as secondary outcomes.

### 2.3 Data extraction

Based on the predefined inclusion and exclusion criteria, two independent reviewers thoroughly screened the original studies by carefully reviewing titles, abstracts, and full texts. The relevant information was extracted using a standardized form and subsequently entered into Excel software, including: 1) Trial information (title, authors, year of publication, and study site); 2) Study design (type of design, randomization method, allocation concealment, and blinding); 3) Population characteristics (sample size, participant demographics [age and gender], baseline comparisons, underlying diseases, diagnostic criteria, and course of illness); 4) Interventions such as treatment medications, dosage, administration, and duration; 5) Outcomes, including previously mentioned outcomes and the relevant data to calculate the overall effect size (number of participants/events, number of participants/mean/standard deviation); and 6) Additional information such as pharmaceutical company sponsorship.

### 2.4 Quality assessment

Two additional reviewers independently conducted quality assessments using Version 2 of the Cochrane risk-of-bias tool for randomized trials (RoB2) (Sterne et al., 2019). The assessment evaluated the randomization process, deviations from intended interventions, missing outcome data, measurement of the outcome, and selection of the reported results. The item “overall bias” summarizes the assessment derived from these components. Each component was categorized as “low risk,” “high risk,” or “some concerns.” An RCT was deemed “low risk” only when all components were assessed as such. The latest version of RoB2 (15 October 2020) was employed to generate a graph depicting the risk of bias. Furthermore, to ensure rigor and professionalism, we lowered the evidence level of the original researches to observational cohort studies and conducted evaluations using the Newcastle-Ottawa Scale (NOS) (Wells et al., 2000), assigning a maximum score of 9 to each study, with 4 points for cohort selection, ensuring that the exposure cohort is representative, the non-exposure cohort originates from the same population as the exposure cohort, exposure factors are rigorously determined, and the outcome of interest was not present at the start of the study. Additionally, 2 points are allocated for comparability, and 3 points are awarded for independent and reliable outcome measures and sufficiently long and complete follow-up. A score of <4 was considered to be a low-quality study, and a score of ≥7 was considered high quality. The reviewers attempted to reach a consensus regarding any disagreements during the data extraction or quality assessment process. If the disagreements persisted, a third reviewer provided final arbitration.

### 2.5 Data analysis

We performed both fix-effects and random-effects network meta-analysis within a Bayesian framework involving 200,000 iterations and 10,000 annealings to, directly and indirectly, compare all interventions (Salanti, 2012) and select the better model for the following analysis (Beliveau et al., 2019). Continuous variables are represented as mean differences with 95% confidence intervals (CIs), while categorical variables are expressed as risk ratios with 95% CI. Brooks–Gelman–Rubin plots were used to evaluate the goodness of fit of the model’s results (Brooks and Gelman, 1998). Nevertheless, due to the absence of closed loops in the intervention network, the application of a node-splitting method for local inconsistency evaluation was unnecessary. The league tables were created to summarize the comparisons among interventions for each relevant outcome. Based on the pooled effect size, each intervention was calculated as a probability for each rank, and the total ranking probability was calculated as the surface under the cumulative ranking curve (SUCRA) for clear visualization (Dias et al., 2013). Additionally, a cluster analysis was used to integrate two different outcomes and evaluate their efficacy between different interventions.

Furthermore, deviance information criterion (DIC), global I^2^-statistics, and predictive interval plots were utilized to measure the heterogeneity, with higher I^2^ values indicating more significant heterogeneity (Higgins and Thompson, 2002). To detect potential publication bias, a comparison-adjusted funnel plot was utilized for outcomes. A sensitivity analysis was conducted among studies published in the last decade, including only poly-herbal TCM formulations supported by more than three RCTs, and those adhering to mainstream and consistent dosage and usage according to clinical guidelines to ensure the robustness of the results. Additionally, a subgroup network meta-analysis was conducted, considering the potential influencing factors including age, treatment course, and sample size. The data analysis was performed using R 4.1.2 for network meta-analysis, assessment of global heterogeneity, probability rankings graph, sensitivity analysis, and subgroup analysis. STATA 14.0 was used for network plot, cluster analysis, predictive interval plot, and funnel plot.

## 3 Results

### 3.1 Search results and study characteristics

**FIGURE 1:**
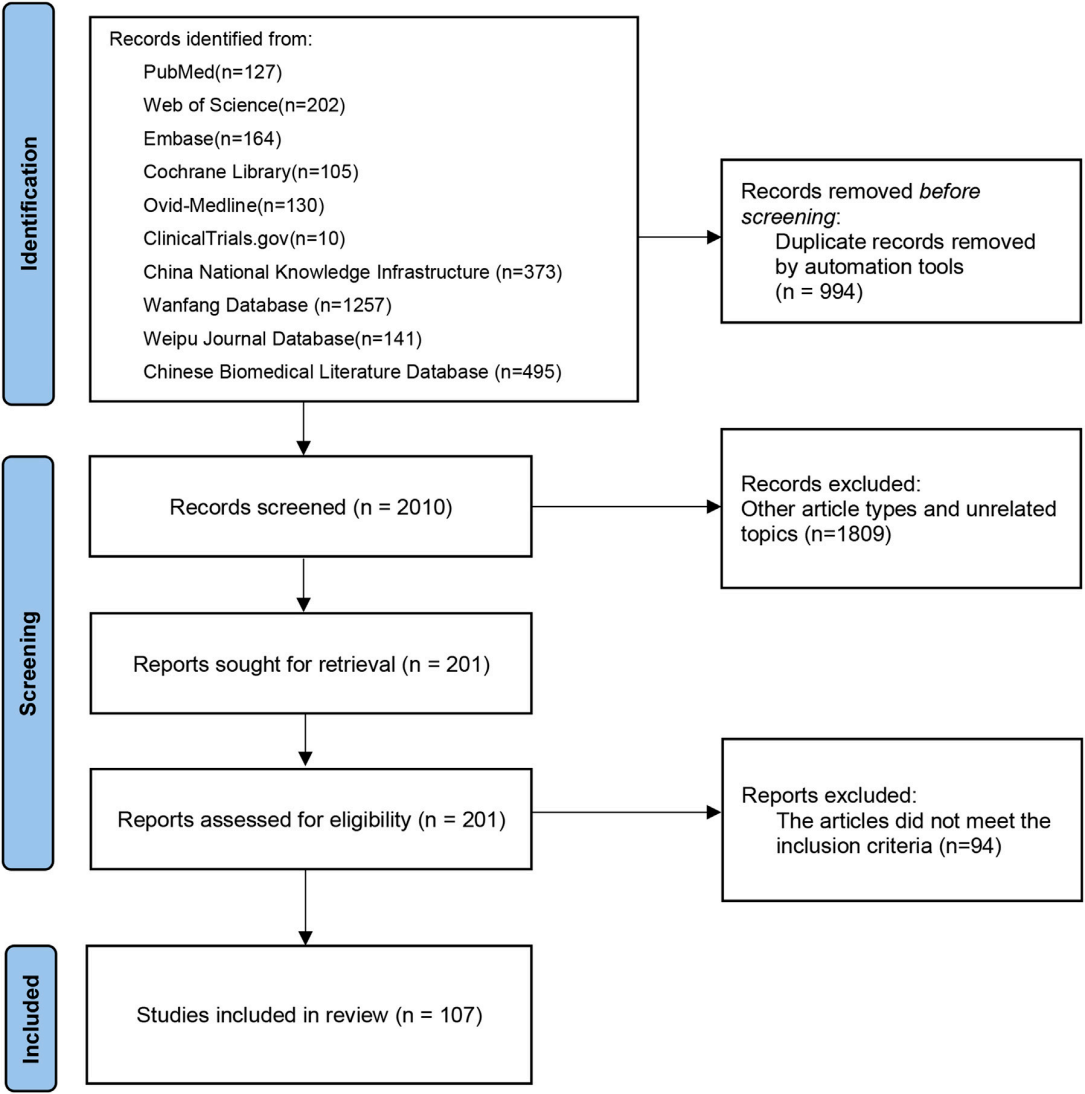
Flow chart for literature screening.

A total of 3,004 records were initially retrieved. Following removal of 994 duplicates, the remaining 2,010 records underwent screening based on predetermined criteria. Subsequently, 1,903 studies were excluded after thoroughly reviewing abstracts and full texts. Among these, some poly-herbal TCM formulations were excluded from our investigation due to a limited number of studies or suboptimal research quality. Ultimately, 107 published RCTs with 11,037 patients were included in the current analysis, comprising 106 two-arm trials and one three-arm trial (Supplementary Material S3). A flowchart depicting the literature screening process is presented in Figure 1.

We included 16 poly-herbal TCM formulations, comprising Guizhi Fuling Capsule (GZFL, three RCTs), Huange Capsule (HE, three RCTs), Jingui Shenqi Pill (JGSQ, eight RCTs), Longbishu Capsule (LBS, 17 RCTs), Liuwei Dihuang Pill (LWDH, three RCTs), Lingze Tablet (LZ, four RCTs), Pulean Tablet (Qianliekang Tablet) (PLA, five RCTs), Qianlie Beixi Capsule (QLBX, four RCTs), Qianlieshu Tablet (QLS, three RCTs), Qianlie Shutong Capsule (QLST, 33 RCTs), Qianlie Tongyu Capsule (QLTY, three RCTs), Qianliexin Capsule (QLX, five RCTs), Saw Palmetto Extract (SPE, seven RCTs), Wenglitong Capsule (WLT, three RCTs), Xialiqi Capsule (XLQ, three RCTs), and Zegui Longshuang Capsule (ZGLS, four RCTs). To ensure comparability and reproducibility, we made a detailed description on the compositions of these poly-herbal TCM formulations in accordance with the ConPhYMP guidelines (Heinrich et al., 2022). In addition, we collected information on adverse reactions, contraindications, and other relevant pharmacological data (Supplementary Material S4). From the 107 selected RCTs, 69, 95, 40, 89, 81, and 87 RCTs contributed data to the analysis of clinical effective rate, IPSS, QoL score, maximum flow rate of urine, prostate volume and postvoid residual urine, respectively. The baseline characteristics were balanced across various groups in all studies and the trial durations ranged from 4 to 52 weeks. More comprehensive insight into the selected studies is provided in Supplementary Material S5.

**FIGURE 2:**
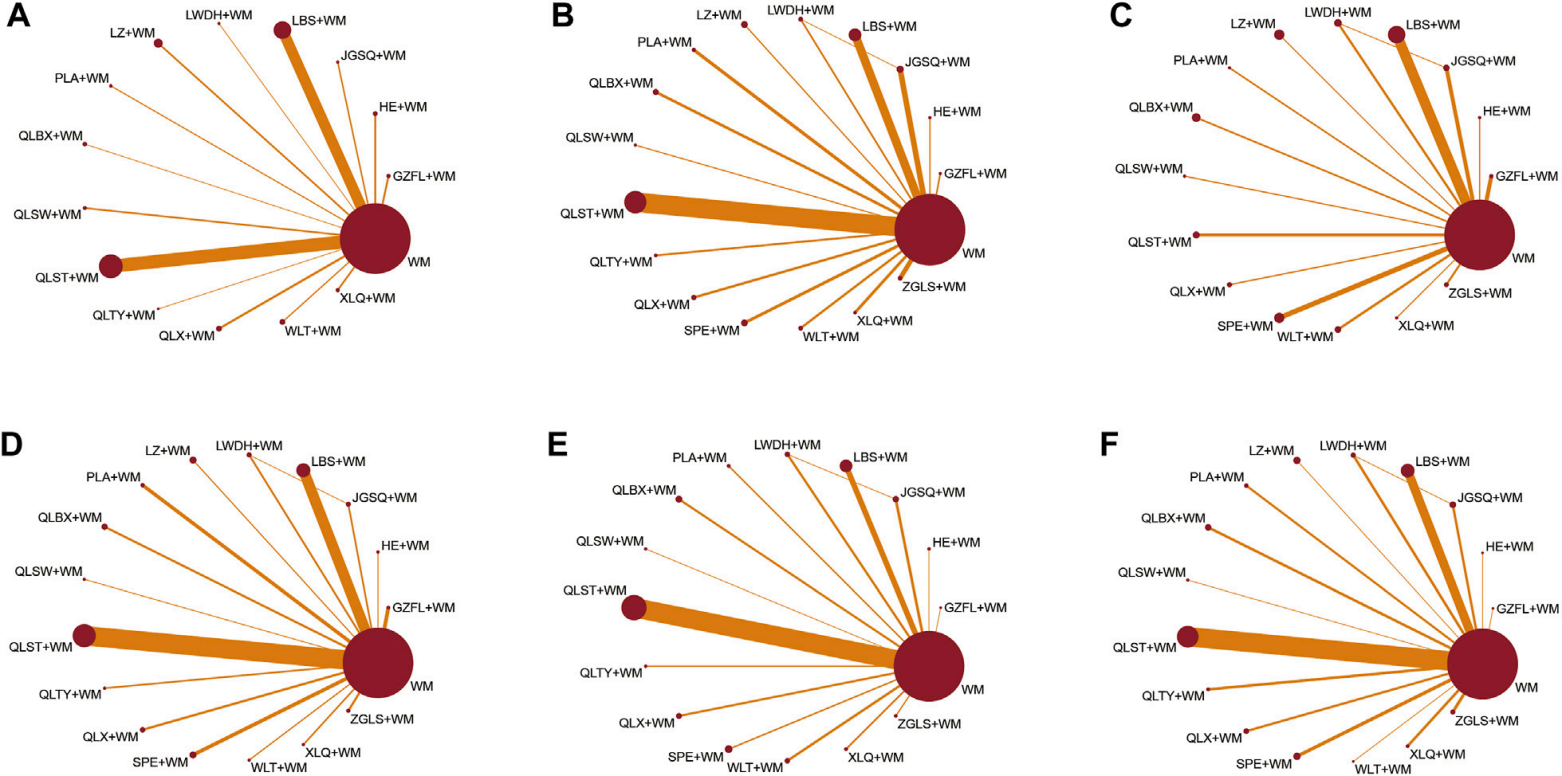
Network graphs of eligible comparisons for different outcomes. **(A)** Clinical effective rate; **(B)** IPSS; **(C)** QoL score; **(D)** Maximum flow rate of urine; **(E)** Prostate volume; **(F)** Postvoid residual urine; WM, Western medicine; GZFL, Guizhi Fuling capsule; HE, Huange capsule; JGSQ, Jingui Shenqi pill; LBS, Longbishu capsule; LWDH, Liuwei Dihuang pill; LZ, Lingze tablet; PLA, Pulean tablet; QLBX, Qianlie Beixi capsule; QLS, Qianlieshu pill; QLST, Qianlie Shutong capsule; QLTY, Qianlie Tongyu capsule; QLX, Qianliexin capsule; SPE, Saw Palmetto Extract capsule; WLT, Wenglitong capsule; XLQ, Xialiqi capsule; ZGLS, Zegui Longshuang Capsule.

Furthermore, a network graph was constructed to illustrate the relationships between different treatments. The size of each node in this graph represents the total number of participants receiving each treatment, while the width of the lines connecting the nodes indicates the standard error of each study involving those specific interventions (Figure 2).

### 3.2 Risk-of-bias assessment

In the selected RCTs, 41 trials specified their randomization methods, including 32 trials applying random number tables, four trials using random lottery method, four trials implementing computer randomization, and one using stratified randomization. Only three studies reported allocation concealment and were consequently rated as “low risk” in the “randomization process” domain (2.8%). Blinding was reported in six studies, comprising three single-blind trials and three double-blind trials. However, only three trials were considered “low risk” in “deviation from intended interventions” (2.8%), as they appropriately analyzed the assignment of interventions. Most trials with randomized patients had outcome data, while few patients lost to follow-up were not deemed to be related to the value of outcomes. Consequently, there were few effects on outcomes assessment and all the trials were evaluated as “low risk” in the domain of “missing outcomes data” (100%). Furthermore, all selected studies were evaluated as “low risk” in the “measurement of outcome” for their use of appropriate methods for outcomes measurement and consistent application throughout the trials, thereby ensuring objectivity. Nearly all articles reported outcomes without evidence of selective result reporting, with ratings of “some concerns” in the “selection of the reported result.” Overall, the trials included in this network meta-analysis were rated as “some concerns.”

We used the NOS to score the studies included in the meta-analysis, and found a total of 90 studies with a score of 9, 12 with a score of 8, three with a score of 7, and two with a score of 6. The quality of the literature was generally high, with 105 high-quality studies and no low-quality studies. Therefore, we concluded that the quality of the included studies was sufficient for the meta-analysis, as summarized in Supplementary Material S6.

### 3.3 Network meta-analysis

In the Brooks–Gelman–Rubin diagnostic plots, convergence is indicated as all median and 97.5% interval lines approximate toward unity (1.0), signifying robust model fit across the analyses conducted in each outcome. These plots can be found for reference in Supplementary Material S7.

### 3.4 Primary outcome

#### 3.4.1 Clinical effective rate

**FIGURE 3:**
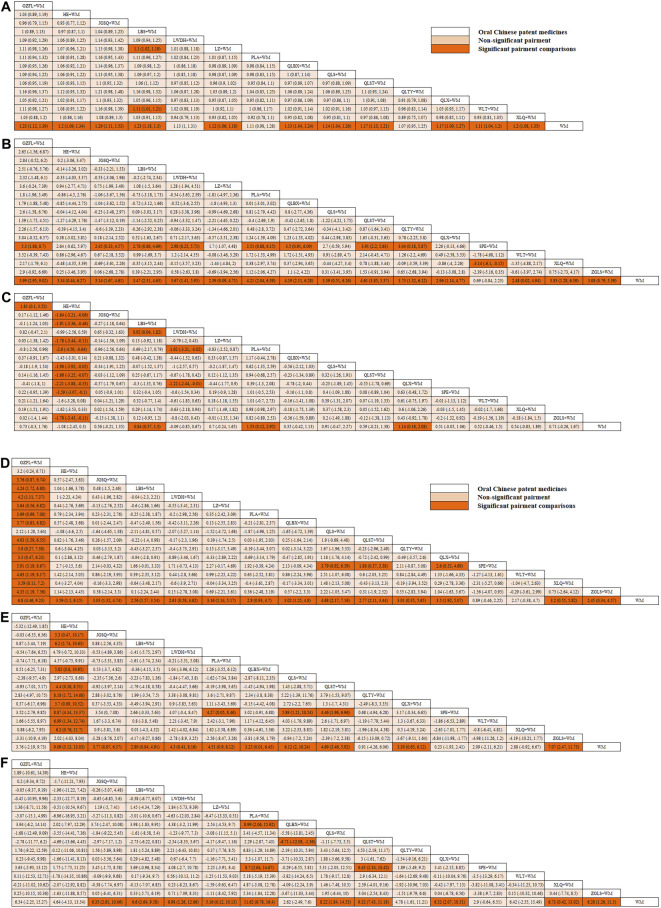
(Contiuned). League tables of comparison between each intervention for each outcome. **(A)** Clinical effective rate; **(B)** International Prostate Symptom Score; **(C)** QoL score; **(D)** Maximum flow rate of urine; **(E)** Prostate volume; **(F)** Postvoid residual urine; WM, Western medicine; GZFL, Guizhi Fuling capsule; HE, Huange capsule; JGSQ, Jingui Shenqi pill; LBS, Longbishu capsule; LWDH, Liuwei Dihuang pill; LZ, Lingze tablet; PLA, Pulean tablet; QLBX, Qianlie Beixi capsule; QLS, Qianlieshu pill; QLST, Qianlie Shutong capsule; QLTY, Qianlie Tongyu capsule; QLX, Qianliexin capsule; SPE, Saw Palmetto Extract capsule; WLT, Wenglitong capsule; XLQ, Xialiqi capsule; ZGLS, Zegui Longshuang Capsule.

**FIGURE 4:**
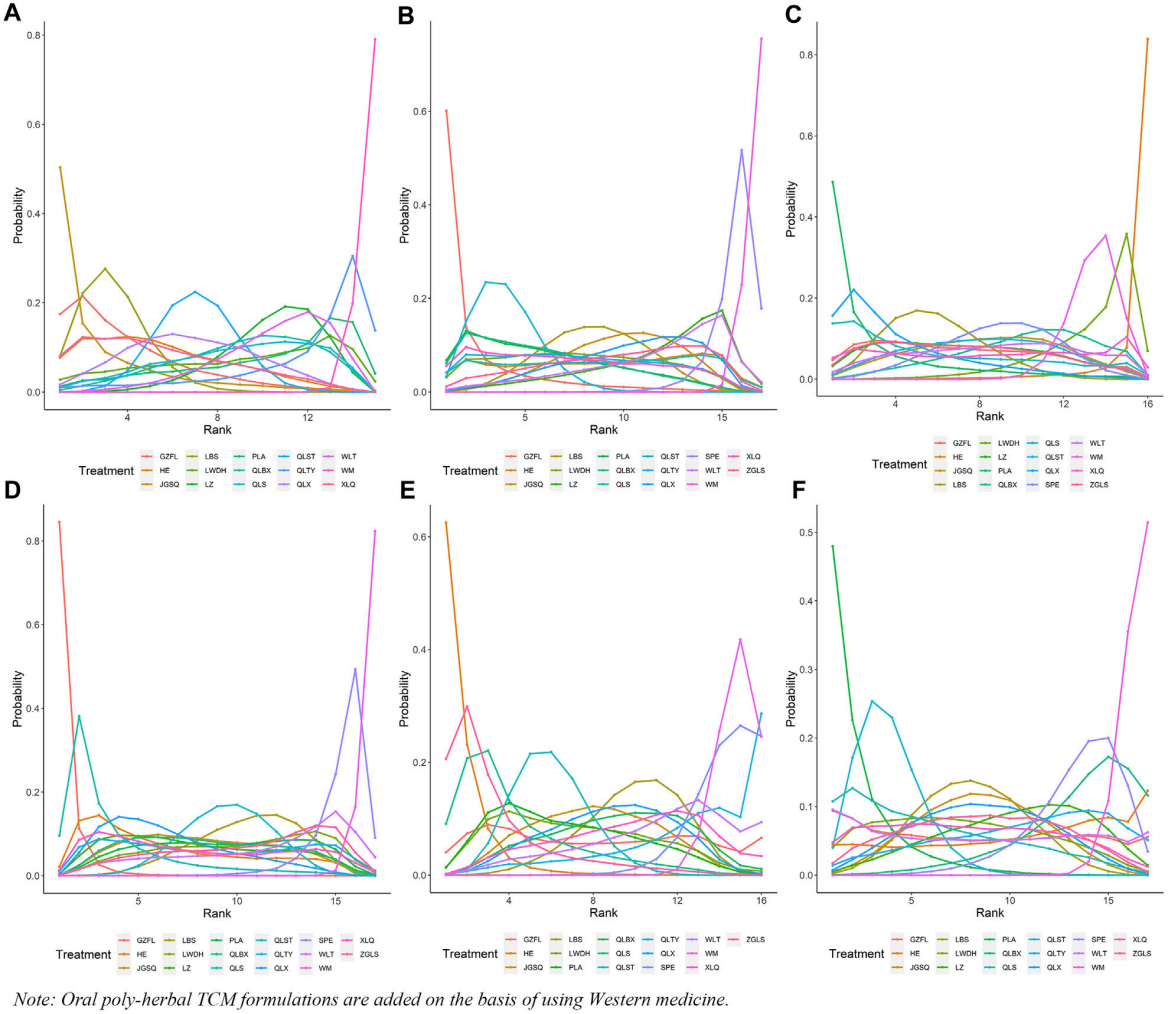
Ranking probability of each outcome for the included interventions. **(A)** Clinical effective rate; **(B)** International Prostate Symptom Score; **(C)** QoL score; **(D)** Maximum flow rate of urine; **(E)** Prostate volume; **(F)** Postvoid residual urine; WM, Western medicine; GZFL, Guizhi Fuling capsule; HE, Huange capsule; JGSQ, Jingui Shenqi pill; LBS, Longbishu capsule; LWDH, Liuwei Dihuang pill; LZ, Lingze tablet; PLA, Pulean tablet; QLBX, Qianlie Beixi capsule; QLS, Qianlieshu pill; QLST, Qianlie Shutong capsule; QLTY, Qianlie Tongyu capsule; QLX, Qianliexin capsule; SPE, Saw Palmetto Extract capsule; WLT, Wenglitong capsule; XLQ, Xialiqi capsule; ZGLS, Zegui Longshuang Capsule.

In this investigation, 15 treatment modalities were evaluated for the clinical effective rate, including GZFL + WM, HE + WM, JGSQ + WM, LBS + WM, LWDH + WM, LZ + WM, PLA + WM, QLBX + WM, QLS + WM, QLST + WM, QLTY + WM, QLX + WM, WLT + WM, XLQ + WM, and WM alone. Analysis of relative risk and 95% CIs of all pairwise interventions indicated that most poly-herbal TCM formulation + WM combinations were more effective than WM alone, excluding LWDH + WM, PLA + WM, and QLTY + WM. Notably, LBS + WM demonstrated superior efficacy over LZ + WM and WLT + WM. However, such significant effects were not observed in other pairwise comparisons. The comparison between each intervention is revealed in Figure 3A. Additionally, based on the ranking probability plot and the SUCRA, JGSQ + WM had the highest effective rate (87.38%), closely followed by LBS + WM (82.98%) and GZFL + WM (79.28%), while WM alone demonstrated the most minor effectiveness (1.57%). The ranking probability is presented in Figure 4A and Table 1.

**TABLE 1:** Ranking probabilities of surface under the cumulative ranking area curves (SUCRA) for the outcomes.

	Clinical effect rate (%)	IPSS (%)	Quality of life (%)	Maximum flow rate of urine (%)	Prostate volume (%)	Postvoid residual urine (%)
GZFL + WM	79.28	91.10	59.30	98.55	51.70	54.08
HE + WM	68.13	50.91	2.91	64.85	95.65	40.58
JGSQ + WM	87.38	45.04	50.87	53.97	52.83	53.07
LBS + WM	82.98	53.06	67.69	40.35	40.35	55.73
LWDH + WM	42.67	57.68	17.63	43.30	58.99	58.68
LZ + WM	32.39	31.90	58.14	56.55	-	42.25
PLA + WM	34.61	68.86	86.71	48.13	61.72	91.81
QLBX + WM	41.61	68.85	38.81	53.62	45.96	21.97
QLS + WM	43.08	51.79	65.94	82.82	77.78	68.29
QLST + WM	56.60	81.10	52.12	46.72	66.95	82.21
QLTY + WM	21.96	58.79	-	52.48	22.65	40.05
QLX + WM	58.13	41.58	78.79	65.55	47.73	52.80
SPE + WM	-	8.14	47.36	10.31	11.31	22.40
WLT + WM	31.96	33.73	48.37	35.01	32.71	53.21
XLQ + WM	67.65	60.58	49.60	56.49	41.63	56.31
ZGLS + WM	-	45.23	58.51	40.12	84.15	52.57
WM	1.57	1.63	17.25	1.19	7.89	4.01

WM, western medicine; GZFL, guizhi fuling capsule; HE, huange capsule; JGSQ, jingui shenqi pill; LBS, longbishu capsule; LWDH, liuwei dihuang pill; LZ, lingze tablet; PLA, pulean tablet; QLBX, qianlie beixi capsule; QLS, qianlieshu pill; QLST, qianlie shutong capsule; QLTY, qianlie tongyu capsule; QLX, qianliexin capsule; SPE, saw palmetto extract capsule; WLT, wenglitong capsule; XLQ, xialiqi capsule; ZGLS, zegui longshuang capsule.

### 3.5 Secondary outcomes

#### 3.5.1 IPSS

All 17 interventions were analyzed for their impact on the IPSS, involving GZFL + WM, HE + WM, JGSQ + WM, LBS + WM, LWDH + WM, LZ + WM, PLA + WM, QLBX + WM, QLS + WM, QLST + WM, QLTY + WM, QLX + WM, SPE + WM, WLT + WM, XLQ + WM, ZGLS + WM, and WM alone. Excluding SPE + WM, each poly-herbal TCM formulation + WM pairing demonstrated a pronounced reduction in IPSS compared to WM monotherapy. Meanwhile, GZFL + WM, JGSQ + WM, LBS + WM, LWDH + WM, PLA + WM, QLBX + WM, QLST + WM, QLTY + WM, and XLQ + WM showed greater efficacy in decreasing IPSS than SPE + WM. Figure 3B provides a detailed comparative analysis of each intervention. Based on the ranking probability of each level and SUCRA, GZFL + WM ranked first in reducing IPSS (91.10%), followed by QLST + WM (81.10%) and PLA + WM (68.86%), whereas WM alone obtained the worst effect (1.63%). The ranking probability is presented in Figure 4B and Table 1.

#### 3.5.2 QoL score

There were 16 interventions involved in the analysis of QoL score, including GZFL + WM, HE + WM, JGSQ + WM, LBS + WM, LWDH + WM, LZ + WM, PLA + WM, QLBX + WM, QLS + WM, QLST + WM, QLX + WM, SPE + WM, WLT + WM, XLQ + WM, ZGLS + WM, and WM alone. Our analysis showed that LBS + WM, PLA + WM, and QLX + WM had significant implications for improving QoL. Except for LWDH + WM, QLBX + WM, WLT + WM, XLQ + WM, and WM alone, the other interventions showed a better effect than HE + WM on improving QoL. More details about the between-intervention differences are presented in Figure 3C. Ranking probability plots and SUCRA results showed that PLA + WM were the most effective in improving QoL, followed by QLX + WM (78.79%) and LBS + WM (67.69%). The ranking probability is shown in Figure 4A and Table 1.

#### 3.5.3 Maximum flow rate of urine

All 17 interventions were evaluated for maximum flow rate of urine. All poly-herbal TCM formulations except WLT + WM and SPE + WM revealed a higher effect in increasing the maximum urinary flow rate than WM alone. GZFL + WM showed significantly better efficacy than other drugs, excluding HE + WM and QLS + WM. This is consistent with the results presented by ranking probability and SUCRA, where GZFL + WM (98.55%) ranks first among all intervention measures, followed by QLS + WM (82.82%) and QLX + WM (65.55%). The detailed comparative analysis is presented in Figure 3D and the ranking probability is demonstrated in Figure 4D and Table 1.

#### 3.5.4 Prostate volume

Sixteen treatment nodes were compared in prostate volume change, including GZFL + WM, HE + WM, JGSQ + WM, LBS + WM, LWDH + WM, PLA + WM, QLBX + WM, QLS + WM, QLST + WM, QLTY + WM, QLX + WM, SPE + WM, WLT + WM, XLQ + WM, ZGLS + WM, and WM alone. Apart from GZFL + WM, QLTY + WM, SPE + WM, WLT + WM, and XLQ + WM, all other intervention measures have shown better improvement than WM alone. Notably, in terms of prostate volume, HE + WM demonstrated superior efficacy over most other interventions. The detailed comparison between each intervention is revealed in Figure 3E. Based on the ranking probability of each level and SUCRA, HE + WM had the highest SUCRA value (95.65%), followed by ZGLS + WM (84.15%) and QLS + WM (77.78%). The ranking probability is shown in Figure 4E and Table 1.

#### 3.5.5 Postvoid residual urine

All 17 treatments involved postvoid residual urine analyses. Among these, JGSQ + WM, LBS + WM, LWDH + WM, LZ + WM, PLA + WM, QLS + WM, QLST + WM, QLX + WM, and XLQ + WM revealed a higher effect in the decrease of postvoid residual urine. The comparison between each intervention is presented in Figure 3F. Ranking probability plots and SUCRA results showed that PLA + WM had the highest effect (91.81%), followed by QLST + WM (82.21%) and QLS + WM (68.29%). The ranking probability is demonstrated in Figure 4F and Table 1.

### 3.6 Adverse reactions

Only a few included studies reported results related to adverse events. Among the 3,758 patients observed, 278 cases of adverse reactions were reported. The most common reactions included 70 cases of gastrointestinal reactions, 56 cases of dizziness, 47 cases of nausea and vomiting, 33 of sexual dysfunction, 29 headaches, and 25 instances of hypotension. These adverse reactions were generally mild, with most resolving spontaneously without intervention, and no severe adverse reactions occurred. Among the 873 patients receiving LBS + WM, the most common adverse reactions were 27 cases of gastrointestinal reactions (3.09%) and 21 cases of nausea and vomiting (2.41%), with a total adverse reaction incidence of 6.87%. Among the 899 patients treated with QLST + WM, the most common adverse reactions were dizziness (2.56%), nausea and vomiting (1.67%), gastrointestinal reactions (1.22%), and hypotension (0.78%), with a total adverse reaction incidence of 7.9%. Further details on additional adverse reactions can be found in Supplementary Material S8.

### 3.7 Cluster analysis

**FIGURE 5:**
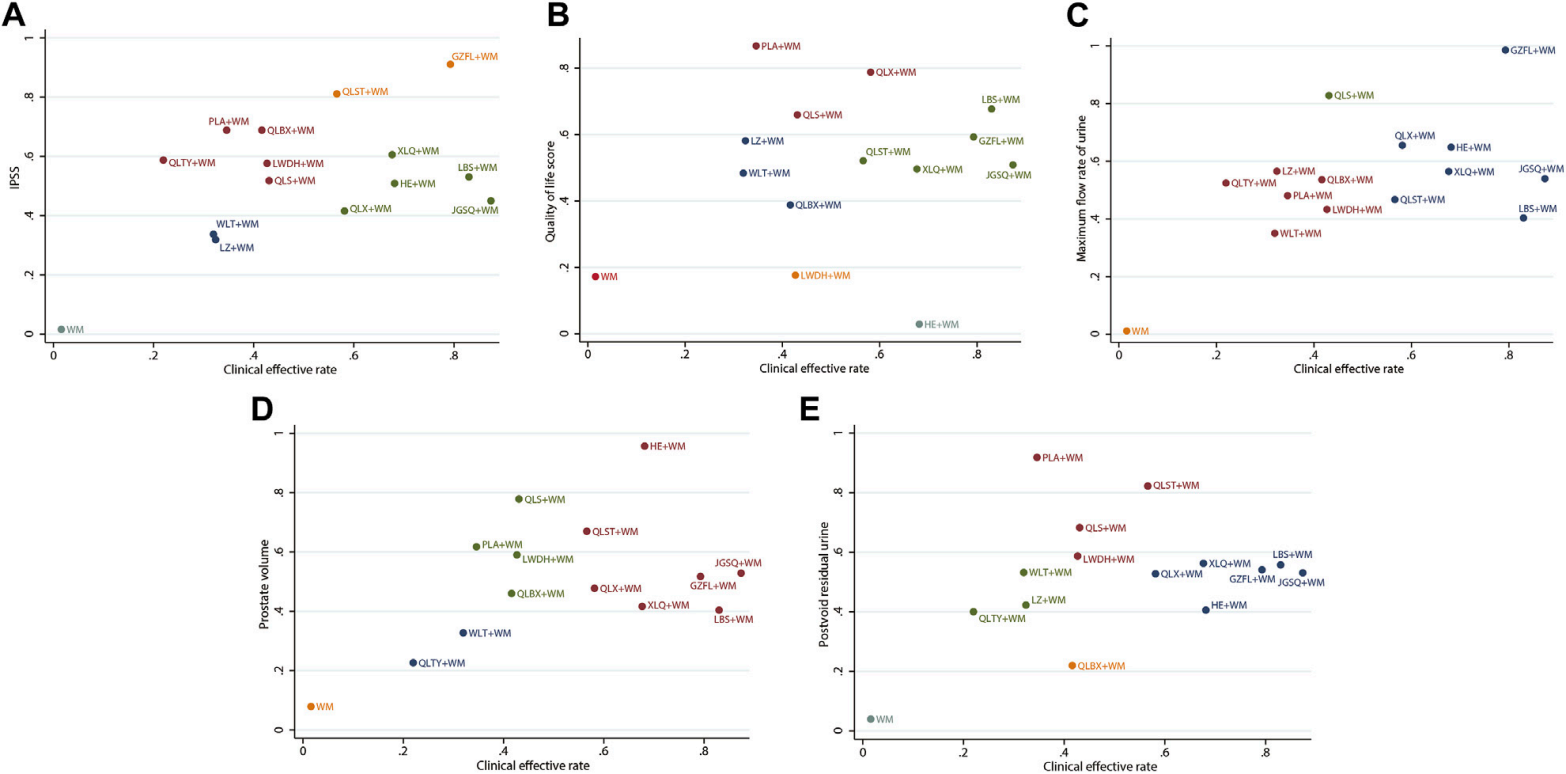
Cluster analysis plots **(A)** Clinical effective rate (x-axis) and IPSS (y-axis) **(B)** Clinical effective rate (x-axis) and QoL score (y-axis) **(C)** Clinical effective rate (x-axis) and maximum flow rate of urine (y-axis) **(D)** Clinical effective rate (x-axis) and prostate volume (y-axis) **(E)** Clinical effective rate (x-axis) and postvoid residual urine (y-axis); WM, Western medicine; GZFL, Guizhi Fuling capsule; HE, Huange capsule; JGSQ, Jingui Shenqi pill; LBS, Longbishu capsule; LWDH, Liuwei Dihuang pill; LZ, Lingze tablet; PLA, Pulean tablet; QLBX, Qianlie Beixi capsule; QLS, Qianlieshu pill; QLST, Qianlie Shutong capsule; QLTY, Qianlie Tongyu capsule; QLX, Qianliexin capsule; WLT, Wenglitong capsule; XLQ, Xialiqi capsule. Interventions grouped by identical colors are categorized within the same cluster. Therapeutic options situated in the upper-right corner of the analysis represent optimal treatments across two distinct outcomes, while the lower-left corner represent the least effective therapies.

To integrate and correlate the clinical efficacy rate with other corresponding outcomes, we conducted a cluster analysis, underpinned by the SUCRA of various interventions. As depicted in Figure 5, GZFL + WM, LBS + WM, QLX + WM, HE + WM, and QLST + WM consistently manifested superior therapeutic effects compared to the other treatment options. Meanwhile, the interventions grouped by the same color exhibited similar efficacy.

### 3.8 Heterogeneity and publication bias

Analytical evaluations were conducted on the data utilizing both fixed- and random-effect models. The analysis results and the assessment of model fit revealed that, for identical outcomes, the random-effect model demonstrated a more favorable profile, characterized by the lower DIC value and diminished heterogeneity. Global I^2^-statistics were revealed as 0, 1%, 0.7%, 0.5%, 0, and 0, corresponding to clinical effective rate, IPSS, QoL score, the maximum flow rate of urine, prostate volume, and postvoid residual urine, respectively. Detailed information is provided in Supplementary Material S9.

The predictive interval plots suggested that 0, 15.44%, 12.5%, 21.32%, 18.33%, and 8.82% of the comparisons of effective clinical rate, IPSS, QoL score, maximum flow rate of urine, prostate volume, and postvoid residual urine, respectively, were affected by the estimated heterogeneity. The predictive interval plots are presented in Supplementary Material S10.

The funnel plot evaluating the publication bias indicated that, while a small subset of points displayed bias, the predominant trend is relative symmetry around the central line. This distribution of data points indicated the relative reliability and high quality of the articles considered in the analysis. The funnel plots are detailed in Supplementary Material S11.

### 3.9 Sensitivity analyses and subgroup analyses

Sensitivity analyses were conducted on the studies published within the last decade, the studies of poly-herbal TCM formulations with more than three RCTs for each outcome, and those with consistent dosage and usage. These analyses robustly affirmed the superior therapeutic benefits of combining poly-herbal TCM formulations with WM across diverse outcomes compared to WM alone. Notably, the trend of enhancing efficacy was consistent, with only a limited number of poly-herbal TCM formulations being excluded. Comprehensive results of these analyses are detailed in Supplementary Material S12.

Subsequent subgroup analyses provided insightful distinctions, revealing that different poly-herbal TCM formulations combined with WM exhibited enhanced effectiveness over WM alone across different age demographics and treatment durations. LZ + WM and PLA + WM demonstrated a higher clinical effective rate in patient cohorts aged 65 years or below, whereas HE + WM and QLS + WM showed greater efficacy in those above 65 years. The control of prostate enlargement was more evident in patients over the age of 65 across all poly-herbal TCM formulations combined with WM. Additionally, with extended treatment duration (≥3 months), most poly-herbal TCM formulation and WM combinations significantly improved outcomes such as the IPSS, maximum urine flow rate, prostate volume, and residual urine volume. More detailed information can be found in Supplementary Material S13.

## 4 Discussion

We performed the first network meta-analysis to evaluate the clinical efficacy of various poly-herbal TCM formulations combined with WM in the treatment of BPH. These treatments are recommended in clinical guidelines and are widely used in clinical practice. Distinct from previous traditional meta-analyses that focused on pairwise comparisons of poly-herbal TCM formulations and WM alone, we innovatively employed a Bayesian framework for model fitting in NMA, which allowed the comparison and ranking of multiple poly-herbal TCM formulations, thus providing significant reference value for clinical guidance.

The outcomes of our analysis indicated a superior clinical effective rate of JGSQ combined with WM. GZFL + WM demonstrated significant effectiveness in reducing IPSS scores by alleviating LUTS and enhancing maximum urinary flow rates. Moreover, PLA + WM showed notable efficacy in improving the QoL scores and reducing postvoid residual urine, while HE + WM effectively controlled prostate volume. Notably, the combination of poly-herbal TCM formulations with WM consistently demonstrated superior efficacy compared to WM alone.

TCM posits that BPH falls under the category of “retention of urine”, which encompasses symptoms like urinary obstruction or difficulty in urination (Sun et al., 2017). The etiology is often linked to dietary imbalance, external damp pathogen invasion, emotional disturbances, and deficiencies in internal organs. The primary deficiency is usually in the kidneys, resulting in disrupted qi transformation in the bladder, impaired circulation of qi and blood, and obstructed distribution of body fluids. Based on the pathogenesis, TCMs that possess efficacy to tonify the kidney, invigorate qi, activate blood, and promote diuresis are used for the treatment of BPH (Zhang et al., 2016; Yu, 2022; Zhang et al., 2023). The selected poly-herbal TCM formulations in this study all belong to this class of drugs.

Among several formulations that show favorable efficacy in this study, JGSQ is composed of 10 different traditional Chinese medicines, such as *Rehmannia glutinosa* (Gaertn.) DC. [Orobanchaceae], *Cornus officinalis* Siebold & Zucc. [Cornaceae], *Alisma orientale* (Sam.) Juz. [Alismataceae], and *Aconitum carmichaelii* Debeaux [Ranunculaceae]. These ingredients contain active compounds such as Rhmannioside D, paeonol, cornuside, aconitine, and alisol, which are crucial for the pharmacological effects of JGSQ, as indicated by studies on the pharmacological basis and mechanism of action (Yang, 2021). An animal study has shown that JGSQ can improve the compliance of the detrusor muscle in aged rats, increase bladder stress relaxation tension, and enhance detrusor muscle elasticity. JGSQ potentiates the relaxation effect of beta adrenergic receptor (β-AR) agonists and mitigates the inhibitory influence of beta-3 adrenergic receptor (β3-AR) antagonists. The potential mechanism of JGSQ may be related to the promotion of β3-AR-induced adenosine release inhibiting acetylcholine release in detrusor smooth fibers, thus facilitating bladder relaxation in aged rats (Silva et al., 2020). This enhances the maximum relaxation effect during the storage phase of the bladder, improves bladder filling, and reduces the frequency of urination. Additionally, JGSQ fortifies urethral sphincter closure, thereby preventing urine leakage and bolstering urinary control (Cao et al., 2015).

GZFL, a compound traditional Chinese medicine formulation refined through modern techniques, is composed of *Cinnamomum cassia* Presl [Lauraceae], *Poria cocos* (Schw.) Wolf [Polyporaceae], *Paeonia suffruticosa* Andrews [Paeoniaceae], *Prunus persica* (L.) Batsch [Rosaceae], and *Paeonia lactiflora* Pall. [Paeoniaceae], with possible active components like cinnamic acid, paeonol, and paeoniflorin (Yuan, 2022). Animal experiments have shown that GZFL capsules can inhibit prostate enlargement and glandular tissue expansion in rats, and modulate the proliferation of the glandular epithelium (Liu et al., 2004). Wang et al. investigated Guizhi Fuling capsules in BPH rat models, and indicated a significant reduction in prostate weight and prostate index, potentially through the modulation of dihydrotestosterone (DHT) levels in serum and prostate tissues, a decrease in vascular endothelial growth factor expression, and an elevation of transforming growth factor β1 (TGF-β1) levels, culminating in inhibited cellular proliferation and angiogenesis (Wang et al., 2021).

PLA tablets, also known as Qianliekang, are an extract of rapeseed pollen containing a range of bioactive enzymes, amino acids, and trace elements. Pollen extracts can inhibit the proliferation of epithelial and fibroblast cells in prostatic hyperplasia, with a specific selectivity for prostate cells (Habib et al., 1990; Wang et al., 1995). Animal models show that alcohol extracts induce bladder smooth muscle contraction in mice and aged rats and counteract norepinephrine-induced urethral smooth muscle contraction, thereby ameliorating urination. The underlying mechanism is hypothesized to involve alpha receptor blockade by these extracts (Kimura et al., 1986; Qian and Qian, 1990).

Huange capsules, a TCM aimed at energizing qi and stimulating blood circulation, are formulated from 12 traditional Chinese medicines such as *Astragalus membranaceus* (Fisch.) Bunge [Fabaceae], *P. persica* (L.) Batsch [Rosaceae], *Curcuma zedoaria* (Christm.) Roscoe [Zingiberaceae], and Coix lacryma-jobi var. ma-yuen (Rom.Caill.) Stapf [Poaceae]. The primary active components, including quercetin, kaempferol, and β-sitosterol (Guo et al., 2022), counteract α1 receptors, inhibit 5α-reductase activity, and decrease the production of basic fibroblast growth factor (bFGF). Furthermore, Huange capsules can reduce endothelin-1 (ET-1) expression in BPH rats, elevate nitric oxide synthase levels in prostate tissue, and suppress pathogenic gene expression, thus diminishing prostate smooth muscle tension, relaxing the muscle and inhibiting prostatic tissue hyperplasia, respectively (Geng, 2018).

In clinical practice, prostatectomy is the preferred treatment for patients with severe symptoms of BPH. However, for patients with milder and early-stage symptoms or those apprehensive about surgical complications and adverse reactions, oral medication is often the initial treatment choice. Poly-herbal TCM formulations have been widely used in China for many years and have achieved beneficial clinical effects. Additionally, TCM is now recognized as a complementary therapy in several other countries, including Germany and Switzerland. In the United States, states like Nevada and California have also garnered legislative support for TCM (Fan, 2015; Huang et al., 2022). Our results indicate that selected poly-herbal TCM formulations in combination with WM may cause specific adverse reactions, but these are well-tolerated and do not lead to severe adverse events. Nevertheless, caution should be exercised in medication administration, with prescriptions tailored according to symptoms.

This study has some limitations. Firstly, there was uncertainty regarding the estimates. Secondly, based on the results of the RoB2 tool, all included studies were evaluated as “some concern”, so caution is advised in interpreting the study results. Thirdly, despite retrieving 107 RCTs, some poly-herbal TCM formulations had a limited number of RCTs, and some trials had a small number of participants, potentially leading to biased results. Fourthly, the same poly-herbal TCM formulations included in this study may be produced by different manufacturers, with different manufacturing methods and dosage forms, resulting in inconsistent dosages. While the concentrations of active components are consistent according to *Pharmacopoeia of the People’s Republic of China*, some differences may exist. Additionally, apart from a few international RCTs, most of the studies included in this analysis focused on the Chinese population. Given the diversity of genetic backgrounds, caution is advised when evaluating and generalizing the efficacy of these findings to other ethnic populations. Further clinical studies worldwide and larger-scale randomized research are needed to validate these findings and enhance the applicability of the results. Nonetheless, the NOS results indicate that all the included studies are of high quality when they are regarded as cohort studies, and that the study findings still hold significant value to guide clinical medication practice.

## 5 Conclusion

In the conservative treatment of BPH, adding poly-herbal TCM formulations to WM could yield better therapeutic outcomes. JGSQ + WM, GZFL + WM, PLA + WM, and HE + WM warrant consideration in treating BPH. Further detailed evaluations are required in future high-quality clinical research.

## Data Availability

The original contributions presented in the study are included in the article/Supplementary Material, further inquiries can be directed to the corresponding authors.
