# Supplementary material for: Comparative efficacy of commercial oral poly-herbal traditional Chinese medicine formulations combined with western medicine in benign prostatic hyperplasia management: a systematic review and network meta-analysis
Source: Front Pharmacol. 2024 Jun 5;15:1358340. doi: 10.3389/fphar.2024.1358340 (PMC11187581; doi:10.3389/fphar.2024.1358340)
Supplement: Supplementary file 1 [file DataSheet1.docx]

Supplementary Material

[1 File S1: PRISMA checklist for network meta-analysis. 4](#_Toc163666871)

[2 File S2: Search strategy for network meta-analysis. 8](#_Toc163666872)

[2.1 Search strategy of China National Knowledge Infrastructure. 8](#_Toc163666873)

[2.2 Search strategy of Wanfang Database. 8](#_Toc163666874)

[2.3 Search strategy of Chinese Biomedical Literature Database. 9](#_Toc163666875)

[2.4 Search strategy of Weipu Journal Database. 9](#_Toc163666876)

[2.5 Search strategy of Pubmed. 10](#_Toc163666877)

[2.6 Search strategy of Embase. 11](#_Toc163666878)

[2.7 Search strategy of Cochrane Library. 11](#_Toc163666879)

[2.8 Search strategy of Web of Science. 12](#_Toc163666880)

[2.9 Search strategy of Ovid-Medline. 13](#_Toc163666881)

[2.10 Search strategy of ClinicalTrials.gov. 13](#_Toc163666882)

[3 File S3: Citations of the selected studies. 15](#_Toc163666883)

[4 File S4: Details of the included poly-herbal TCM formulations. 24](#_Toc163666884)

[4.1 Plant material and initial processing of poly-herbal TCM formulations. 24](#_Toc163666885)

[4.2 Analytical methods for the chemical profile of poly-herbal TCM formulations according to ConPhyMP. 34](#_Toc163666886)

[5 File S5: Details of the included studies. 54](#_Toc163666887)

[6 File S6: Quality assessment of the included studies. 73](#_Toc163666888)

[6.1 Summary of RoB2 assessment. 73](#_Toc163666889)

[6.2 RoB2 assessment of each included study. 73](#_Toc163666890)

[6.3 NOS assessment of each included study. 77](#_Toc163666891)

[7 File S7: Brooks-Gelman-Rubin plots. 79](#_Toc163666892)

[7.1 Brooks-Gelman-Rubin plots for clinical effective rate. 79](#_Toc163666893)

[7.2 Brooks-Gelman-Rubin plots for IPSS. 80](#_Toc163666894)

[7.3 Brooks-Gelman-Rubin plots for QoL score. 81](#_Toc163666895)

[7.4 Brooks-Gelman-Rubin plots for maximum flow rate. 82](#_Toc163666896)

[7.5 Brooks-Gelman-Rubin plots for the prostate volume. 83](#_Toc163666897)

[7.6 Brooks-Gelman-Rubin plots for postvoid residual urine volume. 84](#_Toc163666898)

[8 File S8: Table of adverse drugs reactions. 86](#_Toc163666899)

[9 File S9: Final result and goodness of fit of the models for each outcome. 87](#_Toc163666900)

[10 File S10: Heterogeneity detection. 88](#_Toc163666901)

[10.1 Predictive interval plot for clinical effective rate. 88](#_Toc163666902)

[10.2 Predictive interval plot for IPSS. 89](#_Toc163666903)

[10.3 Predictive interval plot for QoL score. 90](#_Toc163666904)

[10.4 Predictive interval plot for maximum flow rate of urine. 91](#_Toc163666905)

[10.5 Predictive interval plot for prostate volume. 92](#_Toc163666906)

[10.6 Predictive interval plot for postvoid residual urine. 94](#_Toc163666907)

[11 File S11: Funnel plots for publication bias. 95](#_Toc163666908)

[12 File S12: Sensitivity analyses. 96](#_Toc163666909)

[12.1 Sensitivity analysis within a network framework focusing on studies published in the past decade. 96](#_Toc163666910)

[12.2 Sensitivity analysis within a network framework considering studies that exclude poly-herbal TCM formulations with fewer than three selected RCTs in each outcome. 98](#_Toc163666911)

[12.3 Sensitivity analysis within a network framework considering studies of poly-herbal TCM formulations with consistent dosage and usage. 100](#_Toc163666912)

[13 File S13: Subgroup network meta-analysis. 103](#_Toc163666913)

[13.1 Subgroup network meta-analysis for clinical effective rate 103](#_Toc163666914)

[13.2 Subgroup network meta-analysis for IPSS 104](#_Toc163666915)

[13.3 Subgroup network meta-analysis for QoL score 105](#_Toc163666916)

[13.4 Subgroup network meta-analysis for maximum flow rate of urine 106](#_Toc163666917)

[13.5 Subgroup network meta-analysis for prostate volume 107](#_Toc163666918)

[13.6 Subgroup network meta-analysis for postvoid residual urine 108](#_Toc163666919)

# File S1: PRISMA checklist for network meta-analysis.

| **Section/topic** | **#** | **Checklist item** | **Reported on page #** |
| --- | --- | --- | --- |
| **TITLE** | | |  |
| Title | 1 | Identify the report as a systematic review incorporating a network meta-analysis (or related form of meta-analysis). |  |
| **ABSTRACT** | | |  |
| Structured summary | 2 | Provide a structured summary including, as applicable:  Background: main objectives  Methods: data sources; study eligibility criteria, participants, and interventions; study appraisal; and synthesis methods, such as network meta-analysis.  Results: number of studies and participants identified; summary estimates with corresponding confidence/credible intervals; treatment rankings may also be discussed. Authors may choose to summarize pairwise comparisons against a chosen treatment included in their analyses for brevity.  Discussion/Conclusions: limitations; conclusions and implications of findings.  Other: primary source of funding; systematic review registration number with registry name. |  |
| **INTRODUCTION** | | |  |
| Rationale | 3 | Describe the rationale for the review in the context of what is already known, including mention of why a network meta-analysis has been conducted. |  |
| Objectives | 4 | Provide an explicit statement of questions being addressed with reference to participants, interventions, comparisons, outcomes, and study design (PICOS). |  |
| **METHODS** | | |  |
| Protocol and registration | 5 | Indicate if a review protocol exists and if and where it can be accessed (e.g., Web address), and, if available, provide registration information including registration number. |  |
| Eligibility criteria | 6 | Specify study characteristics (e.g., PICOS, length of follow-up) and report characteristics (e.g., years considered, language, publication status) used as criteria for eligibility, giving rationale. Clearly describe eligible treatments included in the treatment network and note whether any have been clustered or merged into the same node (with justification). |  |
| Information sources | 7 | Describe all information sources (e.g., databases with dates of coverage, contact with study authors to identify additional studies) in the search and date last searched. |  |
| Search | 8 | Present full electronic search strategy for at least one database, including any limits used, such that it could be repeated. |  |
| Study selection | 9 | State the process for selecting studies (i.e., screening, eligibility, included in systematic review, and, if applicable, included in the meta-analysis). |  |
| Data collection process | 10 | Describe method of data extraction from reports (e.g., piloted forms, independently, in duplicate) and any processes for obtaining and confirming data from investigators. |  |
| Data items | 11 | List and define all variables for which data were sought (e.g., PICOS, funding sources) and any assumptions and simplifications made. |  |
| Geometry of the network | S1 | Describe methods used to explore the geometry of the treatment network under study and potential biases related to it. This should include how the evidence base has been graphically summarized for presentation, and what characteristics were compiled and used to describe the evidence base to readers |  |
| Risk of bias within individual studies | 12 | Describe methods used for assessing risk of bias of individual studies (including specification of whether this was done at the study or outcome level), and how this information is to be used in any data synthesis. |  |
| Summary measures | 13 | State the principal summary measures (e.g., risk ratio, difference in means). Also describe the use of additional summary measures assessed, such as treatment rankings and surface under the cumulative ranking curve (SUCRA) values, as well as modified approaches used to present summary findings from meta-analyses. |  |
| Planned methods of analysis | 14 | Describe the methods of handling data and combining results of studies for each network meta-analysis. This should include, but not be limited to: Handling of multigroup trials; Selection of variance structure; Selection of prior distributions in Bayesian analyses; and Assessment of model fit. |  |
| Assessment of inconsistency | S2 | Describe the statistical methods used to evaluate the agreement of direct and indirect evidence in the treatment network(s) studied. Describe efforts taken to address its presence when found. |  |
| Risk of bias across studies | 15 | Specify any assessment of risk of bias that may affect the cumulative evidence (e.g., publication bias, selective reporting within studies) |  |
| Additional analyses | 16 | Describe methods of additional analyses if done, indicating which were prespecified. This may include, but not be limited to, the following: Sensitivity or subgroup analyses; Meta-regression analyses; Alternative formulations of the treatment network; and Use of alternative prior distributions for Bayesian analyses (if applicable). |  |
| **RESULTS** | | |  |
| Study selection | 17 | Give numbers of studies screened, assessed for eligibility, and included in the review, with reasons for exclusions at each stage, ideally with a flow diagram. |  |
| Presentation of network structure | S3 | Provide a network graph of the included studies to enable visualization of the geometry of the treatment network. |  |
| Summary of network geometry | S4 | Provide a brief overview of characteristics of the treatment network. This may include commentary on the abundance of trials and randomized patients for the different interventions and pairwise comparisons in the network, gaps of evidence in the treatment network, and potential biases reflected by the network structure. |  |
| Study characteristics | 18 | For each study, present characteristics for which data were extracted (e.g., study size, PICOS, follow-up period) and provide the citations. |  |
| Risk of bias within studies | 19 | Present data on risk of bias of each study and, if available, any outcome level assessment. |  |
| Results of individual studies | 20 | For all outcomes considered (benefits or harms), present, for each study: 1) simple summary data for each intervention group, and 2) effect estimates and confidence intervals. Modified approaches may be needed to deal with information from larger networks. |  |
| Synthesis of results | 21 | Present results of each meta-analysis done, including confidence/credible intervals. In larger networks, authors may focus on comparisons versus a particular comparator (e.g., placebo or standard care), with full findings presented in an appendix. League tables and forest plots may be considered to summarize pairwise comparisons. If additional summary measures were explored (such as treatment rankings), these should also be presented. |  |
| Exploration for inconsistency | S5 | Describe results from investigations of inconsistency. This may include such information as measures of model fit to compare consistency and inconsistency models, P values from statistical tests, or summary of inconsistency estimates from different parts of the treatment network. |  |
| Risk of bias across studies | 22 | Present results of any assessment of risk of bias across studies for the evidence base being studied. |  |
| Results of additional analyses | 23 | Give results of additional analyses, if done (e.g., sensitivity or subgroup analyses, meta-regression  analyses, alternative network geometries studied, alternative choice of prior distributions for  Bayesian analyses, and so forth). |  |
| **DISCUSSION** | | |  |
| Summary of evidence | 24 | Summarize the main findings, including the strength of evidence for each main outcome; consider their relevance to key groups (e.g., health care providers, researchers, and policymakers). |  |
| Limitations | 25 | Discuss limitations at study and outcome level (e.g., risk of bias), and at review level (e.g., incomplete retrieval of identified research, reporting bias). Comment on the validity of the assumptions, such as transitivity and consistency. Comment on any concerns regarding network geometry (e.g., avoidance of certain comparisons). |  |
| Conclusions | 26 | Provide a general interpretation of the results in the context of other evidence, and implications for future research. |  |
| **FUNDING** | | |  |
| Funding | 27 | Describe sources of funding for the systematic review and other support (e.g., supply of data); role of funders for the systematic review. This should also include information regarding whether funding has been received from manufacturers of treatments in the network and/or whether some of the authors are content experts with professional conflicts of interest that could affect use of treatments in the network. |  |

# File S2: Search strategy for network meta-analysis.

## Search strategy of China National Knowledge Infrastructure.

| No. | Search items |
| --- | --- |
| #1 | SU %= '前列腺增生' OR SU %= '良性前列腺增生' OR SU %= '前列腺肥大' OR SU %= '良性前列腺增生症' OR SU %= '良性增生前列腺' OR SU %= '慢性前列腺增生' |
| #2 | SU %= '中成药' OR SU %= '前列康' OR SU %= '右归胶囊' OR SU %= '金匮肾气丸' OR SU %= '前列欣胶囊' OR SU %= '前列舒通胶囊' OR SU %= '锯叶棕' OR SU %= '癃清片' OR SU %= '龙金通淋胶囊' OR SU %= '黄莪胶囊' OR SU %= '灵泽片' OR SU %= '强肾片' OR SU %= '金水宝胶囊' OR SU %= '补中益气丸' OR SU %= '宁泌泰胶囊' OR SU %= '舒泌通胶囊' OR SU %= '桂枝茯苓胶囊' OR SU %= '前列倍喜胶囊' OR SU %= '复方雪参胶囊' OR SU %= '泽桂癃爽胶囊' OR SU %= '济生肾气丸' OR SU %= '八正胶囊' OR SU %= '前列通瘀胶囊' OR SU %= '知柏地黄丸' OR SU %= '左归丸' OR SU %= '右归丸' OR SU %= '龟龄集' OR SU %= '六味地黄丸' OR SU %= '滋肾通关丸' OR SU %= '翁沥通胶囊' OR SU %= '夏荔芪胶囊' OR SU %= '前列金丹片' OR SU %= '前列舒丸' OR SU %= '癃闭舒胶囊' |
| #3 | FT = '随机' |
| #4 | #1 AND #2 AND #3 |

## Search strategy of Wanfang Database.

| No. | Search items |
| --- | --- |
| #1 | 主题:(前列腺增生) or 主题:(良性前列腺增生) or 主题:(前列腺肥大) or 主题:(良性前列腺增生症) or 主题:(良性增生前列腺) or 主题:(慢性前列腺增生) |
| #2 | 主题:(中成药) or 主题:(前列康) or 主题:(右归胶囊) or 主题:(金匮肾气丸) or 主题:(前列欣胶囊) or 主题:(前列舒通胶囊) or 主题:(锯叶棕) or 主题:(癃清片) or 主题:(龙金通淋胶囊) or 主题:(黄莪胶囊) or 主题:(灵泽片) or 主题:(强肾片) or 主题:(金水宝胶囊) or 主题:(补中益气丸) or 主题:(宁泌泰胶囊) or 主题:(舒泌通胶囊) or 主题:(桂枝茯苓胶囊) or 主题:(前列倍喜胶囊) or 主题:(复方雪参胶囊) or 主题:(泽桂癃爽胶囊) or 主题:(济生肾气丸) or 主题:(八正胶囊) or 主题:(前列通瘀胶囊) or 主题:(知柏地黄丸) or 主题:(左归丸) or 主题:(右归丸) or 主题:(龟龄集) or 主题:(六味地黄丸) or 主题:(滋肾通关丸) or 主题:(翁沥通胶囊) or 主题:(夏荔芪胶囊) or 主题:(前列金丹片) or 主题:(前列舒丸) or 主题:(癃闭舒胶囊) |
| #3 | 全部:(随机) |
| #4 | #1 AND #2 AND #3 |

## Search strategy of Chinese Biomedical Literature Database.

| No. | Search items |
| --- | --- |
| #1 | "前列腺增生"[常用字段:智能] OR "良性前列腺增生"[常用字段:智能] OR "前列腺肥大"[常用字段:智能] OR "良性前列腺增生症"[常用字段:智能] OR "良性增生前列腺"[常用字段:智能] OR "慢性前列腺增生"[常用字段:智能] |
| #2 | "中成药"[常用字段:智能] OR "前列康"[常用字段:智能] OR "右归胶囊"[常用字段:智能] OR "金匮肾气丸"[常用字段:智能] OR "前列欣胶囊"[常用字段:智能] OR "前列舒通胶囊"[常用字段:智能] OR "锯叶棕"[常用字段:智能] OR "癃清片"[常用字段:智能] OR "龙金通淋胶囊"[常用字段:智能] OR "黄莪胶囊"[常用字段:智能] OR "灵泽片"[常用字段:智能] OR "强肾片"[常用字段:智能] OR "金水宝胶囊"[常用字段:智能] OR "补中益气丸"[常用字段:智能] OR "宁泌泰胶囊"[常用字段:智能] OR "舒泌通胶囊"[常用字段:智能] OR "桂枝茯苓胶囊"[常用字段:智能] OR "前列倍喜胶囊"[常用字段:智能] OR "复方雪参胶囊"[常用字段:智能] OR "泽桂癃爽胶囊"[常用字段:智能] OR "济生肾气丸"[常用字段:智能] OR "八正胶囊"[常用字段:智能] OR "前列通瘀胶囊"[常用字段:智能] OR "知柏地黄丸"[常用字段:智能] OR "左归丸"[常用字段:智能] OR "右归丸"[常用字段:智能] OR "龟龄集"[常用字段:智能] OR "六味地黄丸"[常用字段:智能] OR "滋肾通关丸"[常用字段:智能] OR "翁沥通胶囊"[常用字段:智能] OR "夏荔芪胶囊"[常用字段:智能] OR "前列金丹片"[常用字段:智能] OR "前列舒丸"[常用字段:智能] OR "癃闭舒胶囊"[常用字段:智能] |
| #3 | "随机"[全部字段:智能]) |
| #4 | #1 AND #2 AND #3 |

## Search strategy of Weipu Journal Database.

| No. | Search items |
| --- | --- |
| #1 | M=(前列腺增生 OR 良性前列腺增生 OR 前列腺肥大 OR 良性前列腺增生症 OR 良性增生前列腺 OR 慢性前列腺增生) |
| #2 | M=(中成药 OR 前列康 OR 右归胶囊 OR 金匮肾气丸 OR 前列欣胶囊 OR 前列舒通胶囊 OR 锯叶棕 OR 癃清片 OR 龙金通淋胶囊 OR 黄莪胶囊 OR 灵泽片 OR 强肾片 OR 金水宝胶囊 OR 补中益气丸 OR 宁泌泰胶囊 OR 舒泌通胶囊 OR 桂枝茯苓胶囊 OR 前列倍喜胶囊 OR 复方雪参胶囊 OR 泽桂癃爽胶囊 OR 济生肾气丸 OR 八正胶囊 OR 前列通瘀胶囊 OR 知柏地黄丸 OR 左归丸 OR 右归丸 OR 龟龄集 OR 六味地黄丸 OR 滋肾通关丸 OR 翁沥通胶囊 OR 夏荔芪胶囊 OR 前列金丹片 OR 前列舒丸 OR 癃闭舒胶囊) |
| #3 | U=(随机) |
| #4 | #1 AND #2 AND #3 |

## Search strategy of Pubmed.

| No. | Search items |
| --- | --- |
| #1 | "prostatic hyperplasia"[MeSH Terms] |
| #2 | Hyperplasia, Prostatic[Title/Abstract] OR Adenoma, Prostatic[Title/Abstract] OR Adenomas, Prostatic[Title/Abstract] OR Prostatic Adenomas[Title/Abstract] OR Prostatic Adenoma[Title/Abstract] OR Benign Prostatic Hyperplasia[Title/Abstract] OR Benign Prostatic Hyperplasias[Title/Abstract] OR Hyperplasia, Benign Prostatic[Title/Abstract] OR Hyperplasias, Benign Prostatic[Title/Abstract] OR Prostatic Hyperplasias, Benign[Title/Abstract] OR Prostatic Hypertrophy, Benign[Title/Abstract] OR Benign Prostatic Hypertrophy[Title/Abstract] OR Hypertrophy, Benign Prostatic[Title/Abstract] OR Prostatic Hyperplasia, Benign[Title/Abstract] OR Prostatic Hypertrophy[Title/Abstract] OR Hypertrophies, Prostatic[Title/Abstract] OR Hypertrophy, Prostatic[Title/Abstract] OR Prostatic Hypertrophies[Title/Abstract] |
| #3 | #1 OR #2 |
| #4 | Chinese patent medicine[Title/Abstract] OR Qianliekang[Title/Abstract] OR Yougui[Title/Abstract] OR Jinguishenqi[Title/Abstract] OR Qianliexin[Title/Abstract] OR Qianlieshutong[Title/Abstract] OR Saw palmetto[Title/Abstract] OR Serenoa repens[Title/Abstract] OR Longqing[Title/Abstract] OR Longjintonglin[Title/Abstract] OR Huange[Title/Abstract] OR Lingze[Title/Abstract] OR Qiangshen[Title/Abstract] OR Jinshuibao[Title/Abstract] OR Buzhongyiqi[Title/Abstract] OR Ningmitai[Title/Abstract] OR Shumitong[Title/Abstract] OR Guizhifuling[Title/Abstract] OR Qianliebeixi[Title/Abstract] OR Fufangxueshen[Title/Abstract] OR Zeguilongshuang[Title/Abstract] OR Jishengshenqi[Title/Abstract] OR Bazheng[Title/Abstract] OR Qianlietongyu[Title/Abstract] OR Zhibaidihuang[Title/Abstract] OR Zuogui[Title/Abstract] OR Guilingji[Title/Abstract] OR Liuweidihuang[Title/Abstract] OR Zishentongguan[Title/Abstract] OR Wenglitong[Title/Abstract] OR Xialiqi[Title/Abstract] OR Qianliejindan[Title/Abstract] OR Qianlieshu[Title/Abstract] OR Longbishu[Title/Abstract] |
| #5 | Controlled Clinical Trial [Publication Type] OR Randomized Controlled Trial[Publication Type] OR Equivalence Trial[Publication Type] OR Pragmatic Clinical Trial[Publication Type] OR random*[All Fields] |
| #6 | #3 AND #4 AND #5 |

## Search strategy of Embase.

| No. | Search items |
| --- | --- |
| #1 | 'prostate hypertrophy'/exp OR 'hyperplasia, prostatic':ti,ab,kw OR 'adenoma, prostatic':ti,ab,kw OR 'adenomas, prostatic':ti,ab,kw OR 'prostatic adenomas':ti,ab,kw OR 'prostatic adenoma':ti,ab,kw OR 'benign prostatic hyperplasia':ti,ab,kw OR 'benign prostatic hyperplasias':ti,ab,kw OR 'hyperplasia, benign prostatic':ti,ab,kw OR 'hyperplasias, benign prostatic':ti,ab,kw OR 'prostatic hyperplasias, benign':ti,ab,kw OR 'prostatic hypertrophy, benign':ti,ab,kw OR 'benign prostatic hypertrophy':ti,ab,kw OR 'hypertrophy, benign prostatic':ti,ab,kw OR 'prostatic hyperplasia, benign':ti,ab,kw OR 'prostatic hypertrophy':ti,ab,kw OR 'hypertrophies, prostatic':ti,ab,kw OR 'hypertrophy, prostatic':ti,ab,kw OR 'prostatic hypertrophies' |
| #2 | 'chinese patent medicine':ti,ab,kw OR qianliekang:ti,ab,kw OR yougui:ti,ab,kw OR Jinguishenqi:ti,ab,kw OR qianliexin:ti,ab,kw OR qianlieshutong:ti,ab,kw OR 'saw palmetto':ti,ab,kw OR 'serenoa repens':ti,ab,kw OR longqing:ti,ab,kw OR longjintonglin:ti,ab,kw OR huange:ti,ab,kw OR lingze:ti,ab,kw OR qiangshen:ti,ab,kw OR jinshuibao:ti,ab,kw OR buzhongyiqi:ti,ab,kw OR ningmitai:ti,ab,kw OR shumitong:ti,ab,kw OR guizhifuling:ti,ab,kw OR qianliebeixi:ti,ab,kw OR fufangxueshen:ti,ab,kw OR zeguilongshuang:ti,ab,kw OR jishengshenqi:ti,ab,kw OR bazheng:ti,ab,kw OR qianlietongyu:ti,ab,kw OR zhibaidihuang:ti,ab,kw OR zuogui:ti,ab,kw OR guilingji:ti,ab,kw OR liuweidihuang:ti,ab,kw OR zishentongguan:ti,ab,kw OR wenglitong:ti,ab,kw OR xialiqi:ti,ab,kw OR qianliejindan:ti,ab,kw OR qianlieshu:ti,ab,kw OR longbishu:ti,ab,kw |
| #3 | 'randomized controlled trial'/exp OR 'equivalence trial'/exp OR 'non-inferiority trial'/exp OR 'pragmatic trial'/exp OR 'superiority trial'/exp OR 'controlled clinical trial':it OR 'randomized controlled trial':it OR 'equivalence trial':it OR 'pragmatic clinical trial':it OR 'superiority trial':it OR 'non-inferiority trial':it OR random* |
| #4 | #1 AND #2 AND #3 |

## Search strategy of Cochrane Library.

| No. | Search items |
| --- | --- |
| #1 | MeSH descriptor: [Prostatic Hyperplasia] explode all trees |
| #2 | (Hyperplasia, Prostatic):ti,ab,kw OR (Adenoma, Prostatic):ti,ab,kw OR (Adenomas, Prostatic):ti,ab,kw OR (Prostatic Adenomas):ti,ab,kw OR (Prostatic Adenoma):ti,ab,kw OR (Benign Prostatic Hyperplasia):ti,ab,kw OR (Benign Prostatic Hyperplasias):ti,ab,kw OR (Hyperplasia, Benign Prostatic):ti,ab,kw OR (Hyperplasias, Benign Prostatic):ti,ab,kw OR (Prostatic Hyperplasias, Benign):ti,ab,kw OR (Prostatic Hypertrophy, Benign):ti,ab,kw OR (Benign Prostatic Hypertrophy):ti,ab,kw OR (Hypertrophy, Benign Prostatic):ti,ab,kw OR (Prostatic Hyperplasia, Benign):ti,ab,kw OR (Prostatic Hypertrophy):ti,ab,kw OR (Hypertrophies, Prostatic):ti,ab,kw OR (Hypertrophy, Prostatic):ti,ab,kw OR (Prostatic Hypertrophies):ti,ab,kw |
| #3 | #1 OR #2 |
| #4 | (Chinese patent medicine):ti,ab,kw OR (Qianliekang):ti,ab,kw OR (Yougui):ti,ab,kw OR (Jinguishenqi):ti,ab,kw OR (Qianliexin):ti,ab,kw OR (Qianlieshutong):ti,ab,kw OR (Saw palmetto):ti,ab,kw OR (Serenoa repens):ti,ab,kw OR (Longqing):ti,ab,kw OR (Longjintonglin):ti,ab,kw OR (Huange):ti,ab,kw OR (Lingze):ti,ab,kw OR (Qiangshen):ti,ab,kw OR (Jinshuibao):ti,ab,kw OR (Buzhongyiqi):ti,ab,kw OR (Ningmitai):ti,ab,kw OR (Shumitong):ti,ab,kw OR (Guizhifuling):ti,ab,kw OR (Qianliebeixi):ti,ab,kw OR (Fufangxueshen):ti,ab,kw OR (Zeguilongshuang):ti,ab,kw OR (Jishengshenqi):ti,ab,kw OR (Bazheng):ti,ab,kw OR (Qianlietongyu):ti,ab,kw OR (Zhibaidihuang):ti,ab,kw OR (Zuogui):ti,ab,kw OR (Guilingji):ti,ab,kw OR (Liuweidihuang):ti,ab,kw OR (Zishentongguan):ti,ab,kw OR (Wenglitong):ti,ab,kw OR (Xialiqi):ti,ab,kw OR (Qianliejindan):ti,ab,kw OR (Qianlieshu):ti,ab,kw OR (Longbishu):ti,ab,kw |
| #5 | MeSH descriptor: [Randomized Controlled Trial] explode all trees |
| #6 | (Randomized Controlled Trial):pt OR (Controlled Clinical Trial):pt OR (Equivalence Trial):pt OR (Pragmatic Clinical Trial):pt OR (random*) |
| #7 | #5 OR #6 |
| #8 | #3 AND #4 AND #7 |

## Search strategy of Web of Science.

| No. | Search items |
| --- | --- |
| #1 | Hyperplasia, Prostatic (主题) or Adenoma, Prostatic (主题) or Adenomas, Prostatic (主题) or Prostatic Adenomas (主题) or Prostatic Adenoma (主题) or Benign Prostatic Hyperplasia (主题) or Benign Prostatic Hyperplasias (主题) or Hyperplasia, Benign Prostatic (主题) or Hyperplasias, Benign Prostatic (主题) or Prostatic Hyperplasias, Benign (主题) or Prostatic Hypertrophy, Benign (主题) or Benign Prostatic Hypertrophy (主题) or Hypertrophy, Benign Prostatic (主题) or Prostatic Hyperplasia, Benign (主题) or Prostatic Hypertrophy (主题) or Hypertrophies, Prostatic (主题) or Hypertrophy, Prostatic (主题) or Prostatic Hypertrophies (主题) |
| #2 | Chinese patent medicine (主题) or Qianliekang (主题) or Yougui (主题) or Jinguishenqi (主题) or Qianliexin (主题) or Qianlieshutong (主题) or Saw palmetto (主题) or Serenoa repens (主题) or Longqing (主题) or Longjintonglin (主题) or Huange (主题) or Lingze (主题) or Qiangshen (主题) or Jinshuibao (主题) or Buzhongyiqi (主题) or Ningmitai (主题) or Shumitong (主题) or Guizhifuling (主题) or Qianliebeixi (主题) or Fufangxueshen (主题) or Zeguilongshuang (主题) or Jishengshenqi (主题) or Bazheng (主题) or Qianlietongyu (主题) or Zhibaidihuang (主题) or Zuogui (主题) or Guilingji (主题) or Liuweidihuang (主题) or Zishentongguan (主题) or Wenglitong (主题) or Xialiqi (主题) or Qianliejindan (主题) or Qianlieshu (主题) or Longbishu (主题) |
| #3 | random* (全部) |
| #4 | #1 AND #2 AND #3 |

## Search strategy of Ovid-Medline.

| No. | Search items |
| --- | --- |
| #1 | exp Prostatic Hyperplasia/ or prostate hyperplasia.mp. or (Hyperplasia, Prostatic or Adenoma, Prostatic or Adenomas, Prostatic or Prostatic Adenomas or Prostatic Adenoma or Benign Prostatic Hyperplasia or Benign Prostatic Hyperplasias or Hyperplasia, Benign Prostatic or Hyperplasias, Benign Prostatic or Prostatic Hyperplasias, Benign or Prostatic Hypertrophy, Benign or Benign Prostatic Hypertrophy or Hypertrophy, Benign Prostatic or Prostatic Hyperplasia, Benign or Prostatic Hypertrophy or Hypertrophies, Prostatic or Hypertrophy, Prostatic or Prostatic Hypertrophies).af. |
| #2 | Chinese patent medicine.mp. or (Qianliekang or Yougui or Jinguishenqi or Qianliexin or Qianlieshutong or Saw palmetto or Serenoa repens or Longqing or Longjintonglin or Huange or Lingze or Qiangshen or Jinshuibao or Buzhongyiqi or Ningmitai or Shumitong or Guizhifuling or Qianliebeixi or Fufangxueshen or Zeguilongshuang or Jishengshenqi or Bazheng or Qianlietongyu or Zhibaidihuang or Zuogui or Guilingji or Liuweidihuang or Zishentongguan or Wenglitong or Xialiqi or Qianliejindan or Qianlieshu or Longbishu).af. |
| #3 | randomized controlled trial.mp. or exp Randomized Controlled Trial/ or random*.mp. |
| #4 | #1 AND #2 AND #3 |

## Search strategy of ClinicalTrials.gov.

| No. | Search items |
| --- | --- |
| #1 | prostate hyperplasia OR prostatic hyperplasia OR prostatic hypertrophy OR prostate hypertrophy |
| #2 | Chinese patent medicine OR Qianliekang OR Yougui OR Jinguishenqi OR Qianliexin OR Qianlieshutong OR Saw palmetto OR Serenoa repens OR Longqing OR Longjintonglin OR Huange OR Lingze OR Qiangshen OR Jinshuibao OR Buzhongyiqi OR Ningmitai OR Shumitong OR Guizhifuling OR Qianliebeixi OR Fufangxueshen OR Zeguilongshuang OR Jishengshenqi OR Bazheng OR Qianlietongyu OR Zhibaidihuang OR Zuogui OR Guilingji OR Liuweidihuang OR Zishentongguan OR Wenglitong OR Xialiqi OR Qianliejindan OR Qianlieshu OR Longbishu |
| #3 | limit to Interventional Studies and “Study with results” |
| #4 | #1 AND #2 AND #3 |

# File S3: Citations of the selected studies.

Argirović, A., and Argirović, D. (2013). Does the addition of Serenoa repens to tamsulosin improve its therapeutical efficacy in benign prostatic hyperplasia? *Vojnosanitetski pregled* 70, 1091–6. doi: 10.2298/vsp110620029a.

Chang, D. G., Li, G. S., Peng, C. H., Yu, X. J., Zhang, P. H., Bi, M. S., et al. (2015). Longbishu Capsules combined with mesylate doxazosin: An efficacious therapy for benign prostatic hyperplasia. *National Journal of Andrology* 21, 165–169. doi: 10.13263/j.cnki.nja.2015.02.014.

Chang, J. K., Huo, J. Q., Zhu, Z. Y., Xu, W. B., Zhao, X. L., Zhao, Z. H., et al. (2020). Clinical study on Qianlieshu Pills combined with silodosin in treatment of prostatic hyperplasia. *Drugs & Clinic* 35, 1671–1674.

Chen, H., Liang, T. F., and Huang, Q. Q. (2014). Effect of Half-dose Jingui Shenqi Pill on the Symptoms of Nocturia and Sex Hormones in Prostatic Hyperplasia. *Guangdong Medical Journal* 35, 2280–2281. doi: 10.13820/j.cnki.gdyx.2014.14.056.

Chen, H., Wang, S. L., Zhu, H. P., Zeng, H., and Yan, Z. N. (2013). Clinical Study on the Combination of Jingui Shenqi Pill and Conventional Western Medicine in the Treatment of Benign Prostatic Hyperplasia. *Chinese Journal of Clinical Rational Drug Use* 6, 47–49. doi: 10.15887/j.cnki.13-1389/r.2013.35.121.

Chen, H., Zhang, R., Huang, X. R., Xin, Z. Q., and Liang, Z. Y. (2016). Study on the Optimization of the Combination of Chinese and Western Medicine in the Treatment of Benign Prostatic Hyperplasia. *World Journal of Integrated Traditional and Western Medicine* 11, 510–513. doi: 10.13935/j.cnki.sjzx.160420.

Chen, M. X. (2008). Effect of Combining Terazosin and Zegui Longshuang on the Treatment of Benign Prostatic Hyperplasia. *Journal of Hainan Medical University*, 697–699. doi: 10.13210/j.cnki.jhmu.2008.06.045.

Chen, X. S., Ou, T. W., and Zhang, J. (2017). The Therapeutic Effect of Longbishu Capsule Combined with Western Medicine in the Treatment of Benign Prostatic Hyperplasia and Its Impact on Traditional Chinese Medicine Syndrome Score and Qol Score. *Modern Journal of Integrated Traditional Chinese and Western Medicine* 26, 4072–4074.

Cheng, W. (2023). Clinical Study on Huange Capsules Combined with Western Medicine for Benign Prostatic Hyperplasia. *NEW CHINESE MEDICINE* 55, 66–70. doi: 10.13457/j.cnki.jncm.2023.18.014.

Di Maida, F., Mari, A., Rubino, R., Minervini, A., Carini, M., and Siena, G. (2020). A Prospective, Open-Label Comparison of Tamsulosin plus Serenoa repens and Bovine Colostrum versus Tamsulosin Alone in the Treatment of Benign Prostatic Hyperplasia. *Urologia internationalis* 104, 351–355. doi: 10.1159/000503735.

Du, X. (2016). Efficacy of BPH Capsule Qianlieshu Joint Finasteride Therapy. *Guide of China Medicine* 14, 99–100. doi: 10.15912/j.cnki.gocm.2016.21.086.

Duan, Z. X., and Liang, C. Y. (2020). The Effect of Qianlie Shutong Capsules Combined with Tamsulosin in the Treatment of Benign Prostatic Hyperplasia on Urethral Function in Patients. *China Rural Health* 12, 22.

Fu, Q. Y., and Lou, G. (2016). Clinical Observation on the Combined Treatment and Nursing Intervention of Traditional Chinese and Western Medicine for Benign Prostatic Hyperplasia. *NEW CHINESE MEDICINE* 48, 184–185. doi: 10.13457/j.cnki.jncm.2016.12.078.

Fu, X. J., Hu, Q. H., Cheng, J. R., Li, F. H., and Sun, J. Y. (2015). Effect of Qianlie Shutong Capsule Combined with Phenoxybenzamine for Treating Elderly Patients with Benign Prostatic Hyperplasia in 74 Cases. *China Pharmaceuticals* 24, 34–35.

Gao, J. R. (2014). Observation on the Efficacy of Qianliekang Capsules in the Treatment of Mild Prostatic Hyperplasia. *The Medical Forum* 18, 1394–1395.

Gao, K., and Liu, H. (2016). Clinical Analysis of the Combination of Phenoxybenzamine and Qianlie Shutong Capsules in the Treatment of Benign Prostate Hyperplasia in the Elderly. *Health Guide*, 23. doi: 10.3969/j.issn.1006-6845.2016.51.022.

Gao, W., and Xue, X. T. (2022). Effect of Prostate Dredging Capsule Combined with Epprete on Symptom Score and Serum T and E2 in Patients with Prostatic Hyperplasia. *Reflexology and Rehabilitation Medicine* 3, 140–143.

Gao, Z., Lun, L. J., SHAO, K. Q., Shen, J. W., LI, Q., and Zhang, J. Y. (2011). Clinical Observation on Qian Lie Xin Capsule Plus Tamsulosin Hydrochloride Sustained Release Capsules in the Treatment of Benign Prostatic Hyperplasia with Shi Re Yu Zu Syndrome. *Journal of Hunan University of Chinese Medicine* 31, 17–20.

Ge, H. Y., Chen, B., Liu, H. L., and Li, H. Q. (2011). The Clinical Observation of Tamsulosin Combined with Zhi Bai Di Huang Pill for the Treatment of Benign Prostatic Hyperplasia in the Elderly. *Clinical Medicine & Engineering* 18, 245–246.

Geng, C. M. (2014). Clinical Effect of Qianlie Tongyu Capsules Combined with Tamsulosin Hydrochloride Sustained Release Capsules in Treatment of Benign Prostatic Hyperplasia. *Tianjin Pharmacy* 26, 37–38.

Gong, Y. C. (2020). Observation of the Efficacy of Xialiqi Capsules Combined with Tamsulosin Sustained-Release Capsule in the Treatment of Benign Prostatic Hyperplasia. *Journal of Practical Traditional Chinese Medicine* 36, 482–483.

Gu, D. L. (2021). Clinical study on Wenglitong Capsules combined with tamsulosin in treatment of benign prostatic hyperplasia. *Drugs & Clinic* 36, 577–581.

Guo, T. B., Yu, J. Y., Zhang, W. X., Yu, H., Xing, S. Q., Cui, Z. H., et al. (2021). Clinical observation of Xialiqi capsule combined with tamsulosin sustained release capsule in the treatment of benign prostatic hyperplasia. *Yiyao Qianyan* 11, 58–59.

Hao, C., Zhao, Z. L., and LI, Y. Z. (2009). Clinical effectiveness of combination therapy with Qianlieshutong capsule and doxazosin for benign prostatic hyperplasia. *Journal of Hunan University of Chinese Medicine* 29, 37–38.

Hizli, F., and Uygur, M. C. (2007). A prospective study of the efficacy of Serenoa repens, tamsulosin, and Serenoa repens plus tamsulosin treatment for patients with benign prostate hyperplasia. *International urology and nephrology* 39, 879–86. doi: 10.1007/s11255-006-9106-5.

Hu, J. S. (2019). Research and analysis of the clinical effect of Qianlie Shutong capsules combined with finasteride in the treatment of benign prostatic hyperplasia. *China Health Care & Nutrition* 29, 250.

Ji, D. L., Xue, C., Li, T., and Liu, P. (2018). Observation of the Efficacy of Longbishu Capsules Combined with epristeride in the Treatment of Benign Prostatic Hyperplasia. *Guizhou Medical Journal* 42, 989–990.

Ji, H. L. (2018). Clinical Efficacy Observation of Tamsulosin Combined with Longbishu Capsules in the Treatment of Benign Prostatic Hyperplasia. *Guide of China Medicine* 16, 125. doi: 10.15912/j.cnki.gocm.2018.08.108.

Jiang, F., and Wu, P. F. (2019). Effect of doxazosin mesylate combined with Wenglitong on benign prostatic hyperplasia and its effect on matrix metalloproteinase-9 and S100 calcium-binding protein P levels. *Hainan Medical Journal* 30, 562–565.

Kong, L. J., Wang, H. M., and Zhao, J. L. (2019). Effect of Qianlie Shutong Capsule combined with tamsulosin on urethral function in patients with benign prostatic hyperplasia. *Hainan Medical Journal* 30, 2373–2375.

Kong, X. H., Jing, X., and Dong, J. J. (2020). Clinical observation of Pulean Tablets combined with napidil in treatment of prostatic hyperplasia. *Drugs & Clinic* 35, 765–768.

Li, B. L. (2019a). Clinical Observation of Qianlie Shutong Capsules Combined with Finasteride in Treatment of Benign Prostatic Hyperplasia. *Drug Evaluation* 16, 19–20.

Li, H., Mi, Q. H., Gao, Y. J., and JIN, J. (2015). Effect of QianlieShutong capsule combined with tamsulosin on serum testosterone and estrogen in patients with benign prostatic hyperplasia and its efficacy. *Chinese Journal of Biochemical and Pharmaceuticals* 35, 89–91.

Li, H. Q., Sun, Y. F., and Liu, S. M. (2022). Clinical effects of Lingze tablets combined with tamsulosin hydrochloride in the treatment of benign prostatic hyperplasia. *Chinese Journal of Andrology* 36, 85–89.

Li, W., Feng, Y., and Wang, F. L. (2017). Clinical observation of Qianlie Beixi Capsules combined with Prostat Tablets in treatment of prostatic hyperplasia. *Drugs & Clinic* 32, 1955–1958.

Li, W. M. (2017). Observation on the Therapeutic Effect of Qianlie Shutong Capsules Combined with Finasteride in the Treatment of Benign Prostatic Hyperplasia. *Nei Mongol Journal of Traditional Chinese Medicine* 36, 76–77.

Li, W. Q., and Jin, F. L. (2020). Clinical Observation of Qianlie Shutong Capsule Combined with Finasteride in the Treatment of Benign Prostatic Hyperplasia in Middle-Aged and the Elderly. *Journal of Community Medicine* 18, 1333–1335. doi: 10.19790/j.cnki.JCM.2020.19.08.

Li, Z. H. (2019b). Clinical Study on Prostate Soothing Capsule Combined with Tamsulosin in Treatment of Benign Prostatic Hyperplasia. *Smart Healthcare* 5, 112–113. doi: 10.19335/j.cnki.2096-1219.2019.09.054.

Li, Z., Wang, Y., Wang, W. H., and Zhang, Y. H. (2009). Evaluation of clinical effect of ZeGuiLongShuang capsules combined with naftopidil on benign prostatic hyperplasia. *Journal of Jilin University (Medicine Edition)* 35, 936–939. doi: 10.13481/j.1671-587x.2009.05.041.

Li, Z. S. (2012). Treatment of 67 Cases of Benign Prostate Hyperplasia with the Combination of Pulean Tablets and Tamsulosin. *China Pharmaceuticals* 21, 69–70.

LIang, J. M., Zhao, G. Y., Luo, J. L., Huang, Y. H., Du, W. W., and Shi, Y. Q. (2018). Clinical study of prostaglandin capsule combined with finasteride in the treatment of benign prostatic hyperplasia. *Chinese Journal of Human Sexuality* 27, 13–15.

Liao, Q. X., Hou, B. N., Luo, X. G., and Liu, H. L. (2016). Clinical Observation on the Therapeutic Effect of Jingui Shenqi Pill on Elderly Patients with Benign Prostatic Hyperplasia. *Pharmacology and Clinics of Chinese Materia Medica* 32, 96–98. doi: 10.13412/j.cnki.zyyl.2016.05.025.

Liu, B. X., and Chen, G. H. (2013). Treatment of 17 Cases of Benign Prostatic Hyperplasia Complicated with Detrusor Instability with Jingui Shenqi Pill. *Chinese Journal of Surgery of Integrated Traditional and Western Medicine* 19, 179–181.

Liu, C. G., Shi, L., Duan, M., Xia, F. F., and Chen, X. P. (2010). Clinical Observation on the Combination of Qianlie Tongyu Capsule and Finasteride in the Treatment of Benign Prostatic Hyperplasia. *Practical Clinical Journal of Integrated Traditional Chinese and Western Medicine* 10, 39–40.

Liu, J. T., Zhang, C. L., and Yin, Y. H. (2009). Evaluation of the Efficacy of ZeGuiLongShuang Capsules Combined with α Receptor Blockers in the Treatment of Benign Prostatic Hyperplasia. *Acta Academiae Medicinae Weifang* 31, 401.

Lu, H. Z., Li, X. Y., and Liu, J. S. (2020). Clinical Study on Longbishu Capsules Combined with Doxazosin for Benign Prostatic Hyperplasia. *NEW CHINESE MEDICINE* 52, 48–50. doi: 10.13457/j.cnki.jncm.2020.21.015.

Luo, J. G. (2009). Prostat Combined with Longbishu Capsules for the Treatment of Benign Prostatic Hyperplasia. *Zhejiang Journal of Integrated Traditional Chinese and Western Medicine* 19, 105.

Luo, Q. Y., and Feng, P. (2020). Clinical Efficacy and Safety Evaluation of Xia Li Qi Capsule Combined with Tamsulosin Hydrochloride Sustained-Release Capsules in the Treatment of Benign Prostatic Hyperplasia. *Modern Journal of Integrated Traditional Chinese and Western Medicine* 29, 1311–1314.

Ma, Z. F., Wang, D. W., Liu, C., Yang, X. F., and Liang, X. Z. (2009). Observation of the Clinical Efficacy of Qianlie Shutong Capsules Combined with α Receptor Blocker in the Treatment of Benign Prostatic Hyperplasia. *Modern Journal of Integrated Traditional Chinese and Western Medicine* 18, 4107–4108.

Mai, H. L., Lan, P. G., and Yue, Z. M. (2023). Analysis of the Therapeutic Effect of Qianlie Shutong Capsules on Stasis Type Benign Prostatic Hyperplasia. *The Medical Forum* 27, 102–104. doi: 10.19435/j.1672-1721.2023.13.032.

Man, Y. P., He, Z. Y., and Li, G. H. (2017). Clinical observation of Qianlie Shutong Capsules combined with Epristeride Tablets in treatment of benign prostatic hyperplasia. *Drugs & Clinic* 32, 1093–1096.

Meng, F. (2017). Effect of Long Bi Shu capsule combined with finasteride in the treatment of benign prostatic hyperplasia. *Chinese Community Doctors* 33, 81+83.

Niu, M., Chen, T. F., Chen, S. J., Xia, H., Zhao, Q., Chen, L. J., et al. (2014). Observation on the Therapeutic Effect of Longbishu Capsules Combined with Epristeride Tablets in the Treatment of Benign Prostatic Hyperplasia (60 Cases). *China Practical Medicine* 9, 140–141. doi: 10.14163/j.cnki.11-5547/r.2014.28.307.

Peng, C. H. (2013). Clinical study on the combined treatment of traditional Chinese and Western medicine for benign prostatic hyperplasia of kidney deficiency and blood stasis type.

Qi, Y. J., and Wang, Y. M. (2011). Observation of the Efficacy of Qianlie Beixi Capsules Combined with Finasteride in the Treatment of Benign Prostatic Hyperplasia. *Zhejiang Journal of Integrated Traditional Chinese and Western Medicine* 21, 630–631.

Ryu, Y. W., Lim, S. W., Kim, J. H., Ahn, S. H., and Choi, J. D. (2015). Comparison of tamsulosin plus serenoa repens with tamsulosin in the treatment of benign prostatic hyperplasia in Korean men: 1-year randomized open label study. *Urologia internationalis* 94, 187–93. doi: 10.1159/000366521.

Shi, B., Chen, K., Zhang, Z. M., Chen, F., and Yang, X. S. (2016). Clinical observation of Qianlie Shutong Capsules combined with finasteride in treatment of benign prostatic hyperplasia. *Drugs & Clinic* 31, 500–503.

Song, C. S., Zhao, J. Y., Guo, J., Chang, D. G., Chen, L., Zhang, R., et al. (2016). Treatment of Benign Prostatic Hyperplasia by Longbishu Capsule Combined Doxazosin Mesylate Tablets. *Chinese Journal of Integrated Traditional and Western Medicine* 36, 1465–1469.

Su, H. Y. (2015). Clinical efficacy of Qianlie Tongyu combined with Tamsulosin in the treatment of benign prostatic hyperplasia. *Chinese Journal of Urban and Rural Enterprise Hygiene* 30, 132–133. doi: 10.16286/j.1003-5052.2015.04.057.

Su, M. (2012). Observation on the Therapeutic Effect of Terazosin Combined with Pulean in the Treatment of Benign Prostatic Hyperplasia. *Strait Pharmaceutical Journal* 24, 174–175.

Sun, J., Li, M., Zhang, J., Qu, W., and Hu, J. N. (2019). Clinical Observation on Huange Capsules Combined with Finasteride in the Treatment of Senile Benign Prostatic Hyperplasia. *China Pharmaceuticals* 28, 87–89. doi: 10.3969/j.issn.1006-4931.2019.11.027.

Tao, X. F., Deng, G., and Jia, H. J. (2012). Observation on the Treatment of Benign Prostate Hyperplasia with Longbishu Capsules Combined with Finasteride. *Chinese Journal of Aesthetic Medicine* 21, 234. doi: 10.15909/j.cnki.cn61-1347/r.2012.10.287.

Tian, Z. Y., Feng, W., Zhu, Y. Q., Sun, X. L., and Xu, X. (2018). Curative Efficacy of Qianlie Shutong Capsules Combined with Finasteride in Treatment of Benign Prostatic Hyperplasia. *Medical Journal of West China* 30, 224–227. doi: 10.3969/j.issn.1672-3511.2018.02.017.

Tong, P., Song, S. H., Yang, J., and Zeng, S. S. (2020). The Effect of Qianlie Shutong Capsules on Clinical Symptoms of Benign Prostatic Hyperplasia. *Journal of North Pharmacy* 17(01), 84–85.

Wan, T. (2017). The Therapeutic Effect of Qianlie Shutong Capsules Combined with Tamsulosin in the Treatment of Benign Prostatic Hyperplasia and the Impact on Serum Testosterone and Estradiol Levels. *Modern Journal of Integrated Traditional Chinese and Western Medicine* 26, 2370–2372.

Wang, C. L., Wang, Y. G., Zhang, J., and Kong, T. (2022). Clinical Study on Lingze Tablets Combined with Tamsulosin Hydrochloride for Benign Prostatic Hyperplasia of Kidney Deficiency and Blood Stasis Type. *NEW CHINESE MEDICINE* 54, 80–83. doi: 10.13457/j.cnki.jncm.2022.04.022.

Wang, C. X., GONG, X. Y., Zhang, X. Y., and Liu, Y. W. (2017). Efficacy Observation of Qianliexin Combined with Tamsulosin Hydrochloride and Finasteride in the Treatment of Elderly Benign Prostatic Hyperplasia. *China Pharmacy* 28, 1108–1111.

Wang, G. R., and Yu, G. F. (2018). Efficacy of Qianlie Shutong Capsules in Adjuvant Treatment of Benign Prostatic Hyperplasia and Its Effect on Serum Testosterone and Estradiol Levels. *Journal of Shandong First Medical University & Shandong Academy of Medical Sciences* 39, 570–571.

Wang, J. (2014). Clinical Efficacy Analysis of Terazosin Combined with Qianlie Shutong Capsules in the Treatment of Benign Prostatic Hyperplasia. *Henan Journal of Surgery* 20, 64–65. doi: 10.16193/j.cnki.hnwk.2014.01.086.

Wang, S. Q., Huang, W., Huang, H. H., Peng, Y. P., and Zhou, J. S. (2020). The Effect of Longbishu Capsules Combined with Finasteride on Residual Urine Output in Elderly Patients with Benign Prostatic Hyperplasia. *Chinese Journal of Gerontology* 40, 1675–1677.

Wang, Y. (2006). Clinical Observation on the Treatment of Benign Prostate Hyperplasia with Liuwei Dihuang Pills Combined with Epristeride. *Journal of Jinzhou Medical University*, 64–65.

Wei, L. Y., Wei, Z. X., and Huang, S. H. (2016). Treatment of Hyperplasia of Prostate with Combination of Traditional Chinese and Western Medicine. *Clinical Medicine* 36, 123–124.

Wu, H. J., and Wu, J. (2019). Effects of Tamsulosin Hydrochloride Sustained-release Capsule Combined with Qianliexin Capsule on the Efficacy and Sexual Function of Patients with Benign Prostatic Hyperplasia. *Drug Evaluation* 16, 66–67.

Wu, X. H. (2019). Exploring the Clinical Efficacy of Qianlie Shutong Capsules Combined with Finasteride in the Treatment of Benign Prostatic Hyperplasia. *Chinese Medical Journal of Metallurgical Industry* 36, 156–157. doi: 10.13586/j.cnki.yjyx1984.2019.02.023.

Xia, H. Q. (2013). Clinical efficacy of saw palmetto extract combined with tamsulosin for the treatment of benign prostatic hyperplasia. *Chongqing Medicine* 42, 2489–2490.

Xiang, Y., and Xiao, D. (2012). Clinical Observation of Terazosin Combined with Zegui Huangshuang in the Treatment of Benign Prostatic Hyperplasia. *Chinese Community Doctors* 14, 219–220.

Xu, M. K., Hao, Y. L., and LIU, H. (2020). Curative effect of Qianliexin combined with doxazosin tablets on benign prostatic hyperplasia in the elderly. *Hainan Medical Journal* 31, 851–854.

Xu, Z. C., Zhang, M. J., Su, M. Y., and Qiu, Q. (2021). Clinical controlled research of Jingui Shenqi Pill in the treatment of patients with benign prostatic hyperplasia of Yang deficiency. *China Medicine and Pharmacy* 11, 47-49+79.

Xuan, S. Q., Wu, D. F., Wei, H. S., and Yang, Y. (2012). Observation on the Therapeutic Effect of Qianlie Shutong Capsules Combined with Tamsulosin in the Treatment of Benign Prostatic Hyperplasia. *Zhejiang Journal of Integrated Traditional Chinese and Western Medicine* 22, 708–709.

Xue, P., Ren, B., Zhang, X. Y., and Zhang, J. W. (2017). The efficacy and safety of Longbishu combined with Tamsulosin hydrochloride sustained-release capsules in the treatment of elderly prostate hyperplasia. *Chinese Journal of Human Sexuality* 26, 30–32.

Yang, Z., and Wang, Y. (2019). Clinical study on Xialiqi Capsules combined with finasteride in treatment of benign prostatic hyperplasia. *Drugs & Clinic* 34, 3071–3075.

Ye, Y. S., and Lu, W. (1994). Clinical Observation on the Treatment of Benign Prostatic Hyperplasia with Prazosin and Qianliekang Tablet. *Chinese Journal of Medicine*, 51–52.

Yin, Z. K., Wang, Y. Y., and Fan, X. M. (2018). Clinical Efficacy of Qianlie Shutong Capsules Combined with Finasteride in the Treatment of Benign Prostatic Hyperplasia. *Chinese Journal of Clinical Rational Drug Use* 11, 91–92. doi: 10.15887/j.cnki.13-1389/r.2018.27.046.

Yu, H. C., Zhong, W. Y., and Bao, P. (2016). Randomized controlled study of Qianlie Shutong combined with Tamsulosin in the treatment of prostatic hyperplasia. *China Practical Medicine* 11, 1-3. doi: 10.14163/j.cnki.11-5547/r.2016.24.001.

Yu, S. C., and He, H. B. (2017). The effect of sustained-release tablets of Tamoxifen hydrochloride combined with Guizhi Fuling pills on the urodynamics of benign prostatic hyperplasia. *Modern Journal of Integrated Traditional Chinese and Western Medicine* 26, 631–633.

Yuan, K. N., She, M. H., and Han, F. Q. (2021). Observation on the Efficacy of Huange Capsule Combined with Finasteride in the Treatment of Prostatic Hyperplasia in Middle-Aged and Elderly People. *China Pharmaceuticals*, 28(11),87-89.

Yuan, M. D. (2017). Effect of Longbishu Capsule on Serum IL-10 and TNF-α in the Treatment of Benign Prostatic Hyperplasia. *Journal of Liaoning University of Traditional Chinese Medicine* 19, 147–150. doi: 10.13194/j.issn.1673-842x.2017.08.042.

Zhang, D. X., Huang, C., Wang, S. Y., and Zhu, H. J. (2022a). Clinical Efficacy of Longbishu Tablets Combined with Tamsulosin Hydrochloride Sustained-Release Capsules in the Treatment of Benign Prostatic Hyperplasia. *Chinese Journal of Clinical Rational Drug Use* 15, 111–113. doi: 10.15887/j.cnki.13-1389/r.2022.15.035.

Zhang, F. (2017). The Therapeutic Effect of Qianlie Shutong Capsules Combined with Tamsulosin in the Treatment of Benign Prostatic Hyperplasia and Its Impact on Serum Testosterone and Estradiol Levels. *Modern Journal of Integrated Traditional Chinese and Western Medicine* 26, 274–276.

Zhang, J. M. (2020). Analysis of the Therapeutic Effect of Qianlie Shutong Capsules Combined with Finasteride in the Treatment of Benign Prostatic Hyperplasia. *Cardiovascular Disease Electronic Journal of Integrated Traditional Chinese and Western Medicine* 8, 56+59. doi: 10.16282/j.cnki.cn11-9336/r.2020.25.042.

Zhang, T. B., Deng, Y. J., Zheng, T., Nan, Y. H., Sun, Y. Y., Lu, K. L., et al. (2020). Clinical study on Qianqianshu Pills combined with doxazosin in treatment of prostatic hyperplasia. *Drugs & Clinic* 35, 243–246.

Zhang, X., Liang, T. S., Wang, J., Wu, G., and Luo, X. D. (2022b). Clinical Trial of Qianlie Shutong Capsules Combined with Tamsulosin Hydrochloride Sustained-Release Capsule and Finasteride in the Treatment of Patients with Benign Prostatic Hyperplasia. *The Chinese Journal of Clinical Pharmacology* 38, 2273–2277. doi: 10.13699/j.cnki.1001-6821.2022.19.006.

Zhang, X. S., and Cheng, H. S. (2012). Wenglitong Capsule and Western Medicine in Treatment of Benign Prostatic Hyperplasia. *Chinese Journal of Experimental Traditional Medical Formulae* 18, 325–327. doi: 10.13422/j.cnki.syfjx.2012.23.100.

Zhang, Y. J. (2016). Efficacy Observation of 46 Cases of Benign Prostatic Hyperplasia Treated with the Combined Medication of Longbishu Capsules and Tamsulosin. *World Journal of Integrated Traditional and Western Medicine* 11, 101–103. doi: 10.13935/j.cnki.sjzx.160130.

Zhang, Y. L. (2012). Effect of Guizhi Fuling Capsules Combined with Tamsulosin on Clinical Symptoms and Quality of Life in Elderly Patients with Prostatic Hyperplasia. *Chinese Journal of Gerontology* 32, 4800–4802. doi: 10.3969/j.issn.1005-9202.2012.21.103.

Zhang, Y., and Liu, B. X. (2015). The Curative Effect of Tamsulosin Combined with Jinguishenqi Pill in Treating Patients with Benign Prostatic Hyperplasia Coexisting with Erectile Dysfunction. *Chinese Journal of Human Sexuality* 24, 3–5.

Zhang, Z. L., Wang, G. P., and Wang, H. M. (2009). 60 cases of benign prostatic hyperplasia treated with traditional Chinese medicine combined with Finasteride. *Shaanxi Journal of Traditional Chinese Medicine* 30, 1489–1490.

Zhao, H., Han, S., Du, L. Y., Zhang, Y. S., He, L., and Wang, S. L. (2019). Effects of Guizhi Fuling Capsules on Clinical Symptoms, Sleep Quality and Life Quality in Treatment of Patients with Prostatic Hyperplasia. *Medical & Pharmaceutical Journal of Chinese People’s Liberation Army* 31, 92–96. doi: 10.3969/j.issn.2095-140X.2019.07.022.

Zhao, M. J., Sha, K. F., and Wang, Y. (2023). Clinical observation of Lingze Tablets combined with epristeride in treatment of benign prostatic hyperplasia. *Drugs & Clinic* 38, 1203–1207.

Zhao, X. S., Du, J., Chen, H. L., and Wang, X. L. (2020). A Clinical Study of Qianliexin Capsule Combined with Terazosin in the Treatment of Prostatic Hyperplasia. *Drugs & Clinic* 35, 2216–2219.

Zhi, Q. M., Huang, Z. M., Zhu, H. B., Li, P. Y., and Guo, W. Q. (2020). Clinical Observation of Jingui Shenqi Pill Combined with Finasteride in the Treatment of Prostatic Hyperplasia (Shen Yang Deficiency Syndrome). *Inner Mongolia Journal of Traditional Chinese Medicine* 39, 84–85. doi: 10.16040/j.cnki.cn15-1101.2020.04.051.

Zhou, P. G. (2021). Efficacy and Safety Analysis of Saw Palmetto Extract Combined with α-Blockers in the Treatment of Benign Prostatic Hyperplasia. *Chinese Journal of Clinical Rational Drug Use* 14, 92–94. doi: 10.15887/j.cnki.13-1389/r.2021.27.032.

Zhou, Q., Li, H. X., Wang, L., Liu, R. B., and Zhao, G. H. (2018). Clinical effects of Longbishu Capsules in the adjuvant treatment for benign prostatic hyperplasia patients due to Kidney Deficiency and Blood Stasis Pattern. *Chinese Traditional Patent Medicine* 40, 2399–2403.

Zhou, S. M., Guo, Q. L., and Qin, F. (2023). Clinical Study on Qianlie Shutong Capsules Combined with Tamsulosin Hydrochloride Sustained-Release Capsules for Patients After Surgery for Prostatic Hyperplasia. *NEW CHINESE MEDICINE* 55, 95–99. doi: 10.13457/j.cnki.jncm.2023.02.023.

Zhou, X. P., Yang, Y. G., Xu, H. P., Wan, W. Y., Liu, H., Liu, J. M., et al. (2014). Clinical effect observation of tamsulosin combined with sabal fruit extract in soft capsule in the treatment of benign prostatic hyperplasia. *China Modern Medicine* 21, 66-67.

Zhu, H. G., Gong, R. J., Han, M., Qiu, J., and Hu, B. Y. (2011). Combination of Qianlie Shutong Capsules and Tamoxifen Hydrochloride Sustained Release Capsules in the Treatment of 28 Cases of Small Volume Benign Prostate Hyperplasia. *Herald of Medicine* 30, 1176–1178.

Zhu, J. H. (2023). Lingze Pill Combined with Tamsulosin Hydrochloride Sustained Release Capsules in the Treatment of Benign Prostatic Hyperplasia. *Chinese Medicine Modern Distance Education of China* 21, 132–135.

# File S4: Details of the included poly-herbal TCM formulations.

## Plant material and initial processing of poly-herbal TCM formulations.

| **Poly-herbal TCM formulations** | **Description of the botanical drug and taxonomic authentication** | **Description of the extract and extraction process** | **Indication, adverse reactions and contraindications** | **Source** | **Quality control reported. (Y/N)** |
| --- | --- | --- | --- | --- | --- |
| Guizhi Fuling Capsule | *Cinnamomum cassia* Presl [Lauraceae], *Poria cocos* (Schw.) Wolf [Polyporaceae], *Paeonia suffruticosa* Andrews [Paeoniaceae], *Prunus persica* (L.) Batsch [Rosaceae], *Paeonia lactiflora* Pall. [Paeoniaceae] | Grind Poria cocos into fine powder. Distill the bark of peony bark with water vapor, collect the distillate, and separate the volatile components. The residue, Cinnamomum cassia, Paeonia lactiflora, Prunus persica, and the remaining Poria cocos are secondary extracted with 90% ethanol. The extracts are combined, and recover the ethanol until there is no alcoholic odor, followed by vacuum concentration to an appropriate volume. The residue is then boiled in water a second time, filtered, and concentrated under vacuum to a suitable volume. The two types of concentrated liquid, along with the Poria cocos powder, are mixed thoroughly, dried, and ground. An appropriate amount of dextrin is added to form granules. The volatile components from the Paeonia suffruticosa bark are incorporated, mixed well, and encapsulated to obtain the final product. | Indication: Promoting blood circulation, resolving stasis, and relieving mass.  Adverse reactions: Occasional discomfort or pain in the stomach, which can disappear after stopping the medication.  Contraindications: Not suitable for pregnant women. | Jiangsu Kanion Pharmaceutical Co., Ltd. | Y-National Pharmaceutical Standard: Z10950005 |
| Huange Capsule | *Astragalus membranaceus* (Fisch.) Bunge [Fabaceae], *Prunus persica* (L.) Batsch [Rosaceae], *Curcuma zedoaria* (Christm.) Roscoe [Zingiberaceae], *Rheum palmatum* L. [Polygonaceae], *Smilax glabra* Roxb. [Smilacaceae], *Coix lacryma-jobi var. ma-yuen* (Rom.Caill.) Stapf [Poaceae], *Leonurus japonicus* Houtt. [Lamiaceae], *Prunella vulgaris* L. [Lamiaceae], *Cinnamomum cassia* Presl [Lauraceae], *Menispermum dauricum* DC. [Menispermaceae], *Platycodon grandiflorus* (Jacq.) A.DC. [Campanulaceae], *Cyathula officinalis* Kuan [Amaranthaceae] | Grind Cinnamomum cassia, Rheum palmatum, Coix lacryma-jobi, and Prunus persica into a fine powder. Extract essential oils from Curcuma zedoaria slices. Perform aqueous extraction followed by alcohol precipitation with Astragalus membranaceus. Combine the aqueous filtrates of other herbal medicines and the residue from the Curcuma zedoaria extraction with the Astragalus ethanol solution, and concentrate to form a dry extract. This extract is then ground, sieved, and mixed with the essential oil from Curcuma zedoaria and the fine powders of Cinnamon and other medicines. This mixture is subsequently combined with pharmaceutical excipients to manufacture capsules. | Indication: Tonifying qi, promoting blood circulation, and clearing damp-heat.  Adverse reactions: Some patients may experience nausea, abdominal pain, diarrhea, dry stools, abdominal bloating, stomach discomfort, chest discomfort, palpitations, painful urination, etc.  Contraindications: Individuals with severe gastritis, stomach, and duodenal ulcers. | Zhejiang Conba Pharmaceutical Co.,Ltd. | Y-National Food and Drug Administration National Drug Standard: YBZ00202011; National Pharmaceutical Standard: Z20110006 |
| Jingui Shenqi Pill | *Rehmannia glutinosa* (Gaertn.) DC. [Orobanchaceae], *Dioscorea oppositifolia* L. [Dioscoreaceae], *Cornus officinalis* Siebold & Zucc. [Cornaceae], *Poria cocos* (Schw.) Wolf [Polyporaceae], *Paeonia suffruticosa* Andrews [Paeoniaceae], *Alisma orientale* (Sam.) Juz. [Alismataceae], *Cinnamomum cassia* Presl [Lauraceae], *Aconitum carmichaelii* Debeaux [Ranunculaceae], *Achyranthes bidentata* Blume [Amaranthaceae], *Plantago asiatica* L. [Plantaginaceae] | Grind the herbal medicines into a fine powder, sift, and mix evenly. For every 100g of powder, add 110 to 130g of refined honey to form large honey pills. | Indication: Warming and supplementing kidney yang, promoting qi circulation, and eliminating water retention.  Adverse reactions: Not reported.  Contraindications: Not suitable for pregnant women. | Tong Ren Tang Technologies Co.Ltd. | Y-National Food and Drug Administration National Drug Standard: WS3-B-3892-98-2011; National Pharmaceutical Standard: Z11020054 |
| Longbishu Capsule | *Cullen corylifolium* (L.) Medik. [Fabaceae], *Leonurus japonicus* Houtt. [Lamiaceae], *Lysimachia christinae* Hance [Primulaceae], *Ophiopogon japonicus* (L.f.) Ker Gawl. [Asparagaceae], *Succinum*, *Cremastra appendiculata* (D.Don) Makino [Orchidaceae] | Grind amber into a fine powder. Boil the remaining herbal medicines in water twice, filter, and combine the filtrates. Then, concentrate under reduced pressure to form a clear paste. Proceed with spray drying, and mix the resulting powder evenly with the amber powder and an appropriate amount of starch. Finally, encapsulate this mixture to obtain the finished product. | Indication: Tonifying kidney, promoting blood circulation, clearing heat, and promoting urination.  Adverse reactions: Nausea, vomiting, abdominal pain, bloating, diarrhea, stomach discomfort, thirst etc.  Contraindications: Pregnant women, individuals allergic to any components of the product, or those with abnormal liver function. | Shijiazhuang Kedi Pharmaceutical Co., Ltd. | Y-National Pharmaceutical Standard: Z10960007 |
| Liuwei Dihuang Pill | *Rehmannia glutinosa* (Gaertn.) DC. [Orobanchaceae], *Cornus officinalis* Siebold & Zucc. [Cornaceae], *Paeonia suffruticosa* Andrews [Paeoniaceae], *Dioscorea oppositifolia* L. [Dioscoreaceae], *Poria cocos* (Schw.) Wolf [Polyporaceae], *Alisma orientale* (Sam.) Juz. [Alismataceae]. Additional ingredient: Talcum Powder | Grind the herbal medicines into a fine powder, sift, and mix evenly. Utilize ethanol to coat the pills, dry them, and form water pills, or for every 100g of powder, add 80g of refined honey to produce honey pills. | Indication: Nourishing yin and tonifying kidney. Adverse reactions: Diarrhea, abdominal pain, bloating, nausea, vomiting, loss of appetite, constipation, rash, headache, allergies, etc.  Contraindications: Individuals allergic to any components of the product. | Nanjing Tongrentang Pharmaceutical Co., Ltd | Y-National Pharmaceutical Standard: Z32020636 |
| Lingze Tablet | *Xylaria nigripes* (Kl.) Sacc. (Xylariaceae), *Curcuma zedoaria* (Christm.) Roscoe [Zingiberaceae], *Fritillaria thunbergii* Miq. [Liliaceae], *Alisma orientale* (Sam.) Juz. [Alismataceae] | Essential oil is extracted from Curcuma zedoaria via steam distillation for 12 hours. The volatile oil is then added to a saturated aqueous solution of beta-cyclodextrin, stirred for 3 hours, and left to stand overnight. The lower layer of the precipitated inclusion compound is filtered, collecting the inclusion compound. Fritillaria thunbergii and Alisma plantago-aquatica are added to water, combined with the post-extraction residue and liquid of the volatile oil, and boiled three times, each for 2 hours. The combined decoction is filtered, and the filtrate is concentrated under reduced pressure to a clear paste. Ethanol is added to reach an alcohol content of 70%, left to stand overnight, and the supernatant is concentrated under vacuum to a clear paste. After spray drying, Xylaria nigripes powder and the previously mentioned inclusion compound are added, along with an appropriate amount of calcium hydrogen phosphate, and mixed evenly. Using a 2% solution of hydroxypropyl methylcellulose as a binder, the mixture is granulated, dried, magnesium stearate is added, and then compressed into tablets. | Indication: Tonifying kidney, promoting blood circulation, dispersing masses, and promoting diuresis.  Adverse reactions: Some patients may experience dry mouth, hiccups, nausea, stomach bloating, heartburn, stomach pain, diarrhea, etc.  Contraindications: Not reported. | Zhejiang Jolly Pharmaceutical Co.,Ltd. | Y-National Pharmaceutical Standard: Z20110050 |
| Qianliekang Tablet (Pulean Tablet) | *Brassica rapa* L. [Brassicaceae] | Collect rapeseed pollen, grind it into a fine powder, sift, and add an appropriate amount of auxiliary materials such as sodium carboxymethyl starch, sodium carboxymethyl cellulose, and silicon dioxide. Mix evenly, granulate, dry, form into tablets, and coat with a thin film to obtain the finished product. | Indication: Tonifying kidney and securing the foundation.  Adverse reactions: A small number of patients may experience mild loose stools.  Contraindications: Individuals allergic to any components of the product. | Zhejiang Conba Pharmaceutical Co.,Ltd. | Y-National Pharmaceutical Standard: Z33020303 |
| Qianlie Beixi Capsule | *Adiantum capillus-veneris* L.[Pteridaceae], *Gryllotalpa orientalis* Burmeister [Gryllotalpidae], *Vaccaria segetalis* (Neck.) Garcke, *Gleditsia sinensis* Lam. [Fabaceae], *Erinaceus europaeus* L. (Scalded) [Erinaceidae] | Grind Gryllotalpa orientalis, Vaccaria segetalis, and hedgehog skin, sift, and set aside. Extract the Adiantum capillus-veneris and Gleditsia sinensis by adding water and refluxing for 2 hours. Concentrate the extract to a clear paste with a relative density of 1.20 to 1.25 (at 80°C). Mix in the previously ground medicine powder, vacuum dry, grind, sift, and encapsulate to obtain the final product. | Indication: Clearing damp-heat, promoting blood circulation, resolving stasis, promoting diuresis, and relieving strangury.  Adverse reactions: Not reported.  Contraindications: Not suitable for pregnant women. | Guizhou Taihe Pharmaceutical Co.,Ltd. | Y-National Drug Standard: WS-10027(ZD-0027)-2002-2012Z; National Pharmaceutical Standard: Z20025028 |
| Qianlieshu Pill | *Rehmannia glutinosa* (Gaertn.) DC. [Orobanchaceae], *Coix lacryma-jobi* L. [Poaceae], *Benincasa hispida* (Thunb.) Cogn. [Cucurbitaceae], *Cornus officinalis* Siebold & Zucc. [Cornaceae], *Dioscorea oppositifolia* L. [Dioscoreaceae], *Paeonia suffruticosa* Andrews [Paeoniaceae], *Atractylodes lancea* (Thunb.) DC. [Asteraceae], *Prunus persica* (L.) Batsch [Rosaceae], *Alisma orientale* (Sam.) Juz. [Alismataceae], *Poria cocos* (Schw.) Wolf [Polyporaceae], *Cinnamomum cassia* Presl [Lauraceae], *Aconitum carmichaelii* Debeaux [Ranunculaceae], *Allium tuberosum* Rottler ex Spreng. [Amaryllidaceae], *Epimedium brevicornu* Maxim. [Berberidaceae], *Glycyrrhiza uralensis* Fisch. [Fabaceae] | Grind the herbal medicines into a fine powder, sift, and mix evenly. For every 100g of powder, add 110 to 130g of refined honey to produce honey pills. | Indication: Strengthening body resistance, stabilizing the foundation, and promoting kidney function for diuresis.  Adverse reactions: Not reported.  Contraindications: Not reported. | Weihai Rensheng Pharmaceutical Co., Ltd. | Y-National Food and Drug Administration National Drug Standard: YBZ01202015; National Pharmaceutical Standard: Z10910008 |
| Qianlie Shutong Capsule | *Phellodendron chinense* Schneid. [Rutaceae], *Paeonia lactiflora* Pall. [Paeoniaceae], *Angelica sinensis* (Oliv.) Diels [Apiaceae], *Ligusticum chuanxiong* Hort. [Apiaceae], *Smilax glabra* Roxb. [Smilacaceae], *Sparganium stoloniferum* Buch.-Ham., *Alisma orientale* (Sam.) Juz. [Alismataceae], *Portulaca oleracea* L. [Portulacaceae], *Verbena officinalis* L. [Verbenaceae], *Bupleurum chinense* DC. [Apiaceae], *Cyathula officinalis* Kuan [Amaranthaceae], *Glycyrrhiza uralensis* Fisch. [Fabaceae] | Take Phellodendron, Alisma orientale, Angelica sinensis, and Ligusticum chuanxiong, and extract them twice with 60% ethanol, each time for 1.5 hours, under reflux. Filter, and recover ethanol from the filtrate under reduced pressure, setting aside the resulting clear paste. Separately, take the other herbal medicines, boil twice with water, the first time for 1.5 hours and the second time for 1 hour. Filter, combine the filtrates, and concentrate under reduced pressure to a clear paste with a relative density of 1.08 (at 60°C). Combine this paste with the previously prepared paste, concentrate to a thick paste with a relative density of 1.32 to 1.35 (at 60°C), vacuum dry, grind the dry paste, mix with starch evenly to form granules, dry, and encapsulate to obtain the final product. | Indication: Clearing heat, promoting diuresis, resolving dampness, dispersing masses, and promoting blood circulation to resolve stasis.  Adverse reactions: Gastrointestinal issues such as nausea, vomiting, diarrhea, abdominal pain; Skin and subcutaneous tissue disorders such as rash, itching; nervous system disorders like dizziness, headache.  Contraindications: Individuals allergic to any components of the product. | Baoding Buchang Tianhao Pharmaceutical Co.,Ltd. | Y-National Food and Drug Administration National Drug Standard: WS-11328(ZD-1328)-2002-2012Z; National Pharmaceutical Standard: Z20027140 |
| Qianlie Tongyu Capsule | *Paeonia lactiflora* Pall. [Paeoniaceae], *Eupolyphaga sinensis* Walker, *Manis pentadactyla* Linnaeus [Manidae], *Prunus persica* (L.) Batsch [Rosaceae], *Pyrrosia lingua* (Thunb.) Farw. [Polypodiaceae], *Prunella vulgaris* L. [Lamiaceae], *Angelica dahurica* (Fisch. ex Hoffm.) Benth. & Hook.f. ex Franch. & Sav. [Apiaceae], *Astragalus membranaceus* (Fisch.) Bunge [Fabaceae], *Pyrola* *calliantha* Andres [Ericaceae], *Ostrea* *gigas* Thunberg [Ostreidae], *Tetrapanax* *papyriferus* (Hook.) K. Koch | Crush Ostrea gigas into a fine powder. Extract Paeonia lactiflora with 75% ethanol by reflux to obtain an extract. Immerse Eupolyphaga sinensis and Prunus persica in 75% ethanol to extract their essences. Boil the remaining herbal ingredients to produce an extract. Add an appropriate amount of starch to ensure a consistent granule weight. Mix the extracts with the raw herbal powder and starch evenly. To ensure the granules are uniformly loose, use ethanol as a wetting agent to produce wet granules. Dry at 80°C, crush to size, and fill into capsules. | Indication: Promoting blood circulation, resolving stasis, and clearing heat to promote diuresis.  Adverse reactions: Occasional upper abdominal discomfort or pain.  Contraindications: Not reported. | Zhuhai Xingguang Pharmaceutical Co., Ltd | Y-National Food and Drug Administration National Drug Standard: WS3-405(Z-56)-2003(Z); National Pharmaceutical Standard: Z19990060 |
| Qianliexin Capsule | *Prunus* *persica* (L.) Batsch [Rosaceae], *Commiphora* *myrrha* Engl. (C. molmol Engler), *Salvia* *miltiorrhiza* Bunge [Lamiaceae], *Paeonia* *lactiflora* Pall. [Paeoniaceae], *Carthamus* *tinctorius* L. [Asteraceae], *Lycopus* *lucidus* Turcz. [Lamiaceae], *Vaccaria* *segetalis* (Neck.) Garcke, *Gleditsia* *sinensis* Lam. [Fabaceae], *Patrinia* *scabiosaefolia*, *Taraxacum* *mongolicum* Hand-Mazz. [Asteraceae], *Melia* *toosendan* Siebold & Zucc. [Meliaceae], *Angelica* *dahurica* (Fisch. ex Hoffm.) Benth. & Hook.f. ex Franch. & Sav. (Processed) [Apiaceae], *Pyrrosia* *lingua* (Thunb.) Farw. [Polypodiaceae], *Lycium* *barbarum* L. [Solanaceae] | Commiphora myrrha, Gleditsia sinensis, and Angelica dahurica is ground into a fine powder. Boil the remaining ingredients twice with water. Filter and combine the filtrates, then concentrate into a thick paste. Incorporate the aforementioned fine powder, mix well, and dry below 80°C. Grind into a fine powder, mix evenly, and encapsulate to obtain final capsules. | Indication: Promoting blood circulation, resolving stasis, clearing heat, and promoting diuresis.  Adverse reactions: Not reported.  Contraindications: Not reported. | Shandong Hongjitang Pharmaceutical Group Co., Ltd. | Y-National Pharmaceutical Standard: Z10950010 |
| Saw Palmetto Extract | *Serenoa* *repens* (W.Bartram) Small [Arecaceae] | Heat polyethylene glycol to a specific temperature until completely melted, then add the extract of Saw Palmetto fruit, stirring until fully dissolved. Rapidly cool under stirring conditions, then leave to sit for 24 hours to form a brittle, easily breakable substance. Crush, sift, and obtain a Saw Palmetto fruit extract - polyethylene glycol solid dispersion inclusion compound. Thoroughly mix into vegetable oil to achieve a uniform and stable suspension. Add the suspension and capsule shell material to a softgel encapsulation machine, press, shape, and dry to obtain the final product. | Indication: Used in the early stage of benign prostatic hyperplasia, such as urinary frequency, urgency, and difficulty in urination.  Adverse reactions: Rare occurrences of stomach pain after taking the product.  Contraindications: Not reported. | TAD Pharma GmbH | Y-National Pharmaceutical Standard: Z20170002 |
| Wenglitong Capsule | *Coix* *lacryma-jobi* L. [Poaceae], *Fritillaria* *thunbergii* Miq. [Liliaceae], *Clematis* *armandii* Franch. [Ranunculaceae], *Gardenia* *jasminoides* J.Ellis [Rubiaceae], *Lonicera* *japonica* Thunb. [Caprifoliaceae], *Inula* *japonica* Thunb. [Asteraceae], *Lycopus* *lucidus* Turcz. [Lamiaceae], *Rheum* *palmatum* L. [Polygonaceae], Verdigris, *Glycyrrhiza uralensis* Fisch. [Fabaceae], *Astragalus* *membranaceus* (Fisch.) Bunge [Fabaceae] | The herbal medicines are decocted twice with water. After filtering, the filtrates are combined and concentrated under reduced pressure to form a paste. This paste is then spray-dried, mixed with starch to form granules, further dried and then encapsulated to obtain the product. | Indication: Clearing heat, promoting diuresis, dispersing masses, and eliminating stasis.  Adverse reactions: Not reported.  Contraindications: Not reported. | Jilin Huawei Pharmaceutical Co., Ltd. | Y-National Food and Drug Administration National Drug Standard: WS3-157(Z-13)-2002(Z)-2; National Pharmaceutical Standard: Z20043767 |
| Xialiqi Capsule | *Astragalus* *membranaceus* (Fisch.) Bunge [Fabaceae], *Ligustrum* *lucidum* W.T.Aiton [Oleaceae], Talcum Powder, *Prunella vulgaris* L. [Lamiaceae], *Litchi* *chinensis* Sonn. [Sapindaceae], *Succinum*, *Cinnamomum verum* J.Presl [Lauraceae], *Phellodendron* *amurense* Rupr. [Rutaceae] | The herbal medicines are decocted twice with water. After filtering, the filtrates are combined and concentrated under reduced pressure to form a paste. This paste is then spray-dried, mixed with starch to form granules, further dried and then encapsulated to obtain the product. | Indication: Strengthening the spleen, nourishing the kidney, promoting diuresis, and dispersing masses. Adverse reactions: Stomach discomfort.  Contraindications: Individuals allergic to any components of the product. | Shijiazhuang Yiling Pharmaceutical Co., Ltd. | Y-National Food and Drug Administration National Drug Standard: YBZ00022012 National Pharmaceutical Standard: Z20123085 |
| Zegui Longshuang Capsule | *Lycopus* *lucidus* Turcz. [Lamiaceae], *Gleditsia* *sinensis* Lam. [Fabaceae], *Cinnamomum* *verum* J.Presl [Lauraceae] | The herbal medicines are decocted twice with water. After filtering, the filtrates are combined and concentrated under reduced pressure to form a paste. This paste is then spray-dried, mixed with starch to form granules, further dried and then encapsulated to obtain the product. | Indication: Dispersing stasis, resolving masses, promoting qi transformation, and promoting water metabolism.  Adverse reactions: Some patients may experience nausea, stomach discomfort, stomach pain, poor appetite, and diarrhea.  Contraindications: Not reported. | Nanjing Chia-Tai Tianqing Pharmaceutical Co., Ltd. | Y-National Food and Drug Administration National Drug Standard: WS3-195(Z-044)-2003(Z); National Pharmaceutical Standard: Z20174006 |

## Analytical methods for the chemical profile of poly-herbal TCM formulations according to ConPhyMP.

| **Poly-herbal TCM formulations** | **Type of extract** | **Preferred/main methods and component identification results for extract characterisation/chemical analysis** | **Alternative methods and component identification results for extract characterisation/chemical analysis** | **Reference** |
| --- | --- | --- | --- | --- |
| Guizhi Fuling Capsule | A | (1) Determine paeonol content by high-performance liquid chromatography (HPLC). Chromatographic conditions: Use octadecylsilane bonded silica as the filler; methanol-water (55:45) as the mobile phase; detection wavelength at 274 nm. The theoretical plate number, calculated based on the paeonol peak, should not be less than 4000. For the preparation of the standard solution, take an appropriate amount of the paeonol reference substance, and prepare a solution in 50% ethanol to achieve a concentration of 70μg per 1ml. To prepare the test sample solution, take the contents of the product, mix thoroughly, grind finely, take about 0.2g, weigh it precisely, place it in a stoppered conical flask, add precisely 25ml of 50% ethanol, seal tightly, weigh, ultrasonicate (power 250W, frequency 40kHz) for 30 minutes, cool down, weigh again, make up for the lost weight with 50% ethanol, shake well, filter, and take the filtrate. Assay method: Precisely measure 10μl of both the standard solution and the test sample solution, inject into the liquid chromatograph, and measure. The content of paeonol per pill in the product should not be less than 1.8mg.  (2) Determining paeoniflorin content using high-performance liquid chromatography (HPLC). Chromatographic conditions: Use octadecylsilane bonded silica as the filler; acetonitrile-water-phosphoric acid-triethylamine (15:85:0.08:0.08) as the mobile phase; detection wavelength at 230 nm. The theoretical plate number calculated based on the paeoniflorin peak should not be less than 4000.Preparation of the standard solution: Take an appropriate amount of paeoniflorin reference substance, weigh precisely, and dissolve in methanol to prepare a solution of 40μg per 1ml.Preparation of the test sample solution: Take the contents of the product under the item of content uniformity, mix thoroughly, grind finely, take about 0.1g, weigh it precisely, place it in a stoppered conical flask, add precisely 50ml of methanol, seal tightly, weigh, ultrasonicate (power 250W, frequency 40kHz) for 30 minutes, cool down, weigh again, make up for the lost weight with methanol, shake well, filter, and collect the filtrate. Assay method: Precisely measure 10μl of both the standard solution and the test sample solution, inject into the liquid chromatograph, and perform the measurement. Each unit of the product should contain no less than 3.0mg of paeoniflorin.  (3) Determining amygdalin content in peach kernels using high-performance liquid chromatography (HPLC). Chromatographic conditions: Employ octadecylsilane bonded silica as the filler; methanol-water (20:80) as the mobile phase; detection wavelength at 218 nm. The theoretical plate number based on the amygdalin peak should not be less than 4000. Preparation of the standard solution: Take an appropriate amount of amygdalin reference substance, weigh it precisely, and dissolve in 50% ethanol to prepare a solution of 40μg per 1ml. Preparation of the test sample solution: Use the test sample solution prepared under the paeonol content determination section. Assay method: Precisely measure 10μl of both the standard solution and the test sample solution, inject into the liquid chromatograph, and perform the measurement. Each unit of the product must contain no less than 0.90mg of amygdalin. | (1) Weigh 2g of the product's contents, place in a Soxhlet extractor, add an appropriate amount of ether, heat and reflux for 2 hours, cool, collect the extract and evaporate under low temperature. Dissolve the residue in 1ml of methanol to prepare the test sample solution. Similarly, prepare a control solution from 1g of Paeonia suffruticosa bark using the same method. Conduct thin-layer chromatography (TLC). Apply 5μl of each solution on the same silica gel G TLC plate, develop with a cyclohexane-ethyl acetate (3:1) mobile phase, dry, spray with a 5% ferric chloride ethanol solution acidified with hydrochloric acid, and heat at 105°C until spots are distinctly colored. In the chromatogram of the test sample, spots corresponding to those in the control chromatogram should appear at the same positions with the same colors.  (2) Weigh 2g of the product's contents, place in a Soxhlet extractor, add an appropriate amount of methanol, heat and reflux for 2 hours, cool, and concentrate the extract to about 2ml as the test sample solution. Prepare a control solution from 1g of Paeonia lactiflora in the same manner. Apply 5μl of each solution on the same silica gel GF254 TLC plate, using a trichloromethane-methanol-water (26:14:5) bottom layer solution as the mobile phase, develop, dry, spray with an anisaldehyde solution, and heat at 105°C until spots are clearly visible. In the chromatogram of the test sample, the principal spots appearing at the same positions should match in color with those of the control chromatogram.  (3) Prepare a control solution of cinnamaldehyde in 50% ethanol at a concentration of 50μg per 1ml. Conduct gas chromatography (GC), using a capillary column coated with 5% diphenyl and 95% dimethyl polysiloxane (30m in length, 0.32mm in diameter, and 0.25μm film thickness), with a column temperature of 150°C. Inject 1μl each of the control solution and the test sample solution (from the content determination section) into the gas chromatograph. The chromatogram of the test sample should show a peak with the same retention time as that of the control sample peak. | Pharmacopoeia of the People's Republic of China |
| Huange Capsule | A | (1) Preparation of the test sample solution: Precisely weigh 10g the contents of the capsule, place into a 250ml volumetric flask, add 100ml of methanol, shake and weigh; ultrasonicate (150W power, 50Hz frequency) for 30min, leave for 24h, weigh again, make up the lost weight with methanol, shake; let stand for 12h, centrifuge at 2000rpm for 5min, collect the supernatant, filter through a 45μm filter paper, collect the filtrate; pass the filtrate directly through a column of neutral alumina-silica gel, elute with methanol, evaporate the eluate in a water bath to dryness, dissolve the residue in methanol, transfer to a 5ml volumetric flask, fill to mark with methanol, shake well, to obtain the test sample solution. Preparation of the standard solution: Take appropriate amounts of astragaloside IV and germacrone reference substances, weigh precisely, and dissolve in methanol to obtain solutions of 0.5mg/ml of astragaloside IV and 0.10mg/ml of germacrone, respectively.  (2) High-performance liquid chromatography (HPLC) conditions: Use a Waters Symmetry C18 column (4.6mm×250mm, 5μm); mobile phase of acetonitrile (A) - water (B) for gradient elution, flow rate of 1.0ml/min, column temperature between 25 to 40°C; Allerch ELSD2000 type evaporative light scattering detector, detection parameters: drift tube temperature 101°C, nitrogen flow rate 2.7L/min; detection wavelength between 200 to 215nm.  (3) Detection method: Precisely inject 10μl of the test sample solution into the HPLC, perform the analysis and calculate to determine the content of astragaloside IV and germacrone in the capsules. | Chromatographic conditions: Column: Diamonsil C18, 150mm x 4.6mm, 5μm; Mobile phase: acetonitrile-water (32:68); Column temperature: 30°C; Flow rate: 1.0 mL/min; Injection volume: 20 μL. ELSD parameters: Nebulizing gas: nitrogen; Nebulizing pressure: 3.5 Bar; Sensitivity: 6; Temperature: 40°C. Preparation of the standard solution: Take an appropriate amount of astragaloside reference substance, weigh it precisely, and dissolve in methanol to prepare a solution of 0.5 mg per 1 mL. Preparation of the test sample solution: Take the product under the item of content uniformity, grind finely, weigh about 5g precisely, place in a stoppered conical flask, add 50 mL of 80% methanol precisely, mix, weigh, ultrasonicate for 30 minutes (power 250W, frequency 40kHz), cool, weigh again, make up the lost weight with methanol, shake, filter, precisely measure 35 mL of the filtrate, concentrate to dryness. Dissolve the residue in 15 mL of water, warm slightly to dissolve, and extract three times with n-butanol saturated with ammonia solution, 40 mL each time. Combine the n-butanol extracts, wash three times with ammonia solution, 40 mL each time. Evaporate the n-butanol extract to dryness, dissolve the residue in methanol and transfer to a 10 mL volumetric flask, fill to the mark with methanol, shake well, to be used as the test sample solution. Precisely inject 10 and 30 μL of the standard solution and 20 μL of the test sample solution into the liquid chromatograph, perform the measurement, and calculate the content of astragaloside using the external standard method with a logarithmic equation. The content of astragaloside in the sample was found to be 0.271 mg per pill. | Pharmacopoeia of the People's Republic of China, doi: 10.13748/j.cnki.issn1007-7693.2007.04.014. |
| Jingui Shenqi Pill | A | Chromatographic conditions: Thermo Hypersil BDS C18 column (250mm x 4.6mm, 5μm); Mobile phase A is acetonitrile and Mobile phase B is 0.01% phosphoric acid, with gradient elution; flow rate of 1mL/min. Detection wavelengths: 0-17 min, 237 nm; 18-25 min, 233 nm; 25-40 min, 330 nm; 40-55 min, 275 nm. Column temperature: 35°C.  Preparation of the standard solution: Precisely weigh an appropriate amount of loganin, paeoniflorin, verbascoside, and paeonol reference substances, and dissolve in 80% methanol to make a mixed solution with mass concentrations of 37.82, 53.52, 76.51, and 64.70μg/mL, respectively, as the standard solution. Preparation of the test sample solution:Take an appropriate amount of the test sample, grind finely, precisely weigh 2.0g, place in a stoppered conical flask, add 50mL of 80% methanol precisely, seal, weigh, ultrasonicate for 30min, cool, reweigh, make up the lost weight with 80% methanol, shake, filter to obtain the test sample solution.  Content determination: Each 1.0g sample approximately contains loganin 0.2782mg, paeoniflorin 0.5873mg, verbascoside 0.7426mg, and paeonol 0.7440mg. | Weigh 10g of the product, grind finely, add 15ml of ether, and soak for 12 hours. Filter, evaporate the ether from the filtrate, dissolve the residue in 1ml of acetone to prepare the test sample solution. Prepare a control solution by dissolving paeonol reference substance in acetone to achieve a concentration of 1mg per 1ml. Perform thin-layer chromatography (TLC) by applying 10μl of each solution onto the same silica gel G TLC plate. Use a cyclohexane-ethyl acetate (3:1) mixture as the developing solvent. Develop, remove, air-dry, and spray with a 5% ferric chloride ethanol solution acidified with hydrochloric acid. Heat at 105°C until spots become clearly visible. In the chromatogram of the test sample, blue-brown spots should appear at the positions corresponding to those in the control chromatogram. | Pharmacopoeia of the People's Republic of China, LIN L. (2012). Determination of loganin,paeoniflorin,verbascoside and paeonol in Jinkui Shenqi Pills by HPLC. Chin. Tradit. Pat. Med. 34, 2137–2140 |
| Longbishu Capsule |  | Determining content using high-performance liquid chromatography (HPLC). Chromatographic conditions: Utilize octadecylsilane bonded silica gel as the filler; acetonitrile-water (35:65) as the mobile phase; detection wavelength at 246 nm. The theoretical plate number based on the psoralen peak should not be less than 7000.  Preparation of the standard solution: Take an appropriate amount of psoralen and isopsoralen reference substances, weigh precisely, and dissolve in methanol to prepare a mixed solution containing 10μg of each per 1ml.  Preparation of the test sample solution: Take the contents of the product under the item of content uniformity, grind finely, weigh approximately 0.3g, place in a stoppered conical flask, add precisely 25ml of methanol, seal, weigh, ultrasonicate (power 250W, frequency 50kHz) for 30 minutes, cool, reweigh, make up the lost weight with methanol, shake, filter, precisely measure 5ml of the filtrate into a 25ml volumetric flask, add 65% methanol to the mark, shake, filter, and collect the filtrate.  Assay method: Precisely inject 10μl of both the standard solution and the test sample solution into the HPLC, perform the analysis, and obtain the results. Each unit of the product must contain no less than 1.2mg of psoralen and isopsoralen, calculated as the total amount of both compounds. | (1) Take 1.5g of the product, add 30ml of ethyl acetate, heat and reflux for 1 hour, cool, filter, evaporate the filtrate to dryness, dissolve the residue in 1ml of ethyl acetate to prepare the test sample solution. Separately, prepare a mixed standard solution containing 2mg/ml each of psoralen and isopsoralen in ethyl acetate. Conduct a thin-layer chromatography (TLC) test by applying 4–8μl of each solution onto the same silica gel G TLC plate, develop with hexane-ethyl acetate (8:2) as the mobile phase, dry, spray with 1% sodium hydroxide ethanol solution, and examine under a UV lamp at 365nm. In the chromatogram of the test sample, fluorescence spots corresponding to those in the standard chromatogram should appear at the same locations.  (2) Take 2g of the product, add 30ml of ethanol, ultrasonicate for 20 minutes, filter, concentrate the filtrate to about 5ml. Load onto a column packed with activated carbon and neutral alumina (activated carbon 60–80 mesh, 0.6g; neutral alumina 100–200 mesh, 2g; mixed well and packed into a column with an inner diameter of 1cm), elute with 80% ethanol 30ml, collect the eluate, evaporate to dryness. Dissolve the residue in 10ml of hydrochloric acid solution, filter, evaporate the filtrate on a water bath to near dryness, dissolve the residue in 1ml of ethanol, shake gently, and use the supernatant as the test sample solution. Prepare a standard solution of stachydrine hydrochloride by dissolving it in ethanol to a concentration of 3mg/ml. Perform TLC by applying 5μl of each solution onto the same silica gel G TLC plate, develop with butanol-ethyl acetate-hydrochloric acid (8:1:3) as the mobile phase, dry, spray with dilute potassium iodobismuthate solution, and examine after 2 hours. In the chromatogram of the test sample, spots at the same locations as those in the standard chromatogram should display the same color. | Pharmacopoeia of the People's Republic of China |
| Liuwei Dihuang Pill | A | Determining content using high-performance liquid chromatography (HPLC). Chromatographic conditions: Use octadecylsilane bonded to silica gel as the filler; acetonitrile as mobile phase A and 0.3% phosphoric acid solution as mobile phase B, with gradient elution. Detection wavelengths for morroniside and loganin are 240nm, and for paeonol is 274nm; column temperature is 40°C. Theoretical plate numbers for morroniside and loganin peaks should not be less than 4000.  Preparation of the standard solution: Take an appropriate amount of morroniside, loganin, and paeonol reference substances, weigh precisely, and dissolve in 50% methanol to prepare a mixed solution containing 20μg each of morroniside and loganin and 45μg of paeonol per 1ml.  Preparation of the test sample solution: Cut the product, take approximately 1g, weigh precisely. Place in a stoppered conical flask, add precisely 25ml of 50% methanol, seal, weigh, reflux for 1 hour, cool, reweigh, make up the lost weight with 50% methanol, shake, filter, and collect the filtrate.  Assay method: Precisely inject 10μl of both the standard solution and the test sample solution into the HPLC, perform the analysis, and obtain the results. Each gram of the product must contain no less than 0.50mg of the total amount of morroniside and loganin, and no less than 0.70mg of paeonol. | (1) Take 6g of the product, chop finely. Add 25ml of methanol, ultrasonicate for 30 minutes, filter, evaporate the filtrate to dryness, dissolve the residue in 20ml of water, and extract twice with a 1:1 mixture of n-butanol and ethyl acetate, 20ml each time. Combine the extracts, wash with 20ml of ammonia solution, discard the ammonia, evaporate to dryness, dissolve the residue in 1ml of methanol as the test sample solution. Prepare a mixed standard solution containing 2mg/ml each of morroniside and loganin in methanol. Perform thin-layer chromatography (TLC) as per general rule 0502, applying 5μl of test solution and 2μl of standard solution onto the same silica gel G TLC plate, developing with a 3:1 mixture of chloroform and methanol, drying, spraying with 10% sulfuric acid ethanol solution, heating at 105°C until spots become clearly visible, and inspecting under UV light at 365nm. In the test sample's chromatogram, fluorescent spots should appear at the same positions as in the standard's chromatogram, showing the same colors.  (2) Take 6g of the product, chop finely, add 4g of diatomaceous earth, mix well. Add 40ml of ether, reflux for 1 hour, filter, evaporate the ether from the filtrate, dissolve the residue in 1ml of acetone as the test sample solution. Prepare a standard solution of paeonol in acetone, containing 1mg/ml. Perform TLC, applying 10μl of each solution onto the same silica gel G TLC plate, developing with a 3:1 mixture of cyclohexane and ethyl acetate, drying, spraying with 5% hydrochloric acid ferric chloride ethanol solution, heating until spots become clearly visible. In the test sample's chromatogram, spots should appear at the same positions as in the standard's chromatogram, showing the same colors. | Pharmacopoeia of the People's Republic of China |
| Lingze Tablet | A | Determining content using high-performance liquid chromatography (HPLC). Chromatographic condition: Use octadecylsilane bonded to silica gel as the stationary phase; acetonitrile as mobile phase A, and water as mobile phase B, with gradient elution; detection wavelength at 216nm. The theoretical plate number based on the germacrone peak should not be less than 5000.  Preparation of the standard solution: Take an appropriate amount of germacrone and furandiene reference substances, weigh precisely, and dissolve in anhydrous ethanol to prepare a mixed solution containing 20μg of each per 1ml.  Preparation of the test sample solution: Take 20 tablets of the product, weigh precisely, grind finely, take about 3.5g, place in a 500ml round-bottom flask, add 200ml of water and a few glass beads, attach a volatile oil extractor, fill the scale part with water from the top of the determinator until it overflows into the flask, add 2ml of ethyl acetate, and determine volatile oils, maintaining a gentle boil for 2 hours, cool, separate the ethyl acetate layer, add 2ml of ethyl acetate and extract once more in the same manner, combine the ethyl acetate layers, transfer to a 25ml volumetric flask, fill to the mark with anhydrous ethanol, shake well, precisely measure 2ml, place in a 20ml volumetric flask, fill to the mark with anhydrous ethanol, shake, filter, and collect the filtrate.  Assay method: Precisely inject 5μl of both the standard solution and the test sample solution into the HPLC, perform the analysis, and obtain the results. Each tablet of the product must contain no less than 0.50mg of germacrone, and no less than 0.50mg of furandiene. | (1) Take 4 tablets of the product, grind finely, dissolve in 30ml of boiling water, centrifuge, and take the precipitate. Add 20ml of acetone, ultrasonicate for 30 minutes, filter, evaporate the filtrate to dryness, and dissolve the residue in 1ml of acetone as the test sample solution. Similarly, prepare a control material solution from 1g of Xylaria nigripes powder following the same procedure. Conduct thin-layer chromatography (TLC), applying 5μl of each solution onto a silica gel G TLC plate prepared with an equal volume mixture of 0.4% sodium carboxymethyl cellulose solution and 3.85% disodium hydrogen phosphate solution. Use a developing solvent of chloroform-ethyl acetate-isopropanol-water-concentrated ammonia solution (4:1:1:0.1:0.1), develop, dry, and inspect immediately under UV light at 365nm. Fluorescent spots appearing at the same positions as the control material should be visible in the test sample chromatogram.  (2) Take 4 tablets of the product, grind finely, extract with 20ml of ether by shaking for 20 minutes, filter, evaporate the filtrate to dryness, and dissolve the residue in 2ml of ethyl acetate as the test sample solution. Prepare a control solution of curcumol by dissolving it in ethyl acetate to achieve a concentration of 1mg/ml. Perform TLC and apply 5μl of each solution onto a silica gel G TLC plate, using petroleum ether (60-90°C)-ethyl acetate (12:1) as the developing solvent, develop, dry, spray with 1% vanillin-sulfuric acid solution, heat at 105°C until spots become clearly visible, and inspect in daylight. Spots of the same color appearing at the same positions as the control should be visible in the test sample chromatogram.  (3) Take 2 tablets of the product, grind finely, dissolve in 20ml of boiling water, filter while hot, add 1ml of concentrated ammonia to the filtrate, extract twice with chloroform, 20ml each time, combine the chloroform extracts, evaporate the solvent to dryness, and dissolve the residue in 0.5ml of chloroform as the test sample solution. Prepare the control solution of peimine and peiminine, each at a concentration of 0.5mg/ml in chloroform. Conduct TLC , applying 5μl of each solution onto a silica gel G TLC plate, using ethyl acetate-methanol-concentrated ammonia (17:2:1) as the developing solvent, develop, dry, sequentially spray with dilute potassium iodobismuthate and sodium nitrite ethanol solutions, and inspect in daylight. Spots of the same color appearing at the same positions as the control should be visible in the test sample chromatogram. | Pharmacopoeia of the People's Republic of China |
| Qianliekang Tablet (Pulean Tablet) | A | Determining content by high-performance liquid chromatography (HPLC). Chromatographic conditions: Use octadecylsilane bonded silica gel as the filler; methanol-0.4% phosphoric acid solution (50:50) as the mobile phase; detection wavelength at 360nm. The theoretical plate number based on the quercetin peak should not be less than 2500.  Preparation of the standard solution: Take an appropriate amount of quercetin and kaempferol reference substances, weigh precisely, and dissolve in methanol to prepare a mixed solution containing 12μg of quercetin and 25μg of kaempferol per 1ml.  Preparation of the test sample solution: Take the product, grind finely, weigh approximately 0.6g, add 40ml of a mixed solution of 25% hydrochloric acid in methanol (1:6), reflux at 80°C for 30 minutes, quickly cool to room temperature, transfer to a 50ml volumetric flask. Wash the reflux flask with 5ml of methanol, combine the washings in the same volumetric flask, and dilute to the mark with methanol, shake well, filter, and collect the filtrate.  Assay method: Precisely inject 10μl of both the standard solution and the test sample solution into the HPLC, perform the analysis, and obtain the results. Each tablet of the product must contain no less than 1.0mg of the total amount of quercetin and kaempferol, calculated based on rapeseed pollen. | (1) Take 2 tablets of the product, grind finely, add 25ml of methanol, ultrasonicate for 10 minutes, filter, and concentrate the filtrate to 10ml to prepare the test sample solution. Similarly, prepare a control material solution from 1g of rapeseed pollen using the same method. Conduct thin-layer chromatography (TLC), applying 5μl of each solution onto the same silica gel G TLC plate, using ethyl acetate-butanol-methanol-water (5:3:1:1) as the developing solvent, develop, remove, air-dry, spray with 3% aluminum chloride ethanol solution, heat at 105°C for about 2 minutes, and inspect under UV light at 365nm. In the chromatogram of the test sample, fluorescent spots appearing at the same positions as in the control material's chromatogram should be visible.  (2) Take 2 tablets of the product, grind finely, add 10ml of petroleum ether (60–90°C), ultrasonicate for 10 minutes, discard the petroleum ether, add 10ml of acetone to the residue, ultrasonicate for 30 minutes, centrifuge, and use the supernatant as the test sample solution. Prepare a control material solution from 1g of rapeseed pollen using the same method. Additionally, prepare a control solution of β-sitosterol by dissolving it in acetone to achieve a concentration of 0.1mg/ml. Perform TLC, applying 5μl of each of the three solutions onto the same silica gel G TLC plate, using cyclohexane-ethyl acetate-acetic acid (9:2:0.2) as the developing solvent, develop, remove, air-dry, spray with 5% phosphomolybdic acid ethanol solution, heat at 105°C until spots become clearly visible. In the chromatogram of the test sample, spots of the same color appearing at the same positions as those in the control material's and control substance's chromatograms should be visible. | Pharmacopoeia of the People's Republic of China |
| Qianlie Beixi Capsule | A | Chromatographic conditions: Use a HypersiL C18 column (4.6mm x 150mm, 5μm), with a mobile phase of methanol-0.2% phosphoric acid solution (47:53), column temperature at 40°C, flow rate at 1.0mL/min, detection wavelength at 370nm, and injection volume of 10μL.  Preparation of sample solution: Take the contents of 10 pills, mix well, take approximately 1g, weigh precisely, place in a stoppered conical flask, add 25mL of 70% methanol, seal, ultrasonicate for 10 minutes, shake, filter, precisely measure 10mL of the filtrate into a round-bottom flask, add 2.5mL of 25% hydrochloric acid solution, reflux in a water bath for 30 minutes, cool, transfer into a 25mL volumetric flask, fill to the mark with methanol, shake, filter through a 0.45μm filter membrane, and use the filtrate as the test sample solution. Take an appropriate amount of quercetin and kaempferol reference substances and prepare the standard solution in the same manner.  Sample determination: Precisely inject 10μL each of the standard and sample solutions into the liquid chromatograph under the aforementioned chromatographic conditions, record the chromatograms, and calculate the contents of quercetin and kaempferol in the sample using the external standard method. The results indicate that each pill contains 0.331mg of quercetin and 0.327mg of kaempferol. | Preparation of the Test Sample Solution: Take 5g of the product contents, add 50mL of ethyl acetate, ultrasonicate for 30 minutes, and then filter. Treat the filter residue with 50mL of n-butanol, ultrasonicate again for 30 minutes, and filter. Evaporate the filtrate to dryness, dissolve the residue in 1mL of methanol to serve as the test sample solution. Prepare a negative control solution from a sample lacking Vaccaria segetalis using the same method. Preparation of the Control Material Solution: Take 0.5g of finely ground Vaccaria segetalis control material and prepare the control solution in the same manner.  Thin-Layer Chromatography (TLC): Apply 10μL of each solution onto the same silica gel G TLC plate, which uses sodium carboxymethyl cellulose as the adhesive. Use butanol saturated with water, ethyl acetate, and acetic acid (3:1:0.2) as the developing solvent. After development, remove the plate, air dry, spray with a 10% sulfuric acid ethanol solution, and heat at 105°C until the spots become clearly visible. Examine under UV light at 365nm. In the test sample chromatogram, fluorescent spots should appear at the same positions as in the control material's chromatogram, with no interference observed in the negative control. | Pharmacopoeia of the People's Republic of China, Lan Y. (2003). Qualitative and Quantitative Studies on Qianliebeixi Capsules. China J. Chin. Mater. Medica, 36–38. |
| Qianlieshu Pill | A | Determination by high-performance liquid chromatography (HPLC). Chromatographic conditions: Use octadecylsilane bonded to silica gel as the filler; methanol-water (45:55) as the mobile phase; detection wavelength at 274nm. Theoretical plate number based on the paeonol peak should not be less than 3500.  Preparation of the standard solution: Take an appropriate amount of paeonol reference substance, weigh precisely, and dissolve in methanol to prepare a solution containing 20μg per 1ml.  Preparation of the test sample solution: Take the product, chop finely, mix well, take approximately 2g, weigh precisely, place in a stoppered conical flask, add precisely 50ml of methanol, seal, weigh, ultrasonicate (power 300W, frequency 40kHz) for 40 minutes, cool, reweigh, make up for the lost weight with methanol, shake, filter, and collect the filtrate.  Assay method: Precisely inject 10μl of both the standard solution and the test sample solution into the liquid chromatograph, perform the analysis, and obtain the results. The content of paeonol in the product must contain no less than 3.2mg. | (1) Take 10g of the product, chop finely, add 50ml of ether, ultrasonicate for 20 minutes, filter, evaporate the filtrate to dryness, and dissolve the residue in 1ml of methanol as the test sample solution. Prepare a control solution by dissolving ursolic acid reference substance in methanol to achieve a concentration of 1mg per 1ml. Conduct a thin-layer chromatography (TLC) test by applying 10μl of the test sample solution and 5μl of the control solution onto the same silica gel G TLC plate, using cyclohexane-chloroform-ethyl acetate (20:5:7) as the developing solvent. Develop, remove, air-dry, spray with a 10% sulfuric acid ethanol solution, and heat at 80°C until spots become clearly visible. Spots of the same color appearing at the same positions as the control in the test sample chromatogram should be visible.  (2) Take 20g of the product, chop finely, add 30ml of ether, ultrasonicate for 15 minutes, filter, evaporate the filtrate to dryness, and dissolve the residue in 1ml of ethyl acetate as the test sample solution. Prepare a control material solution from 0.5g of Atractylodes lancea in the same manner. Perform a TLC test by applying 10μl of the newly prepared test sample solution and 5μl of the control material solution onto the same silica gel G TLC plate, using petroleum ether (60–90°C) as the developing solvent. Develop, remove, air-dry, spray with a 5% solution of p-dimethylaminobenzaldehyde in 10% sulfuric acid ethanol, and heat at 80°C until spots become clearly visible. Spots of the same color appearing at the same positions as the control material in the test sample chromatogram should be visible. | Pharmacopoeia of the People's Republic of China |
| Qianlie Shutong Capsule | A | Determination by high-performance liquid chromatography (HPLC). Chromatographic conditions: Use octadecylsilane bonded silica gel as the filler; 0.1% phosphoric acid solution-acetonitrile (75:25) as the mobile phase; detection wavelength at 270nm. The theoretical plate number based on the berberine chloride peak should not be less than 3000.  Preparation of the standard solution: Take an appropriate amount of berberine hydrochloride reference substance, weigh precisely, and dissolve in methanol to prepare a solution containing 40µg per 1ml.  Preparation of the test sample solution: Take the contents of the product, mix well, grind finely, weigh 0.1g precisely, place in a stoppered conical flask, add precisely 50ml of methanol, seal, weigh, ultrasonicate for 20 minutes, cool, reweigh and make up for the lost weight with methanol, filter, and collect the filtrate.  Assay method: Precisely inject 10µl of both the standard solution and the test sample solution into the liquid chromatograph, perform the analysis, and obtain the results. Each tablet of the product must contain no less than 2.0mg of berberine hydrochloride. | (1) Take 2g of the product, chop finely, add 30ml of ether, ultrasonicate for 20 minutes, filter, and evaporate the filtrate to dryness. Dissolve the residue in 1ml of anhydrous ethanol to prepare the test sample solution. Prepare control material solutions of Angelica sinensis and Ligusticum chuanxiong each with 0.5g, similarly treated with 20ml of ether. Perform thin-layer chromatography (TLC), applying 10µl of each solution onto the same silica gel G TLC plate. Develop using a solvent system of hexane-ethyl acetate (9:1), dry, inspect under UV light at 365nm. The test sample chromatogram should show fluorescent spots at the same positions as the control material chromatograms with the same color.  (2) Take the ether-extracted drug residue from step (1), add 30ml of methanol, ultrasonicate for 20 minutes, filter, and evaporate the filtrate to dryness. Dissolve the residue in 10ml of water and extract twice with water-saturated n-butanol. Combine the n-butanol layers, wash twice with ammonia solution (each time with 30ml) and then twice with water (each time with 30ml), evaporate the n-butanol to dryness, dissolve the residue in 1ml of methanol, and pass through a neutral alumina column (6g, 100-200 mesh), eluting with 50ml of methanol. Collect the eluate, evaporate to dryness, and dissolve the residue in 1ml of methanol as the test sample solution. Prepare a paeoniflorin control solution with methanol to make a 0.3mg/ml solution. Perform TLC as described, applying 5µl of each solution onto a silica gel G TLC plate, developing with the bottom layer of chloroform-methanol-water (13:7:2) mixture kept below 10°C for 12 hours, dry, spray with 10% sulfuric acid ethanol solution, and heat with hot air until spots are clearly visible. The test sample chromatogram should show spots of the same color at the same positions as the control. | Pharmacopoeia of the People's Republic of China |
| Qianlie Tongyu Capsule | A | Determination by high-performance liquid chromatography (HPLC). Chromatographic conditions: Utilize octadecylsilane bonded to silica gel as the filler; acetonitrile-0.04% phosphoric acid (15:85) as the mobile phase; detection wavelength at 230nm. The theoretical plate number, based on the paeoniflorin peak, should not be less than 2000.  Preparation of the standard solution: Precisely weigh an appropriate amount of paeoniflorin reference substance dried to constant weight at 80°C, dissolve in methanol to prepare a solution containing 0.15mg per 1ml.  Preparation of the test sample solution: Take the contents of the product, mix well, weigh 1g precisely, place in a stoppered conical flask, add precisely 50ml of water, weigh, ultrasonicate for 30 minutes, cool, reweigh, make up for the lost weight with water, filter, and collect the filtrate.  Assay method: Precisely inject 5μl each of the standard solution and the test sample solution into the liquid chromatograph, perform the analysis, and obtain the results. Each tablet of the product must contain no less than 3.0mg of paeoniflorin. | (1) Take 1g of the product, add 4ml of ethanol, and macerate for 4 hours at a warm temperature, cool, then filter. The filtrate serves as the test sample solution. Prepare a standard solution of paeoniflorin by dissolving it in ethanol to achieve a concentration of 1mg per 1ml. Perform thin-layer chromatography (TLC) by applying 2μl of each solution onto the same silica gel G TLC plate, using chloroform-ethyl acetate-methanol-formic acid (40:5:10:0.2) as the developing solvent. After development, remove, air-dry, and spray with a 5% vanillin sulfuric acid solution, blow with hot air until spots become clearly visible. In the chromatogram of the test sample, blue-purple spots should appear at the same positions as those in the standard chromatogram.  (2) Take 2g of the product, add 10ml of 70% ethanol, ultrasonicate for 15 minutes, filter, and use the filtrate as the test sample solution. Prepare a control material solution from 1g of peach kernel using the same method. Perform TLC by applying 1μl of the test sample solution and 2μl of the control material solution onto the same silica gel G TLC plate, using n-butanol-acetic acid-water (3:1:1) as the developing solvent. After development, remove, air-dry, and spray with a 2% indantrione acetone solution, and heat at 105℃ until spots become clearly visible. In the chromatogram of the test sample, spots of the same color should appear at the same positions as those in the control material chromatogram. | Pharmacopoeia of the People's Republic of China |
| Qianliexin Capsule | A | Determination by high-performance liquid chromatography (HPLC). Chromatographic conditions: Use octadecylsilane bonded silica gel as the filler; methanol-water (55:45) as the mobile phase; detection wavelength at 249nm. The theoretical plate number based on the imperatorin peak should not be less than 8000.  Preparation of the standard solution: Take an appropriate amount of imperatorin reference substance, weigh precisely, and dissolve in methanol to prepare a solution containing 10μg per 1ml.  Preparation of the test sample solution: Take the contents of the product, mix well, grind finely, weigh approximately 0.5g, place in a stoppered conical flask, add precisely 25ml of methanol, seal, weigh, ultrasonicate for 30 minutes (power 500W, frequency 40kHz), cool, reweigh, make up for the lost weight with methanol, shake, filter, and collect the filtrate.  Assay method: Precisely inject 10μl of the standard solution and 20μl of the test sample solution into the liquid chromatograph, perform the analysis, and obtain the results. Each unit of the product must contain no less than 60μg of imperatorin. | (1) Take 10g of the product, grind finely, add 40ml of methanol, ultrasonicate for 30 minutes, filter, evaporate the filtrate to dryness, dissolve the residue in 20ml of water, and extract thrice with water-saturated n-butanol, each time using 20ml. Combine the n-butanol extracts, wash with 20ml of water-saturated n-butanol, evaporate the n-butanol to dryness, and dissolve the residue in 1ml of methanol to prepare the test sample solution. Prepare a standard solution of paeoniflorin by dissolving it in methanol to achieve a concentration of 1mg per 1ml. Perform thin-layer chromatography (TLC), applying 10μl of each solution onto the same silica gel G TLC plate, using chloroform-ethyl acetate-methanol-formic acid (40:5:10:0.2) as the developing solvent. Develop, remove, air-dry, spray with a 5% vanillin sulfuric acid solution, and heat at 105℃ until spots become clearly visible. In the test sample's chromatogram, spots of the same color should appear at the same positions as in the standard's chromatogram.  (2) Take 1g of the product, grind finely, add 5ml of ether, ultrasonicate for 10 minutes, let stand, and use the supernatant as the test sample solution. Prepare a control material solution from 0.1g of commiphora myrrha in the same manner. Conduct TLC, applying 5μl of each solution onto the same silica gel G TLC plate, using petroleum ether (60-90℃)-ethyl acetate (9:1) as the developing solvent. Develop, remove, air-dry, spray with a 5% vanillin sulfuric acid solution, and heat until spots become clearly visible. In the test sample's chromatogram, the main spots should appear at the same positions as those in the control material's chromatogram, showing the same color. | Pharmacopoeia of the People's Republic of China |
| Saw Palmetto Extract | A | Chromatographic Conditions: DB-5 capillary column (30m x 0.25mm, 0.25μm); carrier gas is nitrogen; the temperature starts at 100°C for 3 minutes, then increases at 20°C/min to 100°C, for 8 minutes, then at 40°C/min to 120°C, for 5 minutes; carrier gas flow rate is 1mL/min; split ratio is 20:1; injection volume is 1μL; the injector temperature is 200°C; detector temperature is 250°C.  Preparation of internal standard solution: Weigh an appropriate amount of methyl heptadecanoate and dissolve in n-hexane, shake to make a concentration of 10.000mg/mL.  Preparation of standard solution: Weigh fatty acid methyl ester standards including methyl hexanoate, methyl octanoate, methyl decanoate, methyl laurate, methyl myristate, methyl palmitate, methyl palmitoleate, methyl stearate, methyl oleate, methyl linoleate, and methyl linolenate in appropriate amounts, dissolve in n-hexane, shake well, as the standard solution with concentrations of 1.036, 0.892, 1.020, 12.104, 4.828, 3.626, 0.126, 0.840, 12.282, 2.378, and 0.356mg/mL, respectively. Accurately measure 2.5mL into a 5.0mL volumetric flask, dilute to mark with n-hexane, add 1.0mL internal standard solution, mix well.  Preparation of test sample solution: Take 100mg of the capsule content into a 25mL conical flask, add 3mL of 5% methanolic sulfuric acid, heat in an oil bath at 100°C for 2 hours with continuous stirring, cool. Sequentially add 10mL of purified water, 1g of sodium chloride, 1mL of internal standard solution, and 5mL of n-hexane, shake well, wait to separate, take the n-hexane layer, filter through a 0.22μm micropore filter.  The average content of each fatty acid in the capsules is measured as follows: hexanoic acid: 1.77mg per capsule, octanoic acid: 3.32mg per capsule, decanoic acid: 3.52mg per capsule, lauric acid: 42.50mg per capsule, myristic acid: 15.98mg per capsule, palmitic acid: 12.77mg per capsule, palmitoleic acid: 0.18mg per capsule, stearic acid: 1.77mg per capsule, oleic acid: 41.59mg per capsule, linoleic acid: 6.98mg per capsule, linolenic acid: 0.92mg per capsule. | Chromatographic Conditions: Hypersil C18 column (250mm x 4.6mm, 5μm), with a mobile phase of methanol:water (94:6) in isocratic elution. The flow rate is set at 1mL/min, the column temperature is maintained at 30°C, and the injection volume is 10 μL.  Preparation of standard solution: Accurately weigh an appropriate amount of β-sitosterol standard and dissolve in methanol. Shake well to make a concentration of 0.500mg/mL as the standard reserve solution.  Preparation of test sample solution:Take 1.000g of the content of saw palmetto extract softgel capsules and place it in a 100mL conical flask, accurately weighed. Add 20mL of potassium hydroxide methanol solution, reflux heat for 2 hours, cool, then transfer the extract to a 125mL separating funnel, wash with water and combine the washings. Extract the solution with ether three times, each time using 30mL, and combine the ether extracts. Wash the combined ether solution with water three times, each time using 30mL. Evaporate the ether to dryness, dissolve the residue in methanol, and make up to 5mL in a volumetric flask to obtain the test sample solution.  The results show that the average content of β-sitosterol per capsule in the product is 3.136mg. | Pharmacopoeia of the People's Republic of China, YANG X. (2015). Quality standard of Sabal fruit extract soft capsules. Chin. J. New Drugs 24, 2854–2864. |
| Wenglitong Capsule | A | Take 5g of the product, weigh precisely, and mix with 2.5g of diatomaceous earth. Add 2ml of concentrated ammonia solution gradually while mixing. Place the mixture into a filter paper tube within a Soxhlet extractor and reflux with chloroform for 6 hours. Recover the chloroform solution, dissolve the residue in chloroform, and quantitatively transfer to a 10ml volumetric flask to serve as the test sample solution. Prepare a control solution of peiminine by dissolving it in chloroform to achieve a concentration of 0.5mg per 1ml. Conduct a thin-layer chromatography (TLC) test by applying 15μl of the test sample solution and 3μl, 5μl of the control solution onto the same silica gel G TLC plate, using chloroform-ethyl acetate-methanol-ammonia (2:6.5:1:0.5) as the developing solvent. After development, remove, air-dry, and spray with modified potassium bismuth iodide reagent. Cover the TLC plate with a glass plate of the same size, secure the edges with tape, and scan by TLC at wavelengths λS=500nm and λR=590nm. Measure the integral absorbance values of the test and control solutions and calculate accordingly. Each unit of the product must contain no less than 0.072mg of peiminine. | (1) Take 10g of the product, add 30ml of ethanol, reflux for 30 minutes, cool, filter, and concentrate the filtrate to about 5ml on a water bath as the test sample solution. Prepare a standard solution of gardenoside by dissolving it in ethanol to achieve a concentration of 0.5mg per 1ml. Conduct a thin-layer chromatography test by applying 10μl of each solution onto the same silica gel G TLC plate, using chloroform-methanol-25% ammonia solution (4:1:0.1) as the developing solvent. Develop, remove, air-dry, and spray with a 10% sulfuric acid ethanol solution, and heat at 105°C for about 10 minutes. In the chromatogram of the test sample, spots of the same color should appear at the same positions as those in the standard's chromatogram.  (2) Take 2g of the product, add 25ml of water, stir, centrifuge, and take the supernatant to which 5ml of 10% sodium hydroxide solution is added, shake well, extract twice with water-saturated n-butanol, each time using 30ml, combine the n-butanol extracts, wash with 40ml of water. After dehydrating the n-butanol solution with an adequate amount of anhydrous sodium sulfate, evaporate on a water bath, dissolve the residue in 1ml of methanol as the test sample solution. Prepare a standard solution of astragaloside by dissolving it in methanol to achieve a concentration of 1mg per 1ml. Conduct a TLC test by applying 2μl of each solution onto the same silica gel G TLC plate, using the lower phase of chloroform-ethyl acetate-methanol-water (20:40:20:10) kept below 10°C as the developing solvent. Develop, remove, air-dry, spray with a 10% sulfuric acid ethanol solution, and heat at 105°C for about 10 minutes. In the chromatogram of the test sample, spots of the same color should appear at the same positions as those in the standard's chromatogram. | Pharmacopoeia of the People's Republic of China |
| Xialiqi Capsule | A | Chromatographic conditions involve using a Shield RP18 column (100mm x 2.1mm, 1.7μm); the mobile phase consists of acetonitrile (A) and 0.2% formic acid aqueous solution (B), with gradient elution. The flow rate is set at 0.2ml/min, the detection wavelength at 240 nm, column temperature at 35°C, and injection volume at 1μl.  Preparation of the test sample solution: Take an appropriate amount of the capsule contents, grind finely, weigh about 1g precisely, place in a stoppered conical flask, precisely add 25ml of 80% methanol, weigh, ultrasonicate for 30 minutes, cool, weigh again, and make up for the lost weight with 80% methanol, shake well to obtain the solution.  Preparation of the standard solution: Precisely weigh appropriate amounts of calycosin-7-glucoside, specnuezhenide, palmatine hydrochloride, berberine hydrochloride, and rosmarinic acid reference substances, place them in the same 10ml volumetric flask, dissolve to the mark with methanol, shake well, to prepare a mixed standard solution with concentrations of 0.396, 1.156, 0.572, 0.740, and 1.578mg/ml, respectively.  The test sample solution is then subjected to chromatographic analysis under the specified conditions, and the content of the compounds is calculated based on the chromatographic results. The contents of calycosin-7-glucoside, specnuezhenide, palmatine hydrochloride, berberine hydrochloride, and rosmarinic acid in the sample are found to be 0.351, 0.216, 0.162, 0.237, and 0.853mg, respectively. | Chromatographic conditions: The chromatographic column is Waters Acquity UPLCR BEH Shield RP18 (2.1x100 mm, 1.7 μm); the mobile phase consists of acetonitrile and 0.1% phosphoric acid solution, with gradient elution; the detection wavelength is set at 280 nm; the column temperature is 35°C; and the flow rate is 0.2ml/min.  Preparation of the test sample solution: Take approximately 1g of the capsule contents, grind finely, weigh accurately, place in a stoppered conical flask, add 25ml of 70% methanol, weigh again, ultrasonicate for 30 minutes (power 250 W, frequency 45kHz), cool down, weigh once more, and make up the lost weight with 70% methanol. Shake well and filter to obtain the solution.  Preparation of the standard solution: Weigh appropriate amounts of the standard substances berberine, palmatine, and cinnamaldehyde. Dissolve in methanol to obtain standard solutions with concentrations of 0.1984 mg/ml, 0.8312 mg/ml, and 0.5876 mg/ml, respectively.  Determination of the sample content: Take the capsule sample, extract following the aforementioned steps, and measure according to the described conditions. Calculate the contents of palmatine, berberine, and cinnamaldehyde, which are found to be 0.2040 mg/g, 0.3381 mg/g, and 0.1898 mg/g, respectively. | Pharmacopoeia of the People's Republic of China, Gao X. (2016). Simultaneous Determination of Five Active Ingredients in Xialiqi Capsule by UPLC. China Pharm. 27, 2543–2545. |
| Zegui Longshuang Capsule | A | Approximately 8g of the product is dried in a phosphorus pentoxide desiccator for 12 hours, then accurately weighed and placed in a Soxhlet extractor. An appropriate amount of anhydrous ether is added, and the product is reflux-extracted for 5 hours. The ether solution is collected in a pre-weighed evaporation dish that has been dried to constant weight, and evaporate the ether. The residue is dried again in a phosphorus pentoxide desiccator for 18 hours, weighed precisely, and the content of ether soluble extractives is calculated. The product should contain no less than 0.24% of ether soluble extractives.  The dried residue obtained is soaked twice with petroleum ether (30-60°C), each time using 20ml of petroleum ether for about 2 minutes, discarding the petroleum ether afterwards. The residue is then dissolved in a suitable amount of an anhydrous ethanol-chloroform (3:2) mixture with mild heat, and the solution is transferred to a 2ml volumetric flask and diluted to the mark with the aforementioned mixture, shaken well, to serve as the test sample solution. A standard solution of ursolic acid is prepared by dissolving it in anhydrous ethanol to make a solution containing 0.5mg per 1ml. Thin-layer chromatography (TLC) is performed by precisely applying 5ul of the test sample solution and 2ul and 4ul of the standard solution onto the same silica gel G TLC plate, using a developing solvent system of cyclohexane-chloroform-ethyl acetate-acetic acid (20:5:8:1). After development, removed, air-dried, and sprayed with a 10% sulfuric acid ethanol solution, then heated at 110°C for about 2-3 minutes until spots become clearly visible. The plate is cooled, covered with a glass plate of the same size, and secured with tape around the edges. The TLC plate is then scanned at wavelengths of λS=520nm and λR=700nm, measuring the integral absorbance values of the test sample and standard, from which the content is calculated. Each unit of the product must contain no less than 16.7ug of ursolic acid. | (1) Take 8g of the product content, add 50ml ethanol, reflux heat for 1 hour, filter, and use the filtrate as the test sample solution. Prepare a standard solution of cinnamaldehyde by dissolving it in ethanol to achieve a concentration of 0.5μl per 1ml. Conduct thin-layer chromatography (TLC) by applying 5μl of each solution onto the same silica gel G TLC plate, using petroleum ether (60-90°C) - ethyl acetate (17:3) as the developing solvent. Develop, remove, air-dry, and spray with 0.1% 2,4-dinitrophenylhydrazine ethanol solution. In the chromatogram of the test sample, spots of the same orange-yellow color should appear at the same positions as those in the standard chromatogram.  (2) Take 1.5g of the product content, add 20ml methanol, ultrasonicate for 20 minutes, filter, evaporate the filtrate to dryness, and dissolve the residue in 1ml of anhydrous ethanol to form the test sample solution. Prepare a control material solution from 1g of Gleditsia sinensis using the same method. Conduct TLC by applying 5μl of the test sample solution and 10μl of the control material solution onto the same silica gel G TLC plate, using ethyl acetate - butanone - methanol - water (5:3:1:1) as the developing solvent. Develop, remove, air-dry, and inspect under UV light at 365nm. In the chromatogram of the test sample, spots of the same pale blue fluorescence should appear at the same positions as those in the control material chromatogram. | Pharmacopoeia of the People's Republic of China |

# File S5: Details of the included studies.

| **Study ID** | **Sample Size (E/C)** | **Age (Year, E/C)** | **Consistent baseline** | **Intervention in experimental group (WM+poly-herbal TCM formulations)** | **Intervention in control group (WM)** | **Course of Treatment (Weeks)** | **Outcomes** | **Adverse Drug Reactions** |
| --- | --- | --- | --- | --- | --- | --- | --- | --- |
| Zhao H 2019 | 56/56 | 70.12±3.86/69.87±3.56 | Y | GZFL 3 pills; tamsulosin hydrochloride 0.2mg po qd; finasteride 1mg po qd | Tamsulosin hydrochloride 0.2mg po qd; finasteride 1mg po qd | 12 | ①②③④ | Detailed description |
| Zhang YL 2012 | 30/30 | 67.71±22.57/68.07±21.27 | Y | GZFL 3 pills po tid; tamsulosin hydrochloride 0.2mg po qn | Tamsulosin hydrochloride 0.2mg po qn | 12 | ①②③④ | Detailed description |
| Yu SC 2017 | 40/40 | 43-78(64.6±11.2)/38-80(63.7±10.6) | Y | GZFL 6g po bid; tamsulosin hydrochloride 0.2mg po qd | Tamsulosin hydrochloride 0.2mg po qd | 4 | ①④⑤⑥ | NR |
| Yuan KN 2021 | 60/60 | 55-80(68.23±2.46)/56-80(68.86±2.45) | Y | HE 4 pills po tid; tamsulosin hydrochloride 0.2mg po bid; finasteride 5mg po qn | Tamsulosin hydrochloride 0.2mg po bid; finasteride 5mg po qn | 6 | ① | NR |
| Sun J 2019 | 50/50 | 60-84/61-85 | Y | HE 4 pills po tid; tamsulosin hydrochloride 0.2mg po bid; finasteride 5mg po qd | Tamsulosin hydrochloride 0.2mg po bid; finasteride 5mg po qn | 6 | ①②③④⑤ | Detailed description |
| Cheng W 2023 | 50/50 | 62.18±8.24/64.34±7.21 | Y | HE 4 pills po tid; tamsulosin hydrochloride 0.2mg po bid; dutasteride 0.5mg po qd | Tamsulosin hydrochloride 0.2mg po bid; dutasteride 0.5mg po qd | 6 | ①②④⑤⑥ | NR |
| Zhi QM 2020 | 33/33 | 50-73(64.15±2.31)/50-72(63.27±1.45) | Y | JGSQ 4 pills po bid; finasteride 5mg po qd | Finasteride 5mg po qd | 24 | ②④⑤⑥ | NR |
| Chen H 2014 | 47/45 | 67.3±5.3/66.7±5.7 | Y | JGSQ 3g po qd; terazosin 2mg po qd, finasteride 5mg po qd | Terazosin tablet 2mg po qd, finasteride 5mg po qd | 24 | ②⑤⑥ | NR |
| Chen H 2013 | 57/55 | 67.0±5.36/66.7±5.64 | Y | JGSQ 3g po qn; terazosin 2mg po qn; finasteride 5mg po qn | Terazosin 2mg po qn; finasteride 5mg po qn | 12 | ②⑤⑥ | NR |
| Liao XX 2016 | 42/43 | 60-80(69.29±8.76)/60-80(69.38±8.95) | Y | JGSQ 6g po bid; finasteride 5mg po qd | Finasteride 5mg po qd | 24 | ①②③④⑥ | NR |
| Xu ZC 2021 | 30/30 | 60-86(68.88±4.03)/58-85(69.00±4.10) | Y | JGSQ 3g po qn; finasteride tablet 5mg po qd; terazosin tablet 2mg po qd | Finasteride tablet 5mg po qd; terazosin tablet 2mg po qd | 8 | ①②④⑤⑥ | Detailed description |
| Liu BX 2013 | 17/18 | 62.32±7.18/61.47±5.95 | Y | JGSQ 6g po bid; doxazosin 4mg po qn | Doxazosin 4mg po qn | 12 | ②③ | NR |
| Zhang Y 2015 | 52/50 | 56.8±7.7/54.2±8.6 | Y | JGSQ 6g po bid; tamsulosin 0.2mg po qd | Tamsulosin 0.2mg po qd | 12 | ② | NR |
| Zhang ZL 2009 | 60/60/30 | 60-80 | Y | Group1: JGSQ 8 pills po tid; finasteride 5mg po qd； Group2: LWDH 8 pills po tid; finasteride 5mg po qd | Finasteride 5mg po qd | 12 | ②③④⑤⑥ | NR |
| Meng F 2017 | 35/35 | 75.36±6.54/76.38±2.89 | Y | LBS 1.8g po qd; finasteride 5mg po qd | Finasteride 5mg po qd | 12 | ① | NR |
| Luo JG 2009 | 30/20 | 50-90/53-85 | Y | LBS 3 pills po bid; cernilton tablet 1 pill bid | Cernilton tablet 1 pill bid | 8 | ①④⑤⑥ | NR |
| Ji HL 2018 | 50/50 | 60-75(65.8±2.28)/60-75(64.24±2.17) | Y | LBS 3 pills po bid; tamsulosin 0.2mg po qn | Tamsulosin 0.2mg po qn | 8 | ① | Detailed description |
| Zhou Q 2018 | 64/62 | 58.6±8.7/59.5±9.2 | Y | LBS 3 pills po bid; tamsulosin hydrochloride 0.2mg po qd; finasteride 5mg po qd | Tamsulosin hydrochloride 0.2mg po qd; finasteride 5mg po qd | 12 | ①②③④⑤⑥ | Detailed description |
| Niu M 2014 | 60/50 | 51-76/50-74 | Y | LBS 0.3g po bid; epristeride 5mg po qn | Epristeride 5mg po qn | 16 | ②④⑤⑥ | Detailed description |
| Peng CH 2012 | 28/27 | 50-78(65.61±6.95)/52-77(63.19±8.08) | Y | LBS 3 pills po bid; doxazosin mesylate 2mg po qd | Doxazosin mesylate 2mg po qn | 24 | ①②④⑤ | Detailed description |
| Wang SQ 2020 | 103/103 | 70.30±8.29/70.25±8.27 | Y | LBS 3 pills po bid; Finasteride 5mg po qd | Finasteride 5mg po qd | 12 | ①②③④⑤⑥ | Detailed description |
| Ji DL 2018 | 108/108 | 62.98±6.41/63.25±6.57 | Y | LBS 0.3g po bid; epristeride 5mg po qn | Epristeride 5mg po qn | 16 | ①②④⑤⑥ | Detailed description |
| Chen XS 2017 | 58/58 | 68.35±3.11/68.26±3.07 | Y | LBS 3 pills po bid; Tamsulosin hydrochloride 0.2mg po qd | Tamsulosin hydrochloride 0.2mg po qd | 12 | ①③④⑤⑥ | Detailed description |
| Xue P 2017 | 58/58 | 76.58±6.32/75.87±5.79 | Y | LBS 3 pills po bid; tamsulosin tablet 0.2mg po qd | Tamsulosin tablet 0.2mg po qd | 12 | ①②③④⑥ | Detailed description |
| Zhang YJ 2016 | 46/46 | 69.8±12.2/68.24±12.5 | Y | LBS 3 pills po bid; tamsulosin tablet 0.2mg po qd | Tamsulosin tablet 0.2mg po qd | 12 | ①②③ | Detailed description |
| Lu HZ 2020 | 55/55 | 68.8±3.5/69.2±3.2 | Y | LBS 3 pills po bid; doxazosin 1 pills po qd | Doxazosin 1 pills po qd | 24 | ①②③④⑤⑥ | Detailed description |
| Tao XF 2012 | 32/32 | 50-80 | Y | LBS 1.8g po qd; finasteride 5mg po qd | Finasteride 5mg po qd | 12 | ②④⑥ | NR |
| Yuan MD 2017 | 47/47 | 68.29±8.57/67.85±8.46 | Y | LBS 3 pills po bid; diethylstilbestrol 1mg po qd; finasteride 5mg po tid | Diethylstilbestrol 1mg po qd; finasteride 5mg po tid | 12 | ①②④⑤⑥ | Detailed description |
| Song CS 2016 | 115/113 | 65.7±7.7/64.8±7.9 | Y | LBS 3 pills po bid; doxazosin 1 pills po qd | Doxazosin 1 pills po qd | 52 | ①②③④⑤⑥ | NR |
| Chang DG 2015 | 28/27 | 65.61±6.95/63.19±8.08 | Y | LBS 3 pills po bid; doxazosin 2mg po qn | Doxazosin 2mg po qn | 24 | ①②③④ | Detailed description |
| Zhang DX 2022 | 53/53 | 57-70(63.50±6.50)/58-71(64.50±6.50) | Y | LBS 3 pills po bid; tamsulosin hydrochloride 0.2mg po qd | Tamsulosin hydrochloride 0.2mg po qd | 4 | ①②③④⑥ | Detailed description |
| Wang Y 2006 | 46/45 | ＞50 | Y | LWDH 1 pill po bid; Epristeride 5mg po bid | Epristeride 5mg po bid | 8 | ②③④⑤⑥ | NR |
| Wei LY 2016 | 62/62 | 60-86(70.3±3.5)/61-85(70.1±3.2) | Y | LWDH 10g po tid; tamsulosin 0.2mg po qd; finasteride 5mg po qd | Tamsulosin 0.2mg po qd; finasteride 5mg po qd | 12 | ①②③④⑤⑥ | Detailed description |
| Zhu JH 2023 | 35/35 | 61.15±10.83/60.11±9.86 | Y | LZ 4 pills po tid; tamsulosin hydrochloride 0.2mg po qd | Tamsulosin hydrochloride 0.2mg po qd | 8 | ①④⑥ | Detailed description |
| Wang CL 2022 | 42/42 | 71.2±8.7/70.9±8.7 | Y | LZ 4 pills po tid; tamsulosin hydrochloride 0.2mg po qn | Tamsulosin hydrochloride 0.2mg po qn | 4 | ①② | NR |
| Li HQ 2022 | 160/160 | 59.17±8.03/58.93±8.47 | Y | LZ 4 pills po tid; tamsulosin hydrochloride 0.2mg po qd | Tamsulosin hydrochloride 0.2mg po qd | 12 | ①②③④⑥ | NR |
| Zhao MJ 2023 | 150/150 | 58.65±7.94/58.67±7.99 | Y | LZ 4 pills po tid; epristeride 5mg po bid | Epristeride 5mg po bid | 6 | ①②③④⑥ | Detailed description |
| Li ZS 2012 | 67/66 | 51-77(65.2±3.8)/53-78(64.6±4.1) | Y | PLA 4 pills po tid; tamsulosin 0.2mg po qn | Tamsulosin 0.2mg po qn | 4 | ②④⑤⑥ | Detailed description |
| Su M 2012 | 32/32 | 52-76(61.6±4.2)/50-76(62.5±4.1) | Y | PLA 4 pills po tid; terazosin hydrochloride 2mg po qn | Terazosin hydrochloride 2mg po qn | 8 | ②④⑤⑥ | Detailed description |
| Kong XH 2020 | 44/44 | 50-76(64.73±3.52)/51-75(64.85±3.27) | Y | PLA 4 pills po tid; naftopidil 25 mg po qd | Naftopidil 25 mg po qd | 4 | ①②③④⑥ | Detailed description |
| Ye YS 1993 | 41/29 | 50-84/51-86 | Y | PLA 4 pills po tid; prazonsin 1mg po tid | Prazonsin 1mg po tid | 2 | ① | NR |
| Gao JR 2014 | 20/16 | 45-62 (53±3.1)/41-61 (57±2.3) | Y | PLA 5 pills po tid; finasteride 5mg po qd | Finasteride 5mg po qd | 2 | ②④⑤⑥ | NR |
| Qi YJ 2011 | 65/63 | 51-79 (65.6±7.4)/53-77(63.7±6.8) | Y | QLBX 6 pills po tid; finasteride 5mg po qd | Finasteride 5mg po qd | 12 | ②③④⑤⑥ | Detailed description |
| Li W 2017 | 150/150 | 52-80(62.26±6.42)/50-78(62.08±6.57) | Y | QLBX 6 pills po tid; prostat tablet 1 pills po bid | Prostat tablet 1 pills po bid | 12 | ①②③④⑤⑥ | Detailed description |
| Zhang TJ 2015 | 40/38 | 57.1±8.4/56.1±7.9 | Y | QLBX 6 pills po tid; finasteride 5 mg po qd | Finasteride 5 mg po qd | 12 | ②③④⑤⑥ | NR |
| Tan C 2021 | 43/43 | 54.87±6.22/56.09±7.15 | Y | QLBX 6 pills po tid; finasteride 5 mg po qd | Finasteride 5 mg po qd | 12 | ②④⑤⑥ | NR |
| Chen H 2016 | 27/27 | 50-80(67.37±4.28) | Y | QLS 9g po tid; tamsulosin 0.2mg po qn | Tamsulosin 0.2mg po qn | 52 | ① | NR |
| Zhang TB 2020 | 50/50 | 42-74(65.51±4.55)/42-75(65.65±4.61) | Y | QLS 9g po tid; doxazosin mesylate 2mg po qd | Doxazosin mesylate 2mg po qd | 12 | ①②③④⑤⑥ | NR |
| Chang JK 2020 | 40/40 | 53-74(63.63±4.90)/53-73(63.51±4.84) | Y | QLS 9g po tid; silodosin 4mg po bid | Silodosin 4mg po bid | 4 | ①②④⑤⑥ | NR |
| Mai HL 2023 | 43/43 | 53-74(63.17±2.52)/53-72(62.34±2.25) | Y | QLST 3 pills po tid; finasteride 5mg po qd | Finasteride 5mg po qd | 12 | ①②④⑤⑥ | NR |
| Man YP 2017 | 45/45 | 55-80(67.48±1.35)/56-80(67.44±1.37) | Y | QLST 3 pills po tid; epristeride 5mg po bid | Epristeride 5mg po bid | 12 | ①②③④⑤⑥ | NR |
| Ma ZF 2009 | 82/80 | 46-85(62.7±21.8)/44-89(63.1±23.4) | Y | QLST 3 pills po tid; doxazosin 4mg po qd | Doxazosin 4mg po qd | 8 | ②④ | Detailed description |
| Du X 2016 | 30/27 | 56-67(59.0±3.6)/56-65(58.8±3.4) | Y | QLST 3 pills po tid; finasteride 5mg po qn | Finasteride 5mg po qn | 12 | ②④⑤⑥ | NR |
| Yin ZK 2018 | 53/53 | 49-80(66.4±3.0)/51-81(67.6±2.2) | Y | QLST 3 pills po tid; finasteride 5mg po qd | Finasteride 5mg po qn | 12 | ①②④⑤⑥ | NR |
| Zhang F 2017 | 58/58 | 50-74(62.73±8.91)/50-74(62.73±8.91) | Y | QLST 3 pills po tid; tamsulosin hydrochloride 0.2mg po qn | Tamsulosin hydrochloride 0.2mg po qn | 12 | ①②④⑤⑥ | NR |
| Xuan SQ 2012 | 40/40 | 52-78(63.4±13.8)/53-85(64.1±15.2) | Y | QLST 3 pills po tid; tamsulosin hydrochloride 0.2mg po qd | Tamsulosin hydrochloride 0.2mg po qd | 4 | ①②③④⑤⑥ | NR |
| Wang J 2014 | 40/40 | 57-79 | Y | QLST 3 pills po tid; doxazosin 4mg po qn | Doxazosin 4mg po qn | 12 | ②④⑤⑥ | NR |
| Yu HC 2016 | 48/48 | 45-82(60.21±8.58)/45-83(60.40±8.26) | Y | QLST 3 pills po tid; tamsulosin hydrochloride 0.2mg po qd | Tamsulosin hydrochloride 0.2mg po qd | 12 | ①②④⑤⑥ | NR |
| Wang GR 2018 | 61/61 | 55-72(64.3±2.5)/56-75(63.9±2.4) | Y | QLST 3 pills po tid; tamsulosin 0.2mg po qd | Tamsulosin 0.2mg po qd | 12 | ①②④⑤⑥ | NR |
| Li H 2015 | 60/54 | 51-67(59.0±8.6)/50-68(58.8±9.4) | Y | QLST 3 pills po tid; tamsulosin hydrochloride 0.2mg po qn | Tamsulosin hydrochloride 0.2mg po qn | 12 | ①②④⑤⑥ | Detailed description |
| Zhu HG 2011 | 28/28 | 52-83(64.7)/52-79(64.1) | Y | QLST 3 pills po tid; tamsulosin hydrochloride 0.2mg po qn | Tamsulosin hydrochloride 0.2mg po qn | 8 | ②④⑤⑥ | Detailed description |
| Liang JM 2018 | 60/60 | 62.25±2.11/62.30±2.14 | Y | QLST 3 pills po tid; finasteride 5mg po qd | Finasteride 5mg po qd | 8 | ①②④⑤⑥ | Detailed description |
| Tong P 2020 | 40/40 | 53-71(62.32±5.01)/51-69(62.23±4.51) | Y | QLST 3 pills po tid; tamsulosin hydrochloride 0.2mg po qd | Tamsulosin hydrochloride 0.2mg po qd | 12 | ②⑤⑥ | Detailed description |
| Gao W 2022 | 45/45 | 53-78(65.03±3.87)/52-79(66.14±3.60) | Y | QLST 3 pills po tid; epristeride 5mg po bid | Epristeride 5mg po bid | 12 | ①②④⑤⑥ | Detailed description |
| Fu QY 2016 | 56/53 | 74.8±6.7/75.8±7.1 | Y | QLST 3 pills po tid; tamsulosin 0.2mg po qd; finasteride 5mg po qd | Tamsulosin 0.2mg po qd; finasteride 5mg po qd | 52 | ①②⑤⑥ | NR |
| Fu XJ 2015 | 74/73 | 52-87(74.1±10.8)/52-85(73.3±11.2) | Y | QLST 3 pills po tid; phenoxybenzamine hydrochloride 20mg po bid | Phenoxybenzamine hydrochloride 20mg po bid | 4 | ②④⑤ | Detailed description |
| Li WQ 2020 | 64/64 | 58-72(63.4±4.8)/57-72(63.7±4.2) | Y | QLST 3 pills po tid; finasteride 5mg po qd | Finasteride 5mg po qd | 8 | ①②④⑤⑥ | NR |
| Li ZH 2019 | 100/100 | 46-63(56.7±7.04)/48-65(56.9±6.71) | Y | QLST 3 pills po tid; tamsulosin hydrochloride 0.2mg po qn | Tamsulosin hydrochloride 0.2mg po qn | 12 | ①②③④⑤ | Detailed description |
| Hao C 2009 | 20/20 | 56-78 | Y | QLST 3 pills po tid; doxazosin 4mg po qd | Doxazosin 4mg po qd | 12 | ②④⑤⑥ | Detailed description |
| Li WM 2017 | 43/43 | 48-69(59.2±3.7)/48-70(59.9±3.9) | Y | QLST 3 pills po tid; finasteride 5mg po qd | Finasteride 5mg po qd | 12 | ①②④⑤⑥ | NR |
| Zhou SM 2023 | 50/48 | 42-78(58.47±6.85)/41-79(58.25±6.67) | Y | QLST 3 pills po tid; tamsulosin hydrochloride 0.2mg po qd | Tamsulosin hydrochloride 0.2mg po qd | 4 | ①②④⑤⑥ | NR |
| Wan T 2017 | 56/56 | 50-72(60.5±2.3)/51-70(60.2±2.1) | Y | QLST 3 pills po tid; tamsulosin hydrochloride 0.2mg po qn | Tamsulosin hydrochloride 0.2mg po qn | 12 | ①②④⑤⑥ | Detailed description |
| Wu XH 2019 | 35/35 | 64.02±5.32/63.52±5.36 | Y | QLST 1 pills po tid; finasteride 5mg po qd | Finasteride 5mg po qd | 8 | ①④⑤⑥ | Detailed description |
| Duan ZX 2020 | 40/40 | 35-82(70.08±10.05)/36-83(70.25±10.31) | Y | QLST 3 pills po tid; tamsulosin hydrochloride 0.2mg po qd | Tamsulosin hydrochloride 0.2mg po qd | 64 | ① | NR |
| Zhang JM 2020 | 25/25 | 57-80(69.59±4.82)/56-79(68.99±4.71) | Y | QLST 3 pills po tid; finasteride 5mg po qd | Finasteride 5mg po qd | 8 | ①④⑤⑥ | NR |
| Tian ZY 2018 | 45/45 | 61-80(71.08±5.54)/60-80(70.20±5.21) | Y | QLST 3 pills po tid; finasteride 5mg po qd | Finasteride 5mg po qd | 8 | ①②④⑤⑥ | NR |
| Kong LJ 2019 | 64/64 | 42-69(55.6±2.1)/45-68(55.5±2.2) | Y | QLST 3 pills po tid; tamsulosin hydrochloride 0.2mg po qn | Tamsulosin hydrochloride 0.2mg po qn | 12 | ①②④⑤⑥ | Detailed description |
| Li BL 2019 | 24/24 | 61-74(68.5±2.7)/60-73(67.2±2.6) | Y | QLST 3 pills po tid; finasteride 5mg po qd | Finasteride 5mg po qd | 8 | ①②④⑤⑥ | Detailed description |
| Zhang X 2022 | 58/57 | 57.26±6.83/55.46±6.66 | Y | QLST 3 pills po tid; tamsulosin hydrochloride 0.2mg po tid; | Tamsulosin hydrochloride 0.2mg po tid | 4 | ①②④⑤⑥ | Detailed description |
| Hu JS 2019 | 34/34 | 66-79(74.12±3.79)/65-80(74.13±3.62) | Y | QLST 3 pills po tid; finasteride 5mg po qd | Finasteride 5mg po qd | 8 | ②④⑥ | NR |
| Gao K 2016 | 30/30 | 53-84(73.2±10.3) | Y | QLST 3 pills po tid; phenoxybenzamine hydrochloride 20mg po bid | Phenoxybenzamine hydrochloride 20mg po bid | 4 | ②④⑤⑥ | NR |
| Shi B 2016 | 44/44 | 60-72(65.36±2.53)/62-70(65.34±2.51) | Y | QLST 3 pills po tid; finasteride 5mg po qd | Finasteride 5mg po qd | 8 | ①②④⑤⑥ | NR |
| Su HY 2015 | 48/54 | 67.3/68.5 | Y | QLTY 5 pills po tid; tamsulosin 0.2mg po qd | Tamsulosin 0.2mg po qd | 4 | ②④⑤⑥ | Detailed description |
| Geng CM 2014 | 38/38 | 67.3±9.8/68.5±8.7 | Y | QLTY 5 pills po tid; tamsulosin hydrochloride 0.2mg po qn | Tamsulosin hydrochloride 0.2mg po qn | 4 | ②④⑤⑥ | Detailed description |
| Liu CG 2010 | 42/36 | 53-80 | Y | QLTY 5 pills po tid; finasteride 5mg po qd | Finasteride 5mg po qd | 8 | ①②⑥ | NR |
| Gao Z 2011 | 33/30 | 51-84(71±7)/52-83(70±6) | Y | QLX 3g po tid; tamsulosin hydrochloride 0.2mg po qd | Tamsulosin hydrochloride 0.2mg po qd | 4 | ②④⑤⑥ | NR |
| Wang CX 2017 | 48/46 | 61-78(65.58±7.48)/61-80(66.12±6.87) | Y | QLX 2.5g po tid; finasteride 5mg po qd | Finasteride 5mg po qd | 12 | ①②④⑤⑥ | NR |
| Xu MK 2020 | 78/79 | 54-79(65.32±3.86)/56-80(66.14±4.12) | Y | QLX 2.5g po tid; doxazosin 2mg po qd for 2 weeks, and then 4mg po qd | Doxazosin 2mg po qd for 2 weeks, and then 4mg po qd | 12 | ①②③④⑤⑥ | Detailed description |
| Wu HJ 2019 | 43/43 | 53-78(65.59±4.58)/54-79(65.54±5.67) | Y | QLX 2.5g po tid; tamsulosin hydrochloride 0.2mg po qd | Tamsulosin hydrochloride 0.2mg po qd | 12 | ①②④⑤⑥ | Detailed description |
| Zhao XS 2020 | 41/41 | 54-76(65.62±1.75)/54-75(65.24±1.36) | Y | QLX 3g po tid; terazosin 2mg po qd | Terazosin 2mg po qd | 4 | ①②③④⑤⑥ | NR |
| F. Hizli 2007 | 20/20 | 60.2±6.3/58.9±5.7 | Y | SR 320 mg po qd; tamsulosin 0.4 mg po qd | Tamsulosin 0.4 mg po qd | 24 | ②③④⑤⑥ | Detailed description |
| Y. W. Ryu 2015 | 50/53 | 62.5±1.21/63.4±1.44 | Y | SR 320 mg po qd; tamsulosin 0.2 mg po qd | Tamsulosin 0.2 mg po qd | 52 | ②③④⑤⑥ | Detailed description |
| A. Argirovic 2013 | 81/87 | 65.9±7.4/56.8±7.7 | Y | SR 320 mg po qd; tamsulosin 0.4 mg po qd | Tamsulosin 0.4 mg po qd | 24 | ②③④⑤⑥ | Detailed description |
| Fabrizio Di Maida 2019 | 76/72 | 66.4±1.20/65.7±1.42 | Y | SR 320 mg po qd; tamsulosin 0.4 mg po qd | Tamsulosin 0.4 mg po qd | 52 | ②③④⑤⑥ | Detailed description |
| Xia HQ 2013 | 45/42 | 53-79(62.3±4.7)/55-81(63.6±5.1) | Y | SPE 160 mg po bid; tamsulosin 0.2mg po qn | Tamsulosin 0.2mg po qd | 8 | ②④⑤⑥ | Detailed description |
| Zhou XP 2014 | 46/46 | 55-75 | Y | SPE 160mg po bid; tamsulosin hydrochloride 0.2mg po qn | Tamsulosin hydrochloride 0.2mg po qn | 12 | ②③④⑤⑥ | NR |
| Zhou PG 2021 | 30/30 | 55.8±5.1/56.0±5.5 | Y | SPE 160mg po bid; tamsulosin hydrochloride 0.2mg po qn | Tamsulosin hydrochloride 0.2mg po qn | 12 | ②③④⑤⑥ | Detailed description |
| Jiang F 2019 | 120/120 | 71.86±7.59/72.94±8.52 | Y | WLT 3 pills po bid; doxazosin mesylate 4mg po qd; LBS 3 pills po bid | Doxazosin mesylate 4mg po qd; LBS 3 pills po bid | 4 | ①②③⑤ | NR |
| Zhang XS 2012 | 48/52 | 55-84(66.7±5.5)/56-81(65.8±5.3) | Y | WLT 3 pills po bid; tamsulosin 0.2mg po qd; finasteride 5mg po qd | Tamsulosin 0.2mg po qd; finasteride 5mg po qd | 12 | ①②③④⑤ | NR |
| Gu DL 2021 | 41/41 | 51-78(64.57±1.29)/51-79(64.88±1.51) | Y | WLT 3 pills po bid; tamsulosin 0.2mg po qd | Tamsulosin 0.2mg po qd | 12 | ①②④⑤⑥ | NR |
| Luo QY 2020 | 40/40 | 52-79(65.2±4.7)/49-76(64.7±5.0) | Y | XLQ 3 pills po tid; tamsulosin 0.2mg po qd | Tamsulosin 0.2mg po qd | 12 | ①②④⑤⑥ | Detailed description |
| Guo TB 2021 | 30/30 | 58-76(68.10±6.09)/55-74(65.03±6.12) | Y | XLQ 3 pills po tid; tamsulosin 0.2mg po qd | Tamsulosin 0.2mg po qd; finasteride 5mg po qd | 12 | ①②⑤⑥ | Detailed description |
| Yang Z 2019 | 50/50 | 58-76(68.10±6.09)/57-74(68.03±6.15) | Y | XLQ 3 pills po tid; finasteride 5mg po qd | Finasteride 5mg po qd | 12 | ①②③④⑤⑥ | NR |
| Xiang Y 2012 | 56/42 | 50-77/52-72 | Y | ZGLS 2 pills po tid; terazosin 2mg po qn | Terazosin 2mg po qn | 8 | ②④⑥ | Detailed description |
| Liu JT 2009 | 42/42 | 56-83 | Y | ZGLS 2 pills po tid; tamsulosin hydrochloride 0.2mg po qd | Tamsulosin hydrochloride 0.2mg po qd | 12 | ②④⑥ | NR |
| Chen MX 2008 | 51/30 | 50-80 | Y | ZGLS 2 pills po tid; terazosin 2mg po qd | Terazosin 2mg po qd | 8 | ②③⑤⑥ | Detailed description |
| Li Z 2009 | 70/70 | 60.6±7.5/60.1±8.2 | Y | ZGLS 2 pills po tid; naftopidil 25 mg po qd | Naftopidil 25 mg po qd | 8 | ②③④⑤⑥ | NR |

Note: E/C, experimental group/control group; NR, Not Reported; Y, Yes; WM, Western medicine; GZFL, Guizhi Fuling capsule; HE, Huange capsule; JGSQ, Jingui Shenqi pill; LBS, Longbishu capsule; LWDH, Liuwei Dihuang pill; LZ, Lingze tablet; PLA, Pulean tablet; QLBX, Qianlie Beixi capsule; QLS, Qianlieshu pill; QLST, Qianlie Shutong capsule; QLTY, Qianlie Tongyu capsule; QLX, Qianliexin capsule; SPE, Saw Palmetto Extract capsule; SR, Serenoa repens; WLT, Wenglitong capsule; XLQ, Xialiqi capsule; ZGLS, Zegui Longshuang Capsule; ①, Clinical effective rate; ②, International Prostate Symptom Score; ③, Qol score; ④, Maximum flow rate of urine; ⑤, Prostate volume; ⑥, Postvoid residual urine.

# File S6: Quality assessment of the included studies.

## Summary of RoB2 assessment.


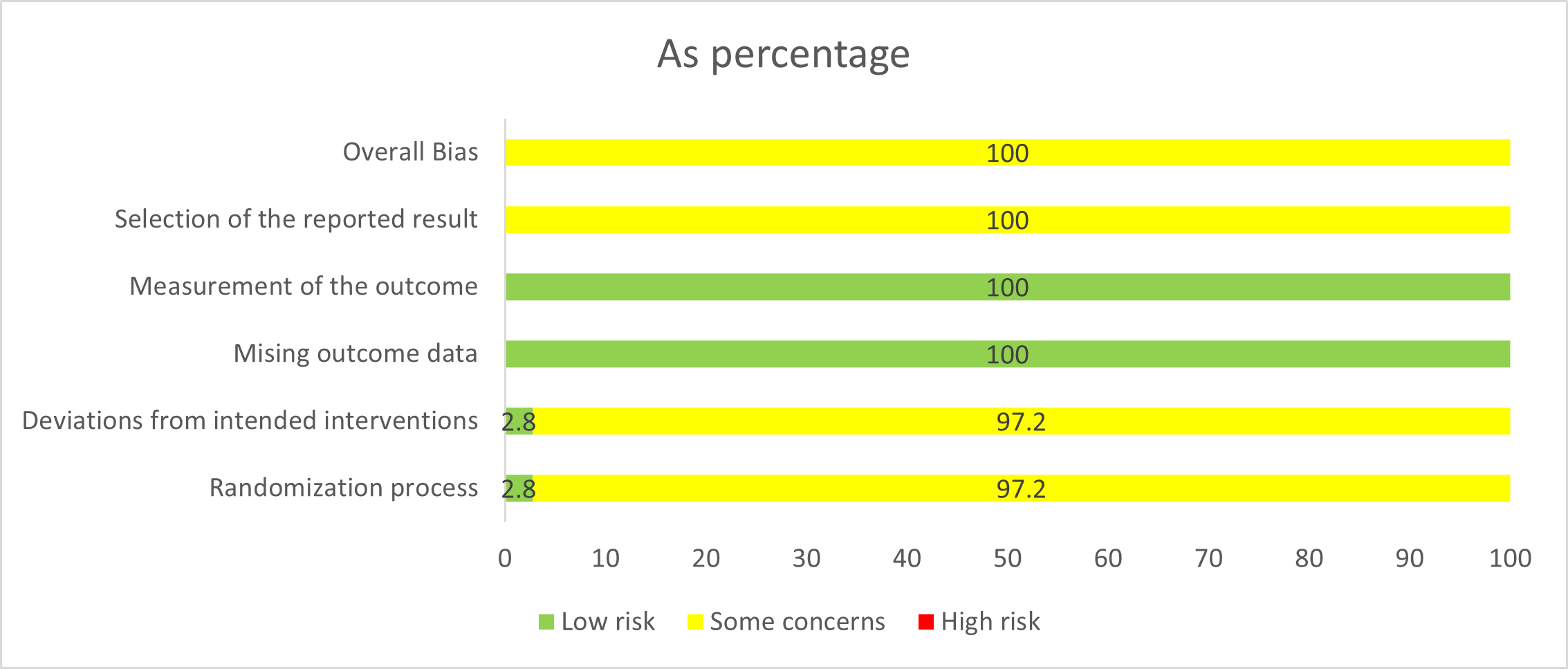


## RoB2 assessment of each included study.


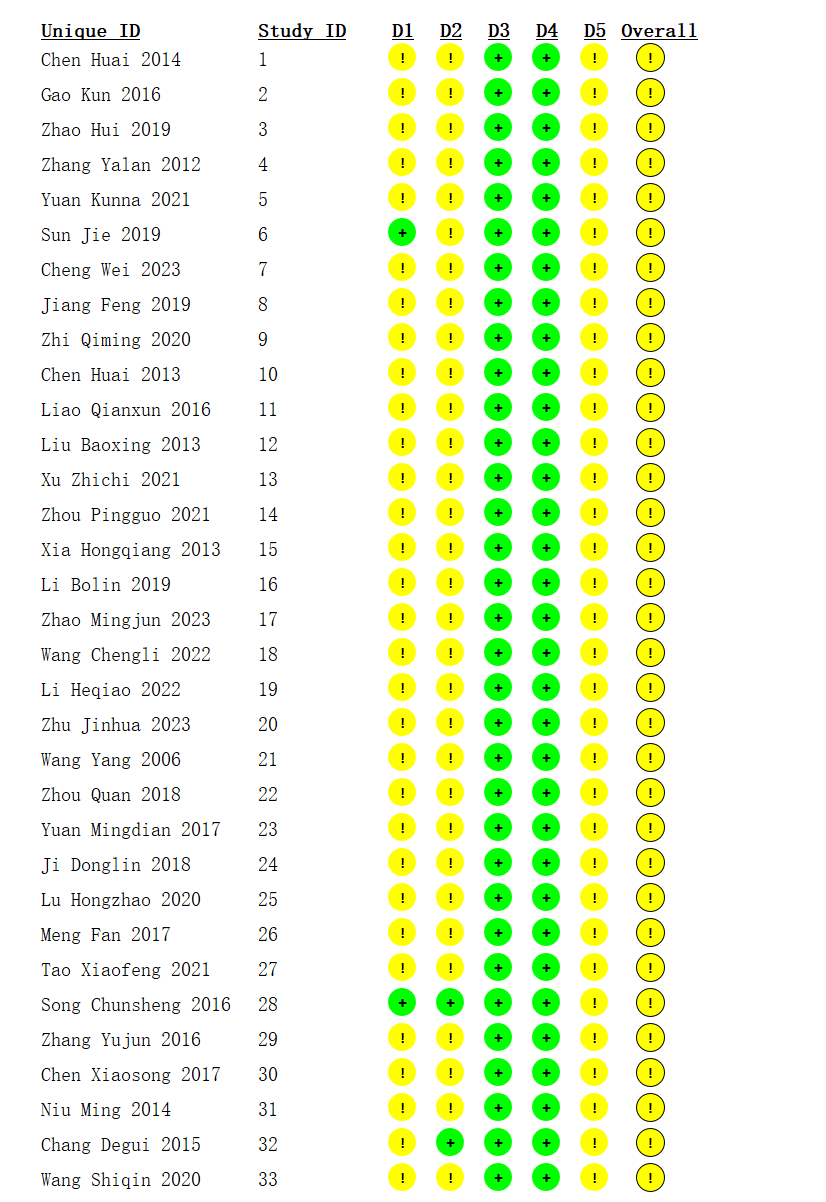


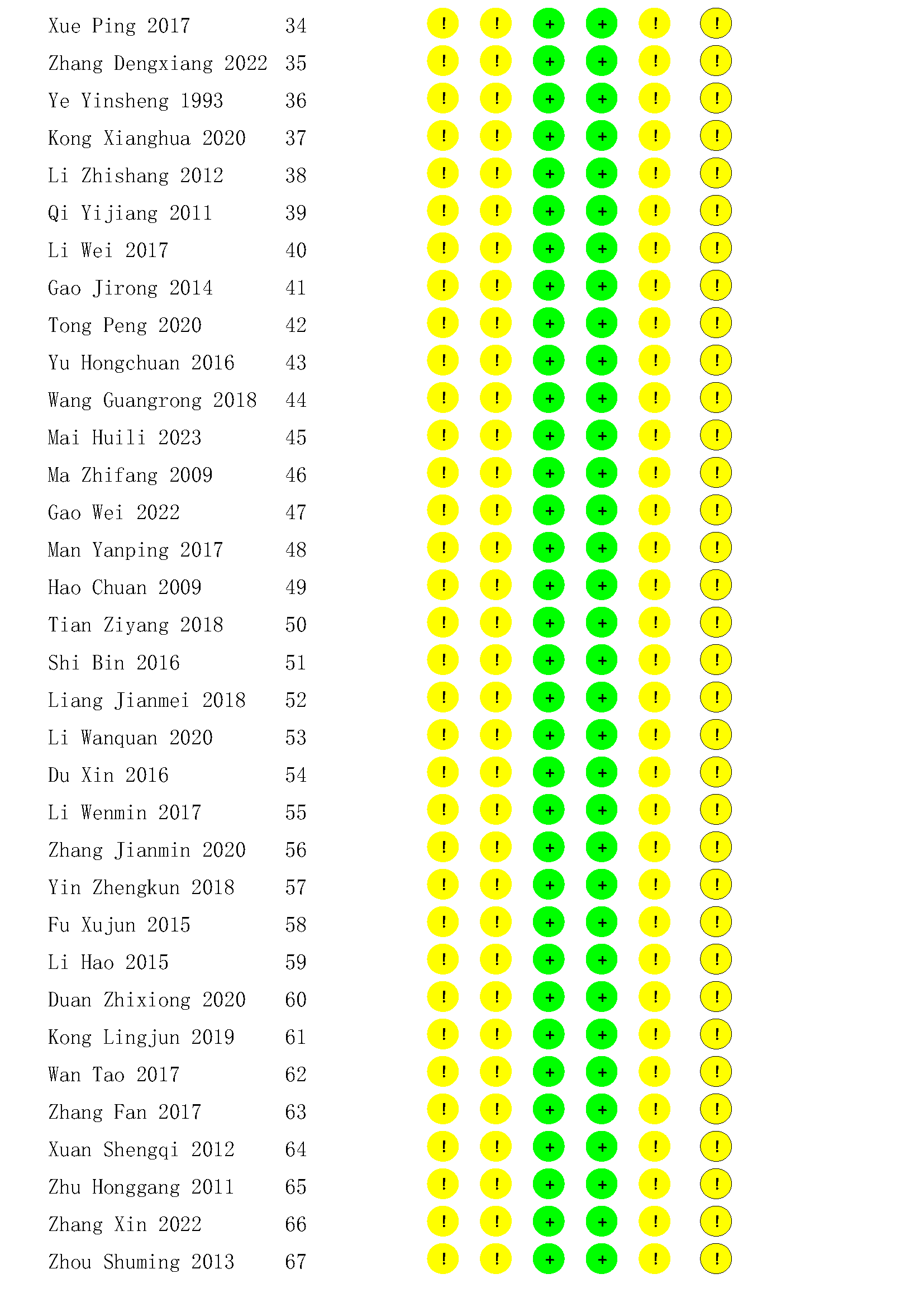


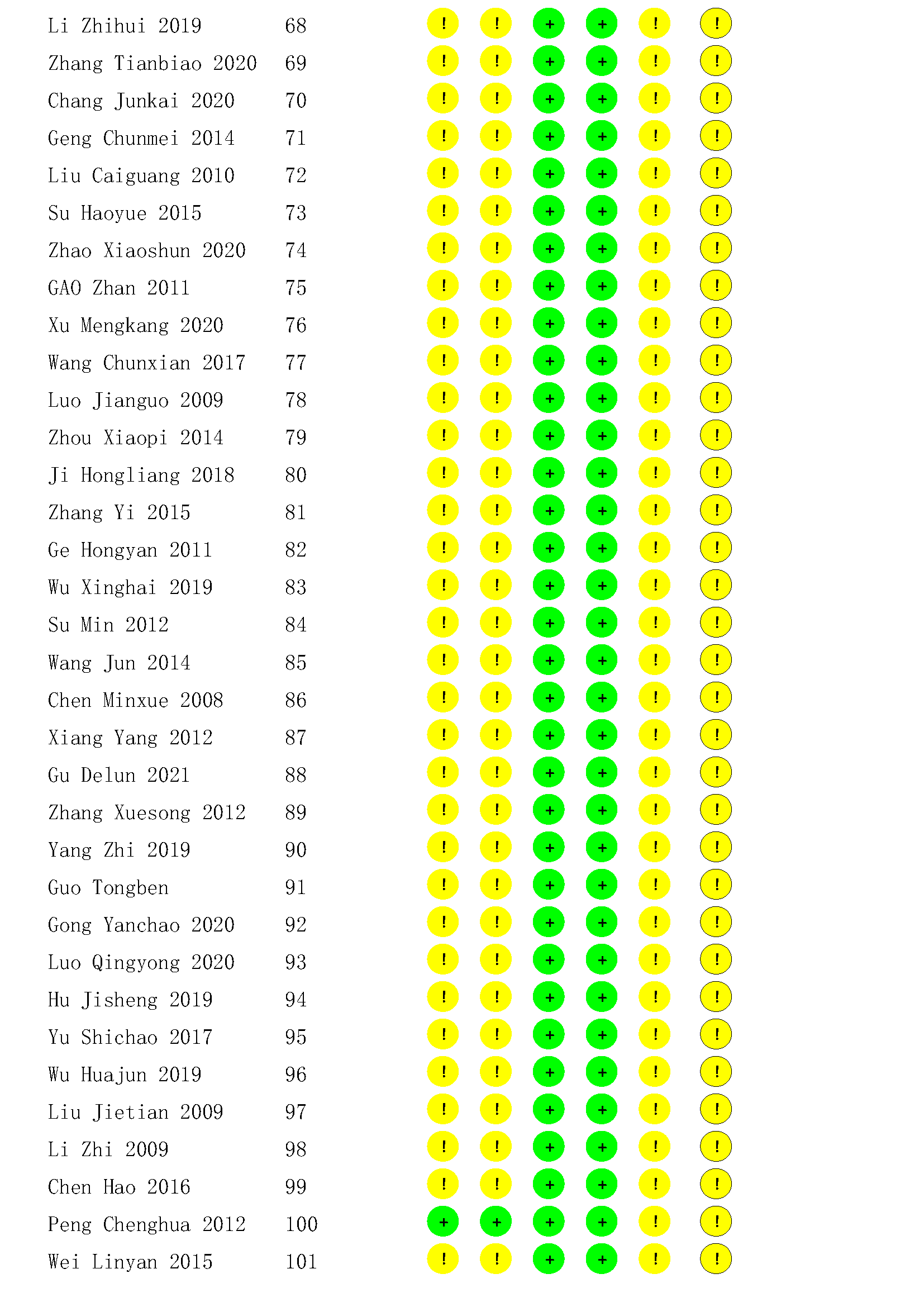


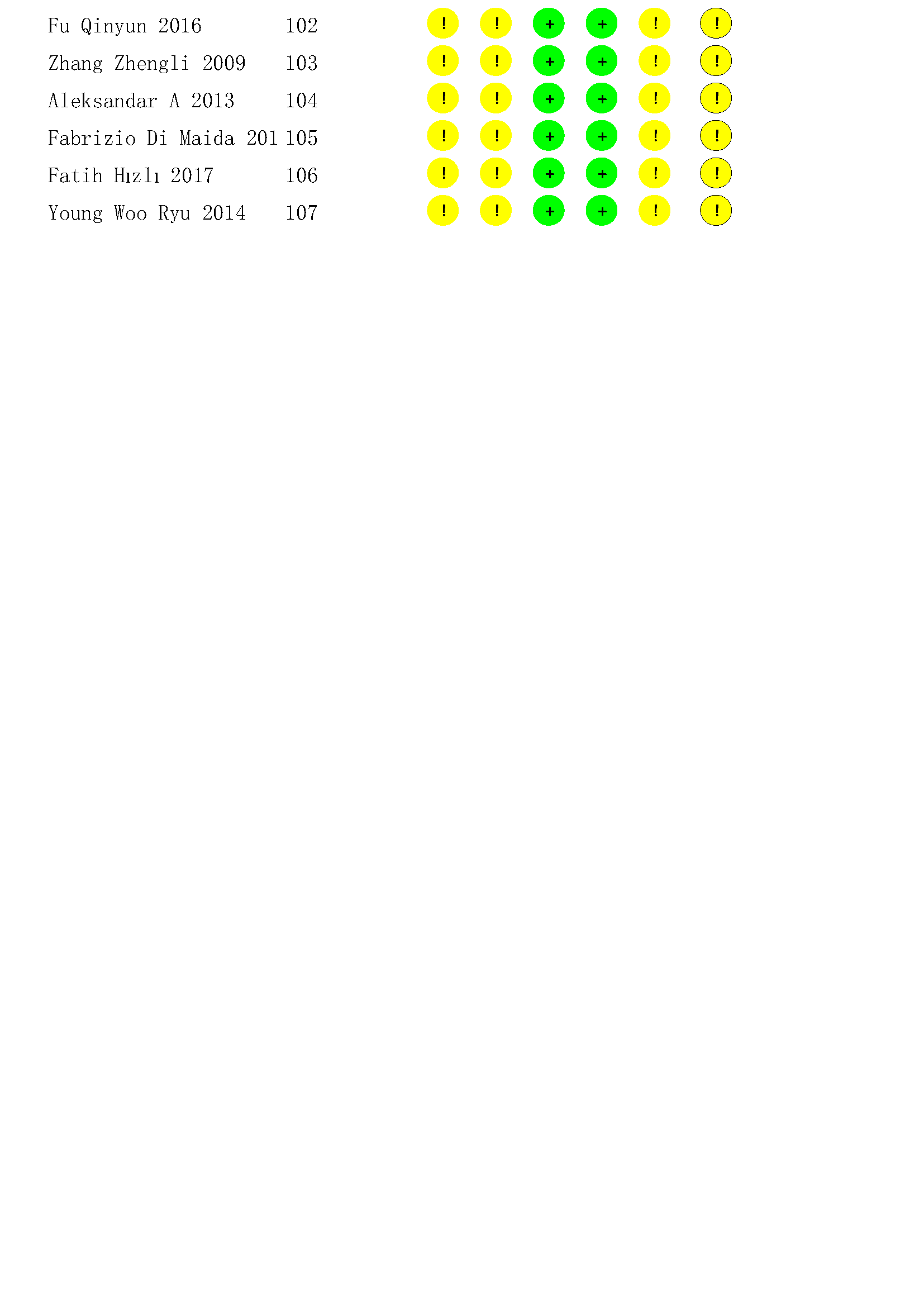


## NOS assessment of each included study.


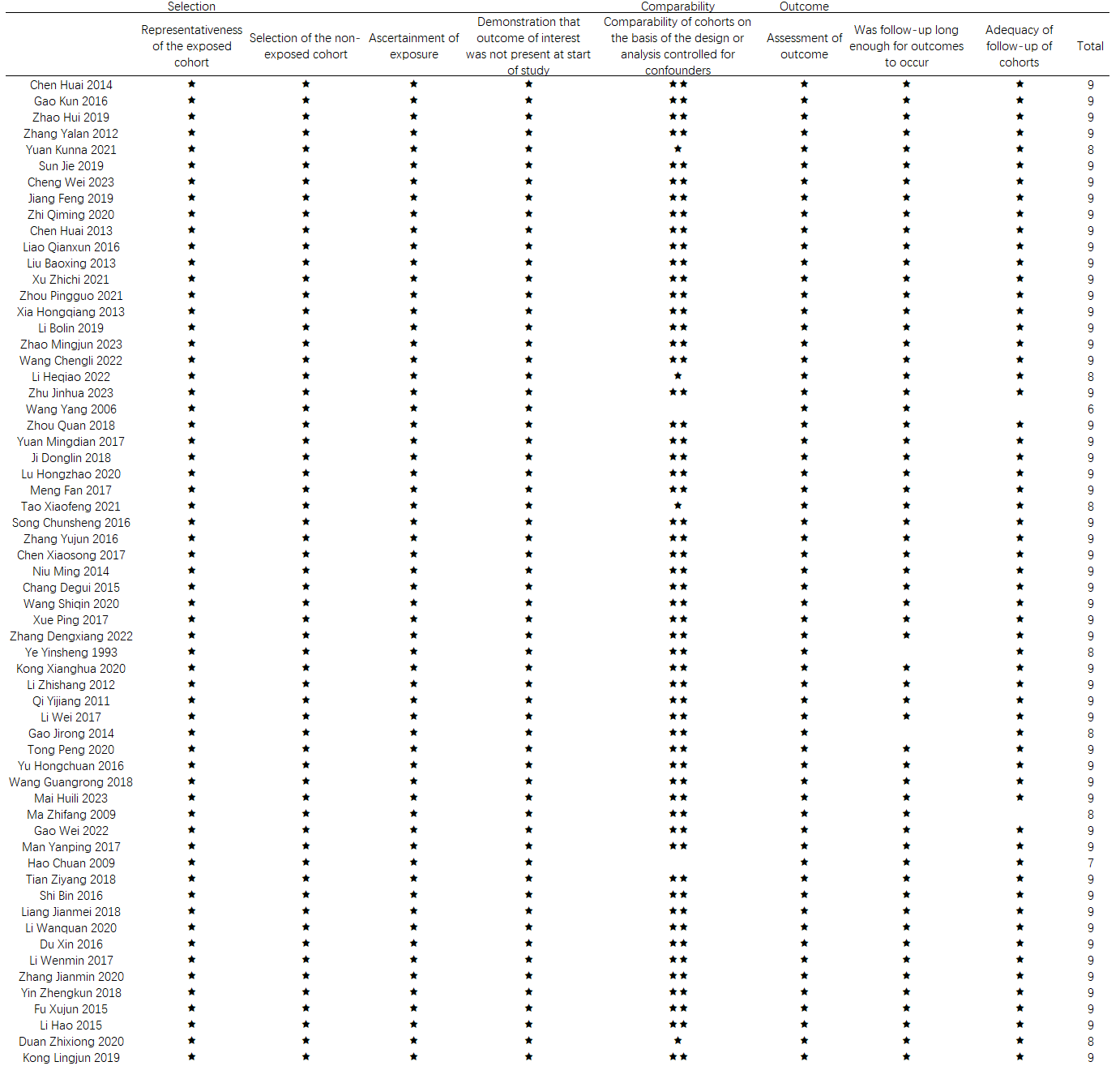


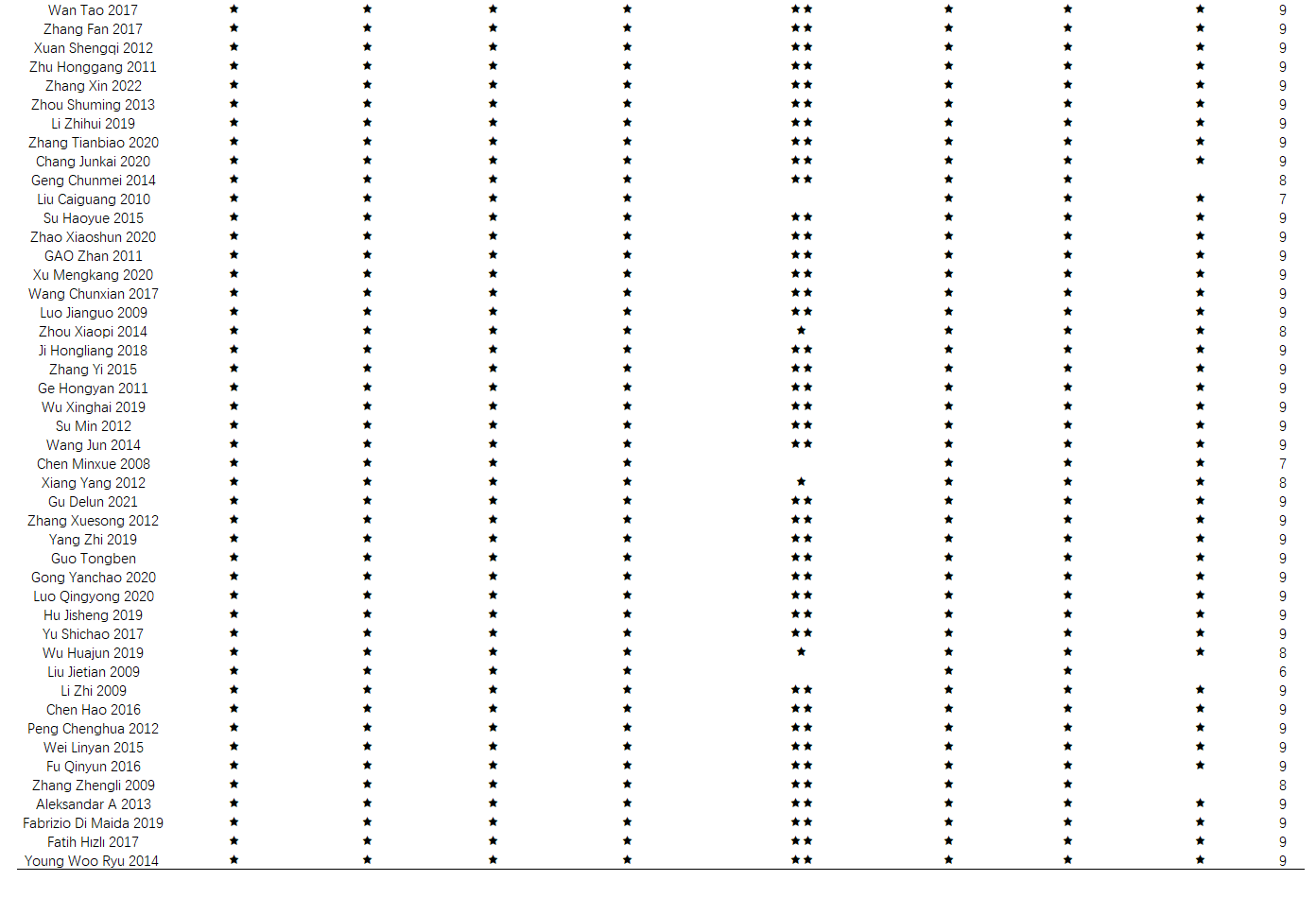


# File S7: Brooks-Gelman-Rubin plots.

## Brooks-Gelman-Rubin plots for clinical effective rate.


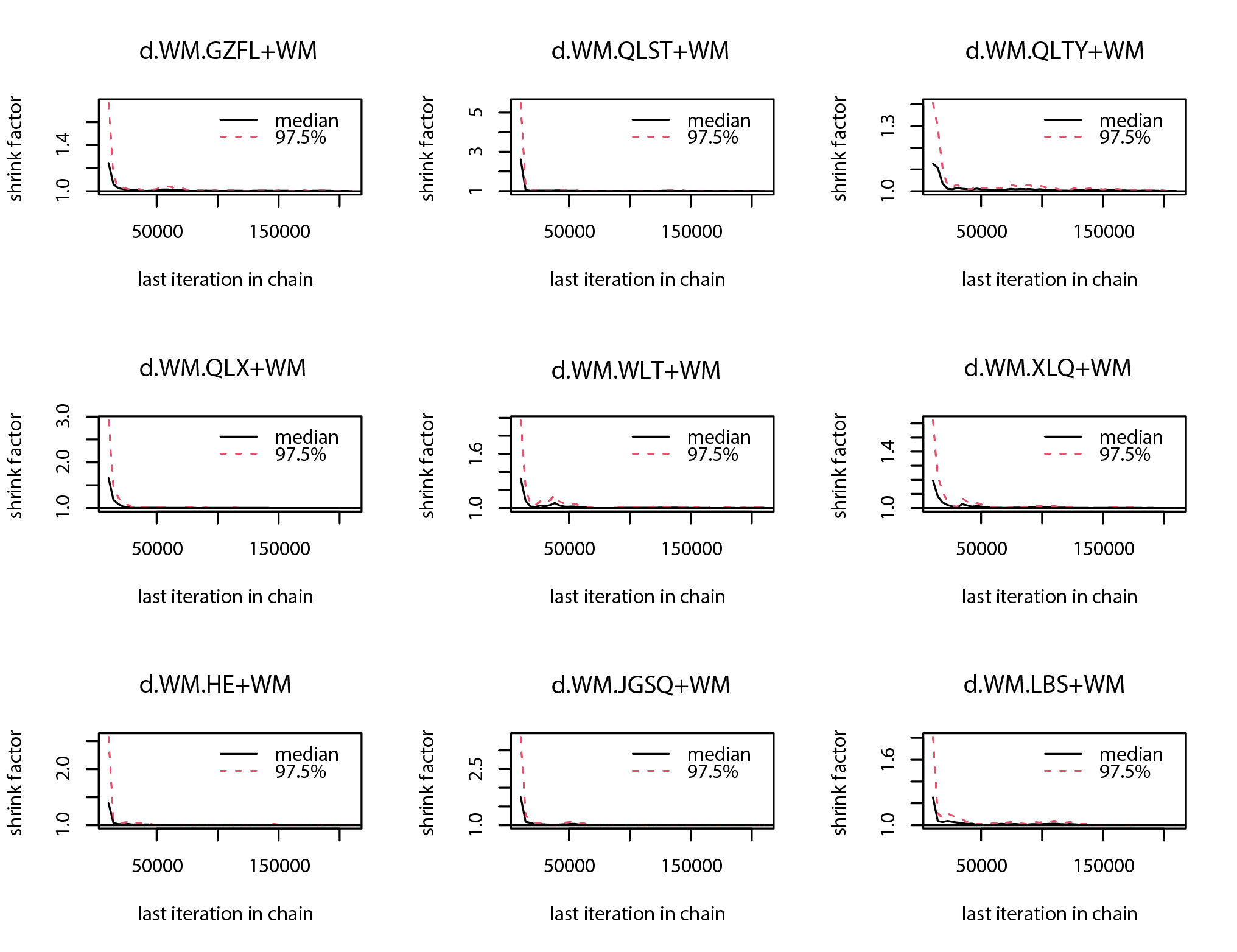


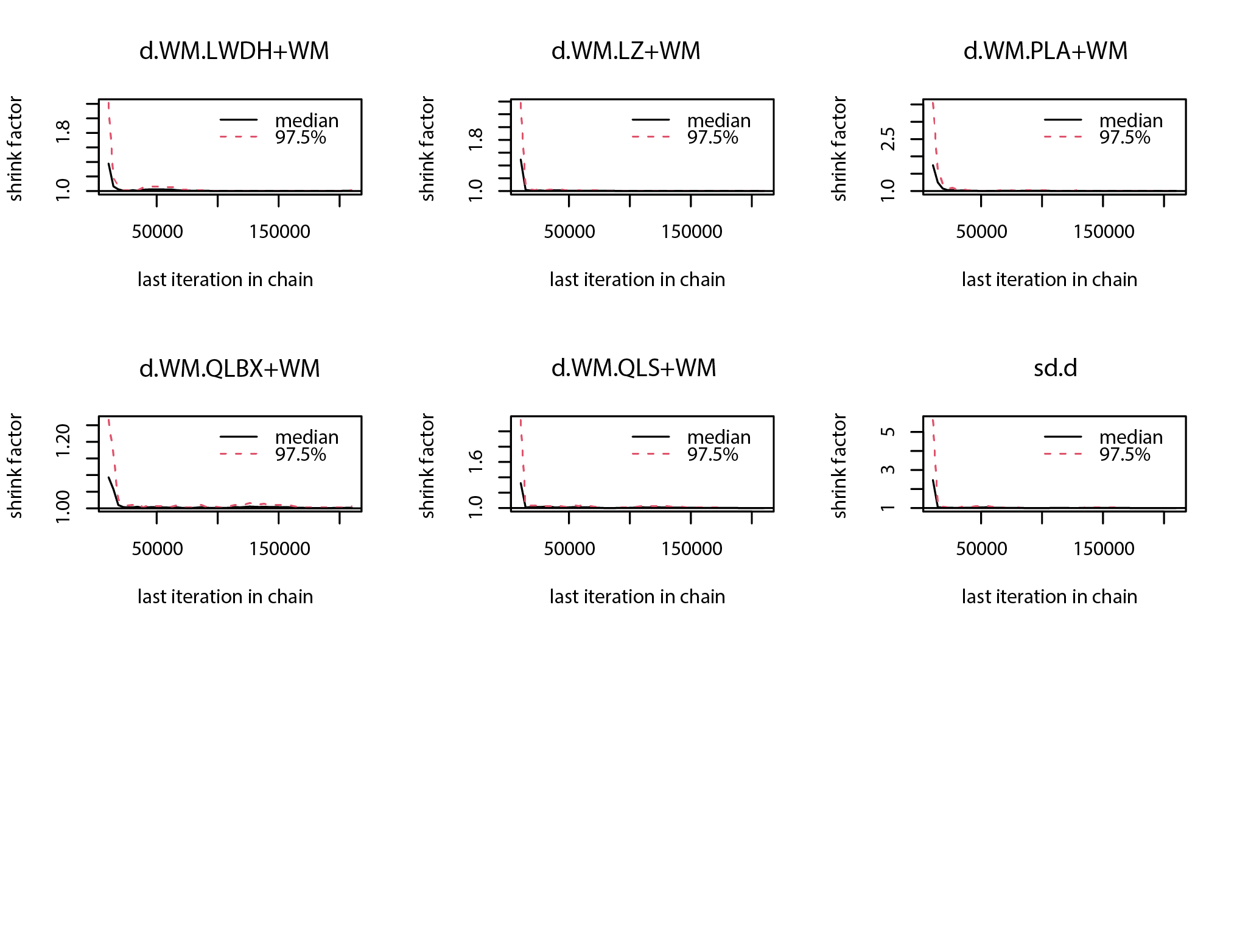


## Brooks-Gelman-Rubin plots for IPSS.

**
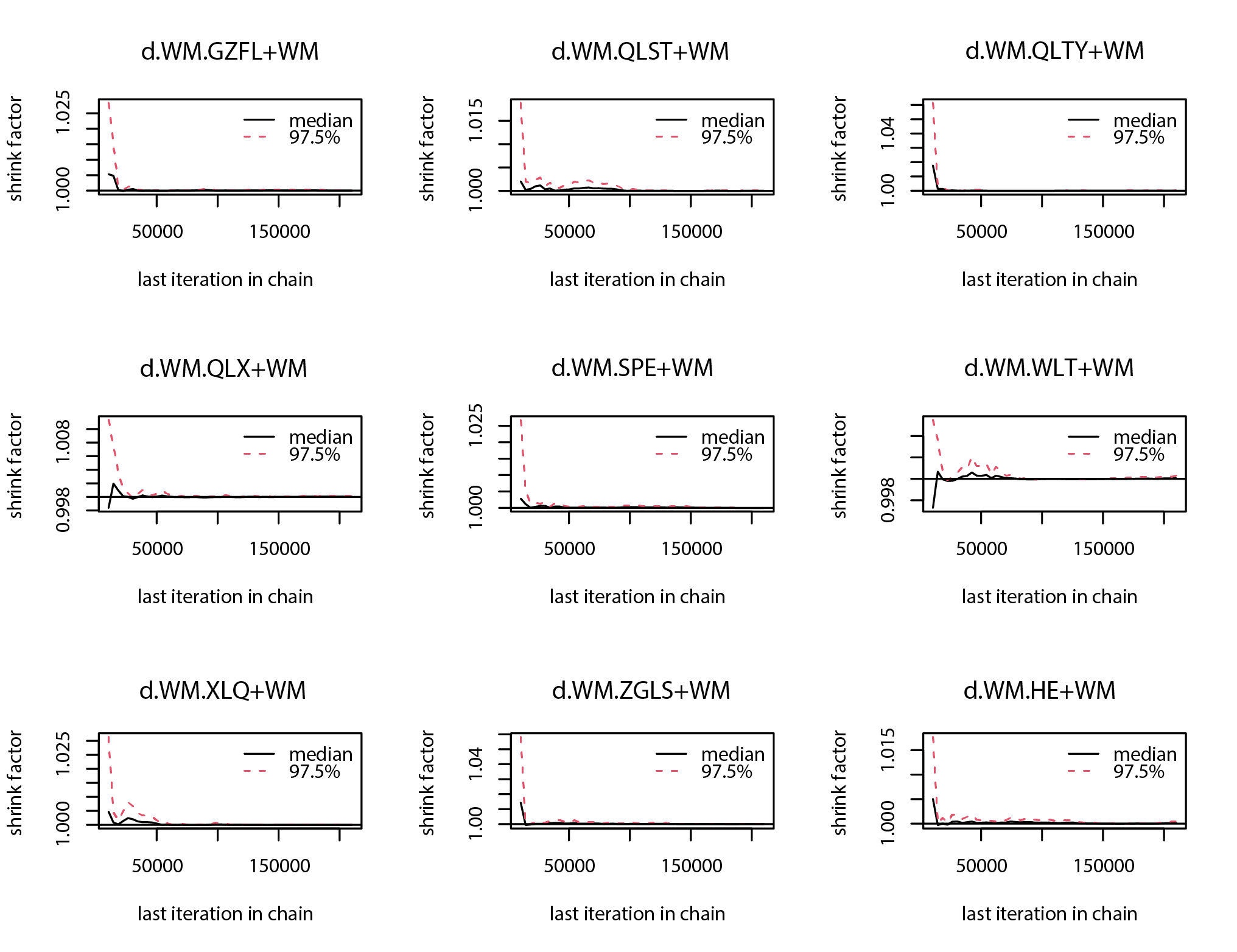
**

**
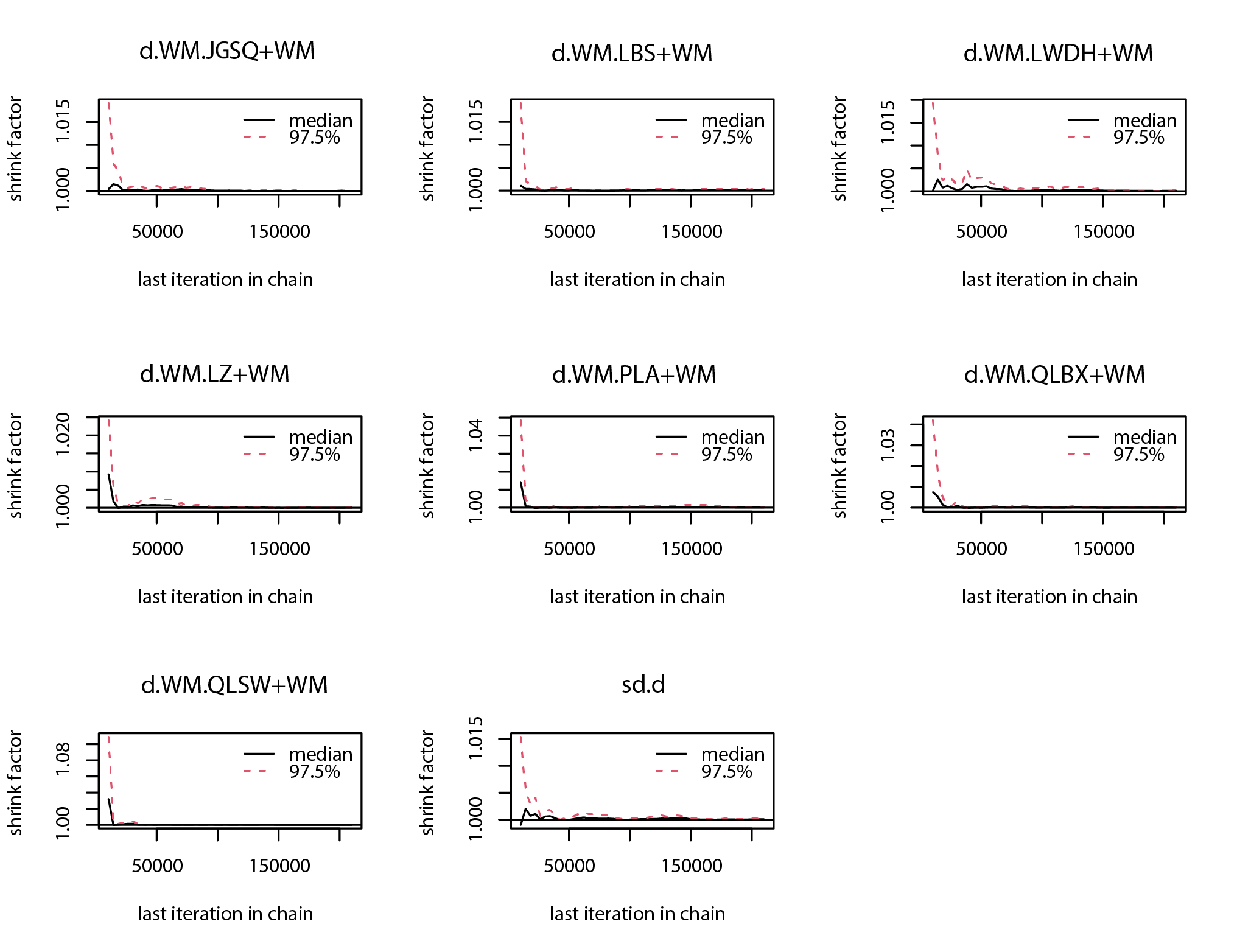
**

## Brooks-Gelman-Rubin plots for QoL score.


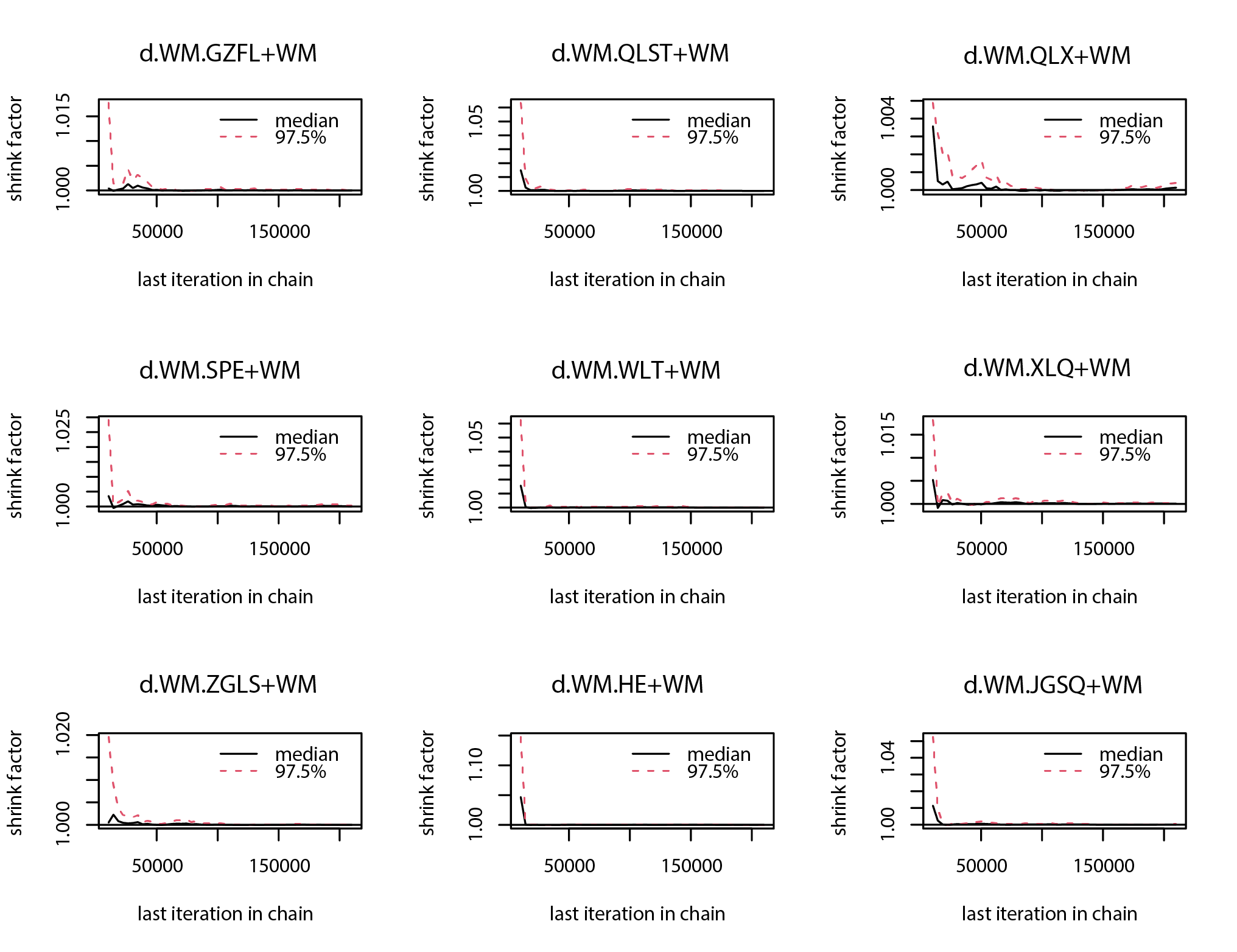


**
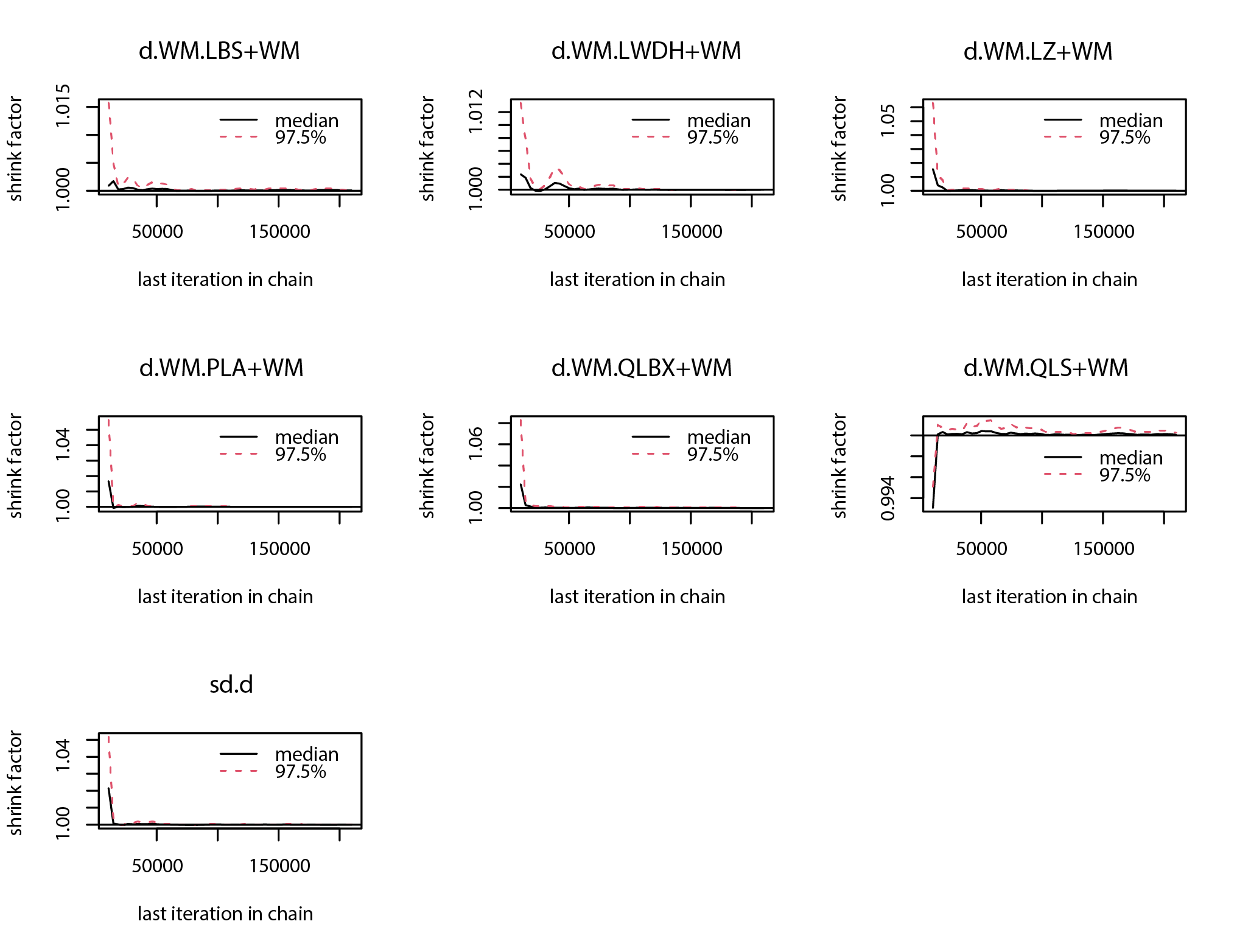
**

## Brooks-Gelman-Rubin plots for maximum flow rate.

**
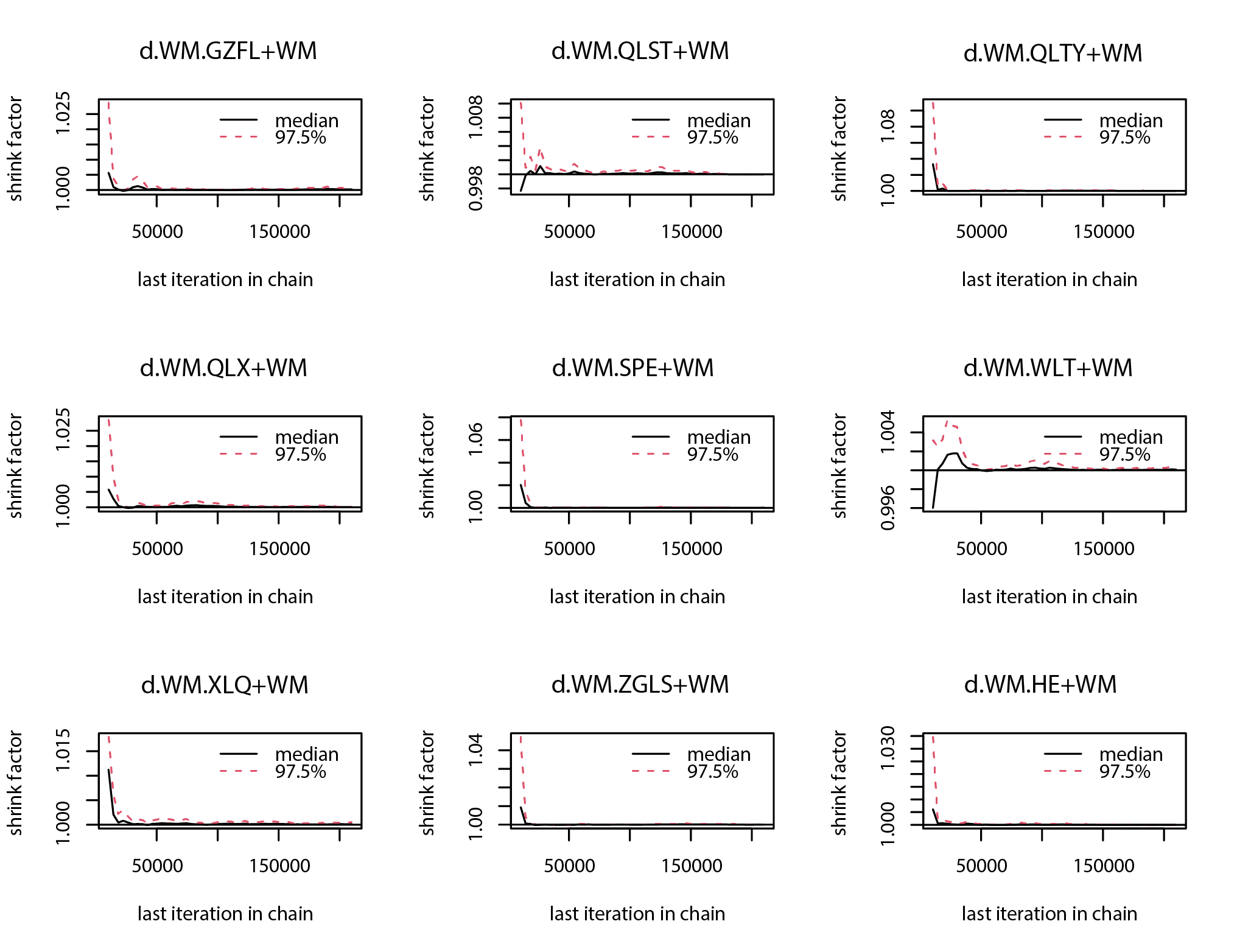
**

**
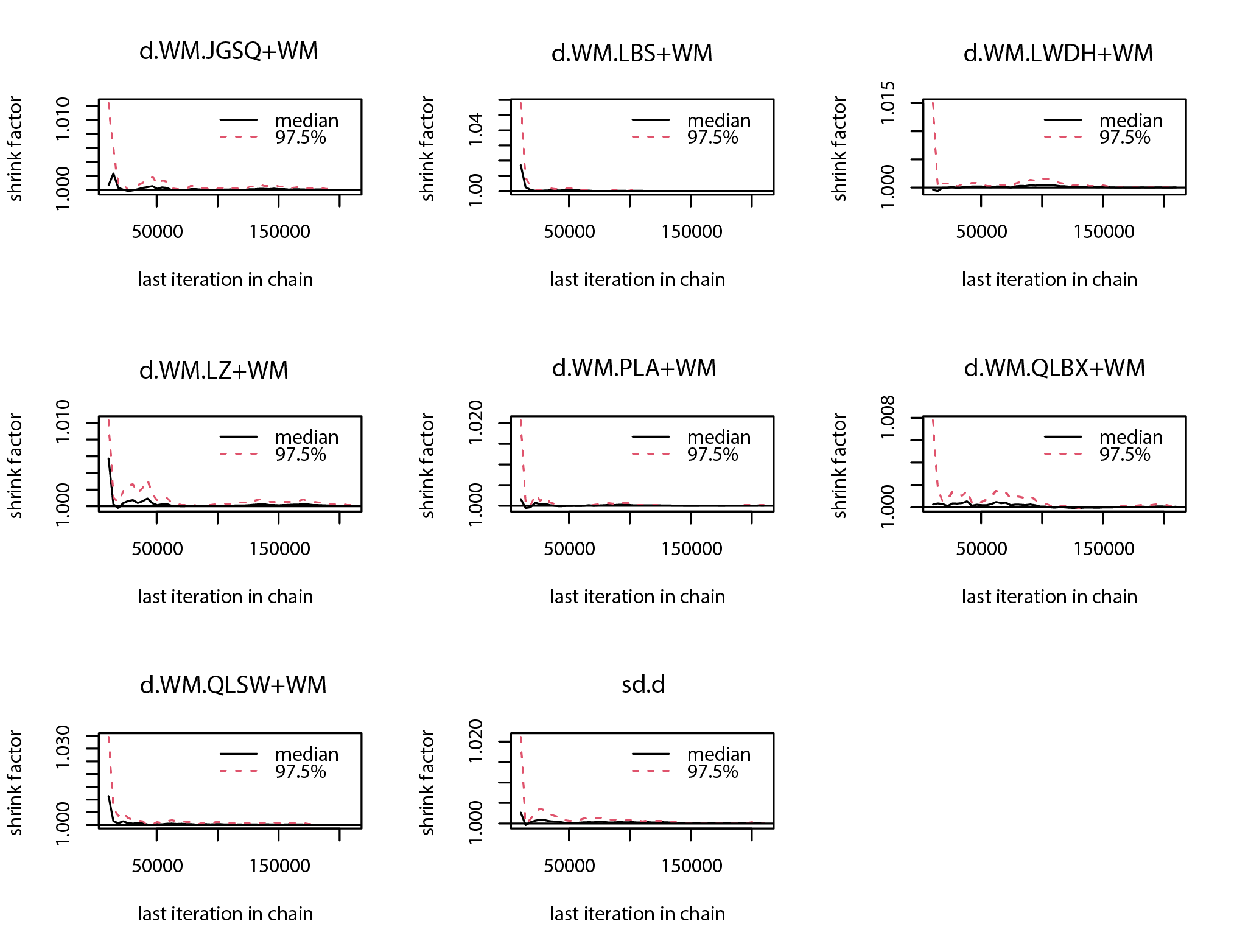
**

## Brooks-Gelman-Rubin plots for the prostate volume.

**
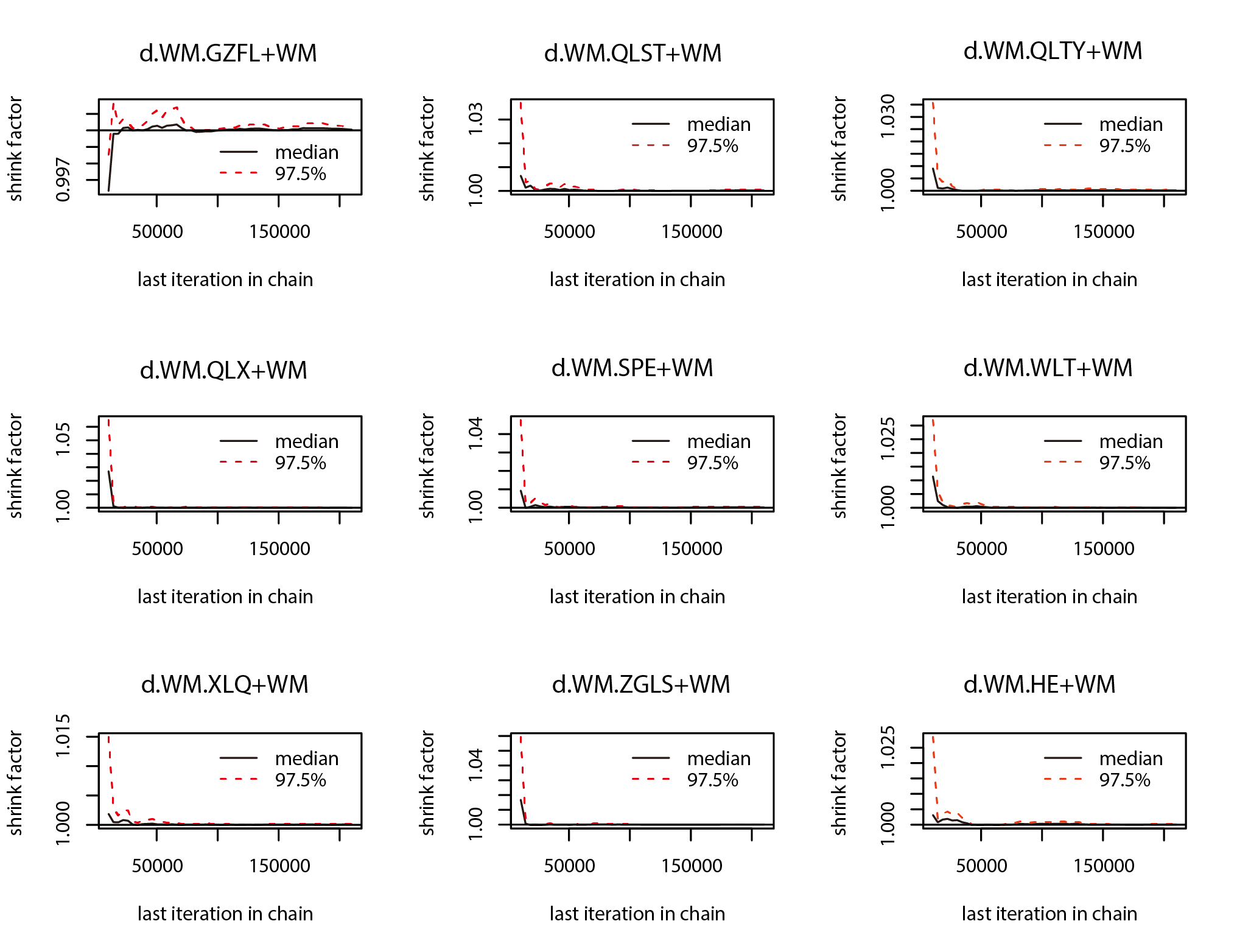
**

**
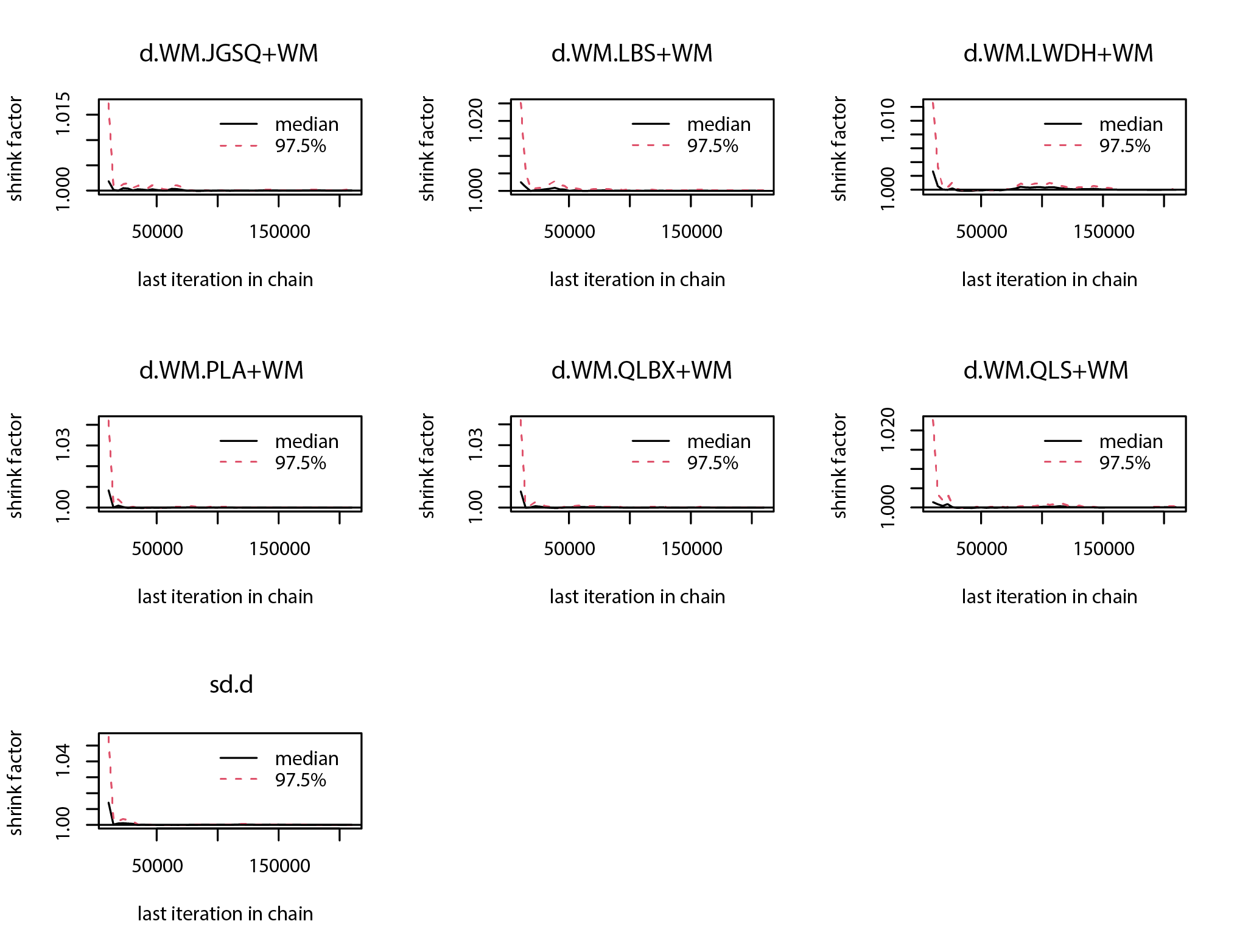
**

## Brooks-Gelman-Rubin plots for postvoid residual urine volume.


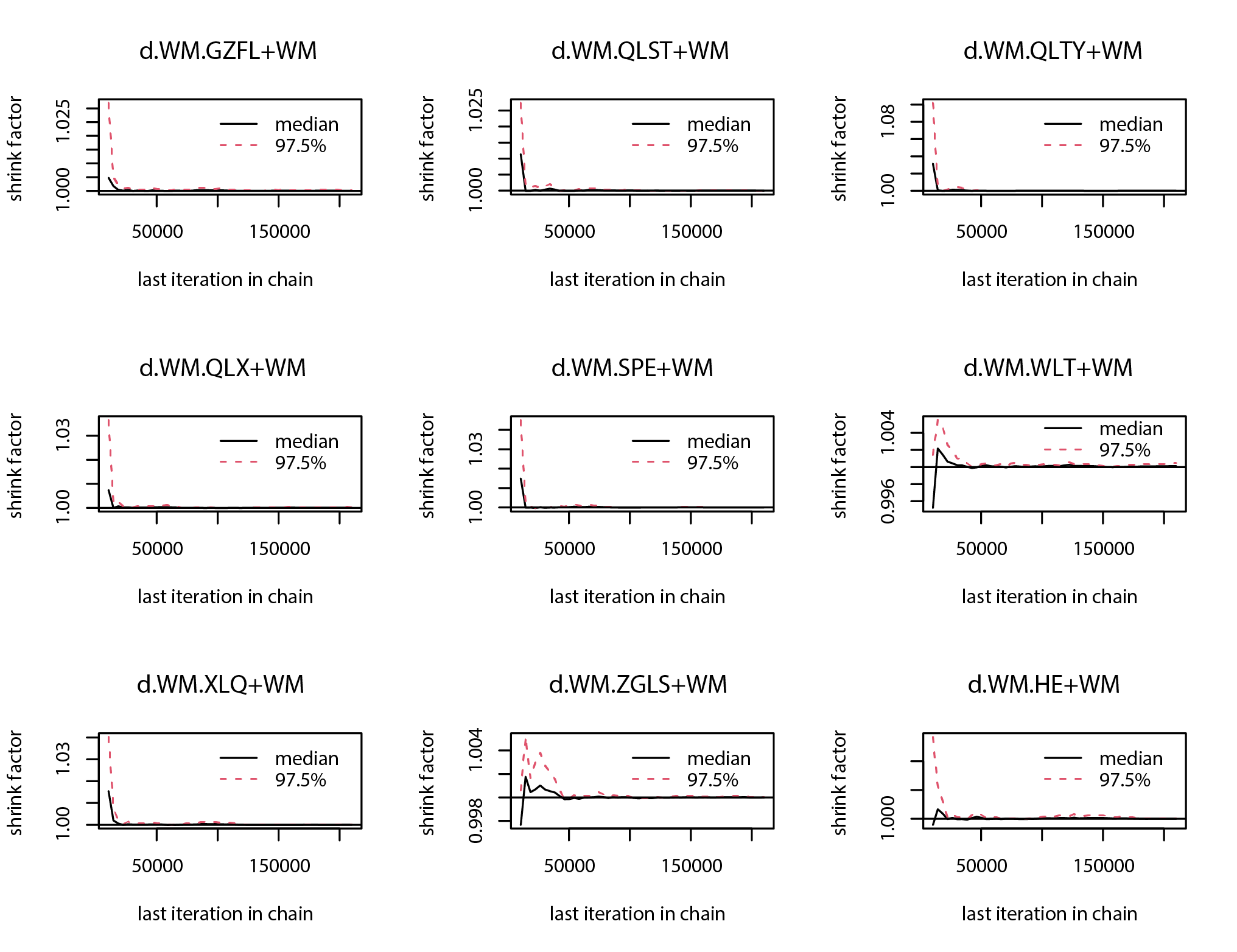


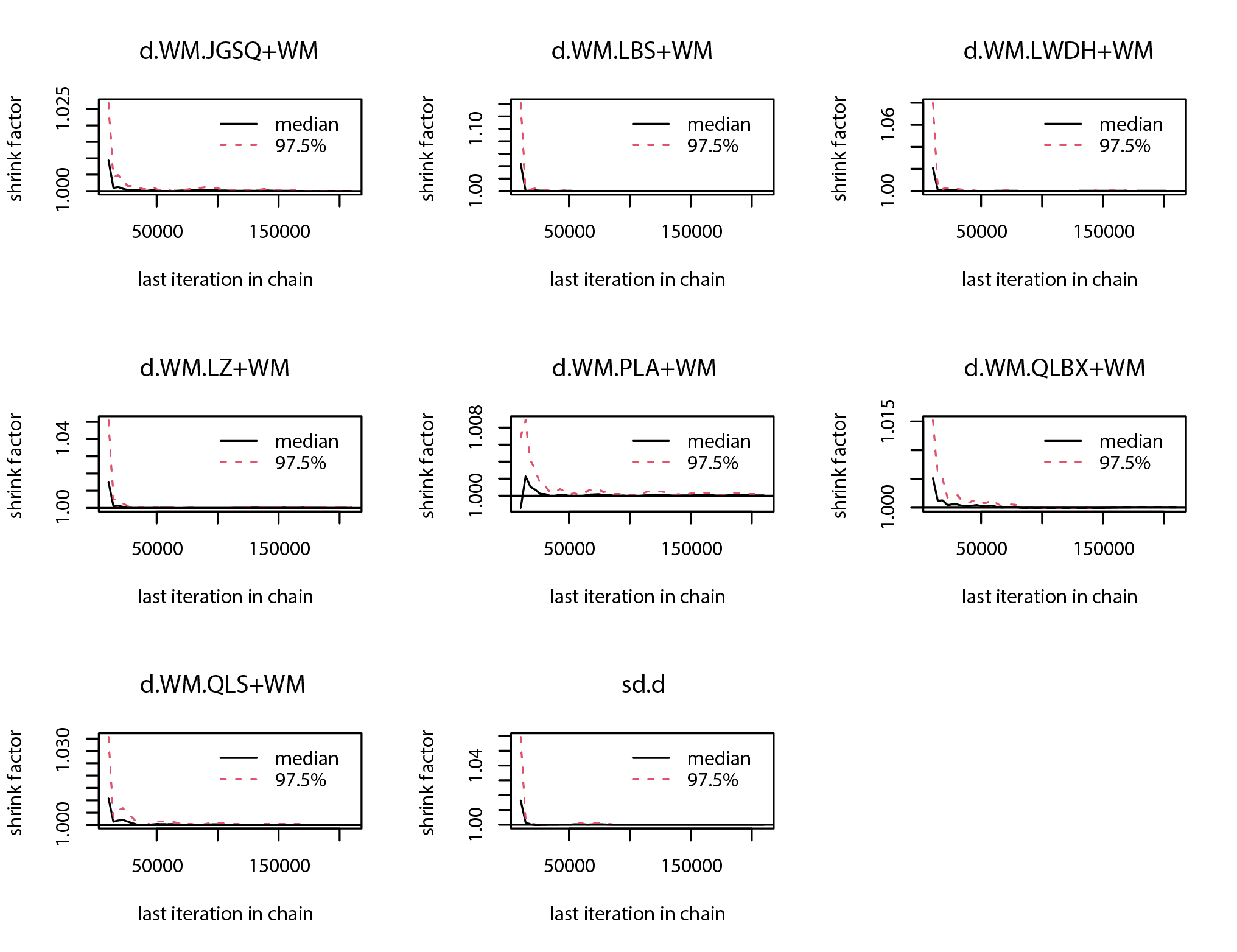


Note: Brooks-Gelman-Rubin plot for the efficacy of the comparison among different treatments in clinical effect rate, IPSS, QoL score, maximum flow rate, prostate volume, postvoid residual urine volume. WM, Western medicine; GZFL, Guizhi Fuling capsule; HE, Huange capsule; JGSQ, Jingui Shenqi pill; LBS, Longbishu capsule; LWDH, Liuwei Dihuang pill; LZ, Lingze tablet; PLA, Pulean tablet; QLBX, Qianlie Beixi capsule; QLS, Qianlieshu pill; QLST, Qianlie Shutong capsule; QLTY, Qianlie Tongyu capsule; QLX, Qianliexin capsule; SPE, Saw Palmetto Extract capsule; WLT, Wenglitong capsule; XLQ, Xialiqi capsule; ZGLS, Zegui Longshuang Capsule.

# File S8: Table of adverse drugs reactions.

| Treatment | Dizziness | Headache | Fatigue | Nausea or vomiting | Gastrointestinal reaction | Skin itching or allergy | Hypotension | Sexual dysfunction | Urethral burning sensation | Total |
| --- | --- | --- | --- | --- | --- | --- | --- | --- | --- | --- |
| GZFL+WM | 3/86(3.49%) | 1/86(1.16%) | 0/86(0%) | 1/86(1.16%) | 1/86(1.16%) | 0/86(0%) | 0/86(0%) | 0/86(0%) | 0/86(0%) | 6/86(6.98%) |
| HE+WM | 0/50(0%) | 1/50(2%) | 0/50(0%) | 0/50(0%) | 1/50(2%) | 1/50(2%) | 0/50(0%) | 0/50(0%) | 0/50(0%) | 3/50(6%) |
| JGSQ+WM | 0/142(0%) | 0/142(0%) | 0/142(0%) | 0/142(0%) | 0/142(0%) | 0/142(0%) | 0/142(0%) | 0/142(0%) | 0/142(0%) | 0/142(0%) |
| LBS+WM | 4/873(0.46%) | 3/873(0.34%) | 0/873(0%) | 21/873(2.41%) | 27/873(3.09%) | 0/873(0%) | 2/873(0.23%) | 2/873(0.23%) | 1/873(0.11%) | 60/873(6.87%) |
| LWDH+WM | 3/62(4.84%) | 0/62(0%) | 0/62(0%) | 0/62(0%) | 0/62(0%) | 0/62(0%) | 0/62(0%) | 0/62(0%) | 0/62(0%) | 3/62(4.84%) |
| LZ+WM | 1/345(0.29%) | 2/345(0.58%) | 0/345(0%) | 2/345(0.58%) | 1/345(0.29%) | 2/345(0.58%) | 0/345(0%) | 0/345(0%) | 0/345(0%) | 8/345(2.32%) |
| PLA+WM | 5/143(3.5%) | 2/143(1.4%) | 0/143(0%) | 0/143(0%) | 3/143(2.1%) | 0/143(0%) | 0/143(0%) | 0/143(0%) | 0/143(0%) | 10/143(6.99%) |
| QLBX+WM | 0/298(0%) | 0/298(0%) | 0/298(0%) | 2/298(0.67%) | 5/298(1.68%) | 0/298(0%) | 0/298(0%) | 2/298(0.67%) | 4/298(1.34%) | 13/298(4.36%) |
| QLST+WM | 23/899(2.56%) | 5/899(0.56%) | 3/899(0.33%) | 15/899(1.67%) | 11/899(1.22%) | 3/899(0.33%) | 7/899(0.78%) | 2/899(0.22%) | 2/899(0.22%) | 71/899(7.9%) |
| QLTY+WM | 0/128(0%) | 0/128(0%) | 0/128(0%) | 0/128(0%) | 6/128(4.69%) | 0/128(0%) | 4/128(3.13%) | 0/128(0%) | 0/128(0%) | 10/128(7.81%) |
| QLX+WM | 5/162(3.09%) | 0/162(0%) | 0/162(0%) | 4/162(2.47%) | 0/162(0%) | 0/162(0%) | 0/162(0%) | 0/162(0%) | 0/162(0%) | 9/162(5.56%) |
| SPE+WM | 6/302(1.99%) | 15/302(4.97%) | 2/302(0.66%) | 1/302(0.33%) | 12/302(3.97%) | 0/302(0%) | 12/302(3.97%) | 26/302(8.61%) | 0/302(0%) | 74/302(24.5%) |
| WLT+WM | 0/41(0%) | 0/41(0%) | 0/41(0%) | 0/41(0%) | 0/41(0%) | 0/41(0%) | 0/41(0%) | 0/41(0%) | 0/41(0%) | 0/41(0%) |
| XLQ+WM | 1/120(0.83%) | 0/120(0%) | 0/120(0%) | 1/120(0.83%) | 0/120(0%) | 0/120(0%) | 0/120(0%) | 1/120(0.83%) | 0/120(0%) | 3/120(2.5%) |
| ZGLS+WM | 5/107(4.67%) | 0/107(0%) | 0/107(0%) | 0/107(0%) | 3/107(2.8%) | 0/107(0%) | 0/107(0%) | 0/107(0%) | 0/107(0%) | 8/107(7.48%) |

# File S9: Final result and goodness of fit of the models for each outcome.

|  | Model type | Dbar | pD | DIC | Data points | Ratio | Global I^2^ |
| --- | --- | --- | --- | --- | --- | --- | --- |
| Clinical effective rate | Random-effect | 116.2873 | 71.54144 | 187.8288 | 138 | 0.8427 | 0% |
|  | Fix-effect | 116.5149 | 70.01823 | 186.5332 | 138 | 0.8443 | 0% |
|  |  |  |  |  |  |  |  |
| IPSS | Random-effect | 191.9088 | 180.0966 | 372.0055 | 191 | 1.005 | 1% |
|  | Fix-effect | 777.5265 | 111.2108 | 888.7373 | 191 | 4.071 | 76% |
|  |  |  |  |  |  |  |  |
| QoL score | Random-effect | 80.5801 | 79.45148 | 160.0316 | 81 | 0.9948 | 0.7% |
|  | Fix-effect | 934.2493 | 55.32114 | 989.5705 | 81 | 11.53 | 91% |
|  |  |  |  |  |  |  |  |
| Maximum urinary flow rate | Random-effect | 178.9056 | 170.3316 | 349.2372 | 179 | 0.9995 | 0.5% |
|  | Fix-effect | 1305.132 | 105.7966 | 1410.929 | 179 | 7.291 | 86% |
|  |  |  |  |  |  |  |  |
| Prostate volume | Random-effect | 154.0513 | 144.095 | 298.1463 | 163 | 0.9451 | 0% |
|  | Fix-effect | 1036.142 | 96.93174 | 1133.073 | 163 | 6.357 | 84% |
|  |  |  |  |  |  |  |  |
| Postvoid residual urine | Random-effect | 173.4197 | 156.1738 | 329.5935 | 175 | 0.991 | 0% |
|  | Fix-effect | 618.5304 | 103.2369 | 721.7673 | 175 | 3.534 | 72% |

# File S10: Heterogeneity detection.

## Predictive interval plot for clinical effective rate.


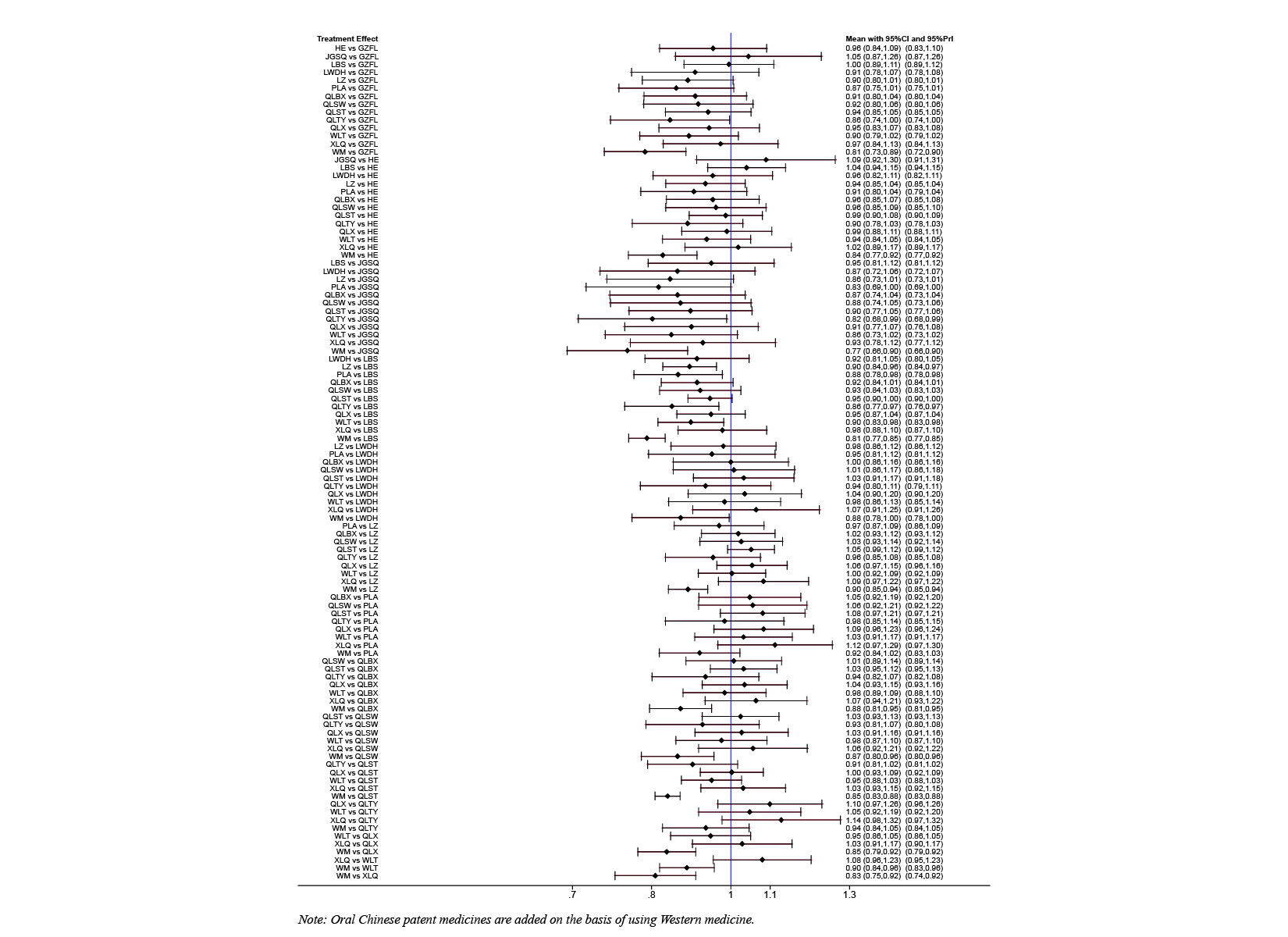


Note: Poly-herbal TCM formulations are added on the basis of using Western medicine. WM, Western medicine; GZFL, Guizhi Fuling capsule; HE, Huange capsule; JGSQ, Jingui Shenqi pill; LBS, Longbishu capsule; LWDH, Liuwei Dihuang pill; LZ, Lingze tablet; PLA, Pulean tablet; QLBX, Qianlie Beixi capsule; QLSW, Qianlieshu pill; QLST, Qianlie Shutong capsule; QLTY, Qianlie Tongyu capsule; QLX, Qianliexin capsule; WLT, Wenglitong capsule; XLQ, Xialiqi capsule.

## Predictive interval plot for IPSS.


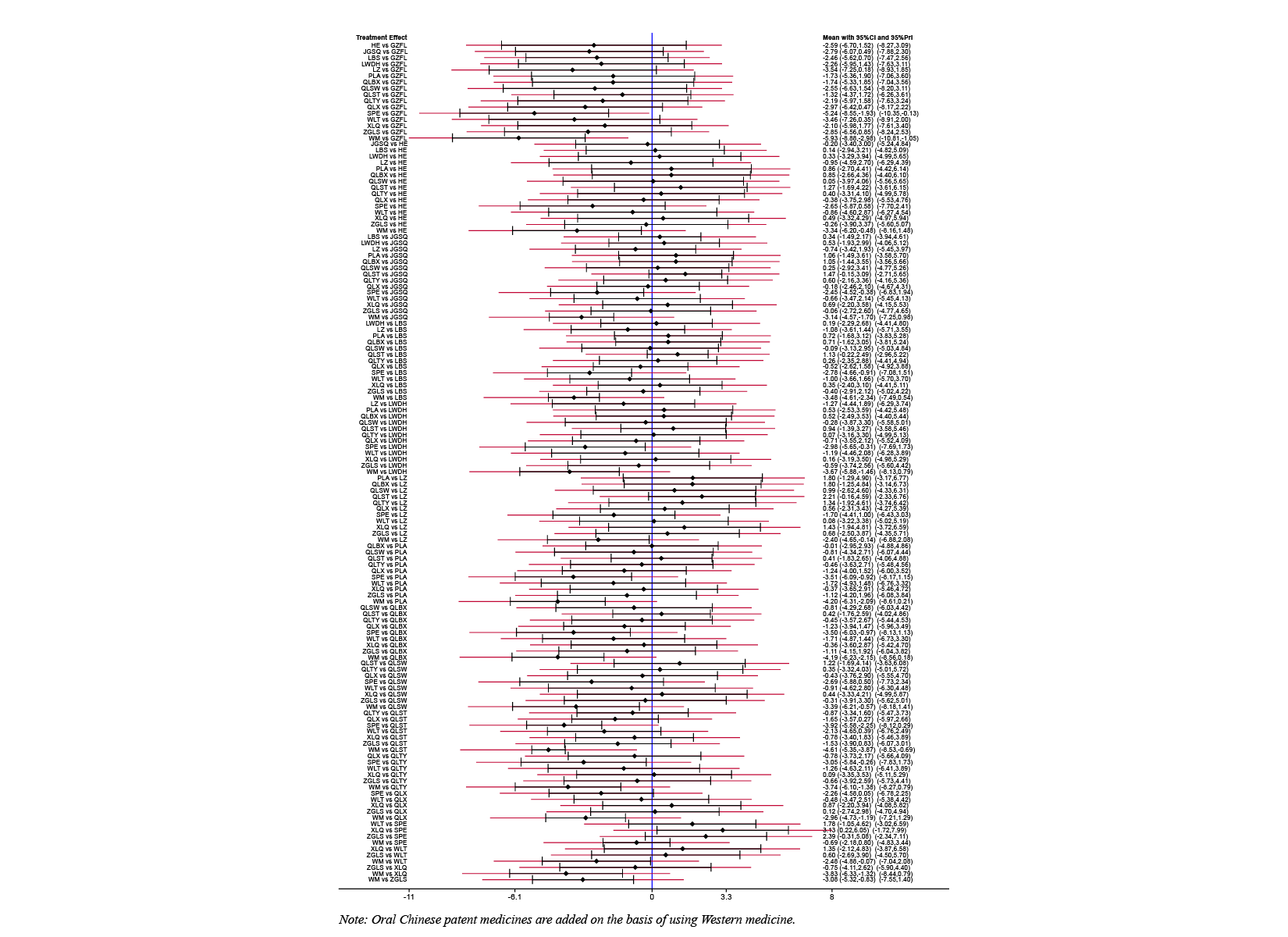


Note: Poly-herbal TCM formulations are added on the basis of using Western medicine. WM, Western medicine; GZFL, Guizhi Fuling capsule; HE, Huange capsule; JGSQ, Jingui Shenqi pill; LBS, Longbishu capsule; LWDH, Liuwei Dihuang pill; LZ, Lingze tablet; PLA, Pulean tablet; QLBX, Qianlie Beixi capsule; QLSW, Qianlieshu pill; QLST, Qianlie Shutong capsule; QLTY, Qianlie Tongyu capsule; QLX, Qianliexin capsule; SPE, Saw Palmetto Extract capsule; WLT, Wenglitong capsule; XLQ, Xialiqi capsule; ZGLS, Zegui Longshuang Capsule.

## Predictive interval plot for QoL score.


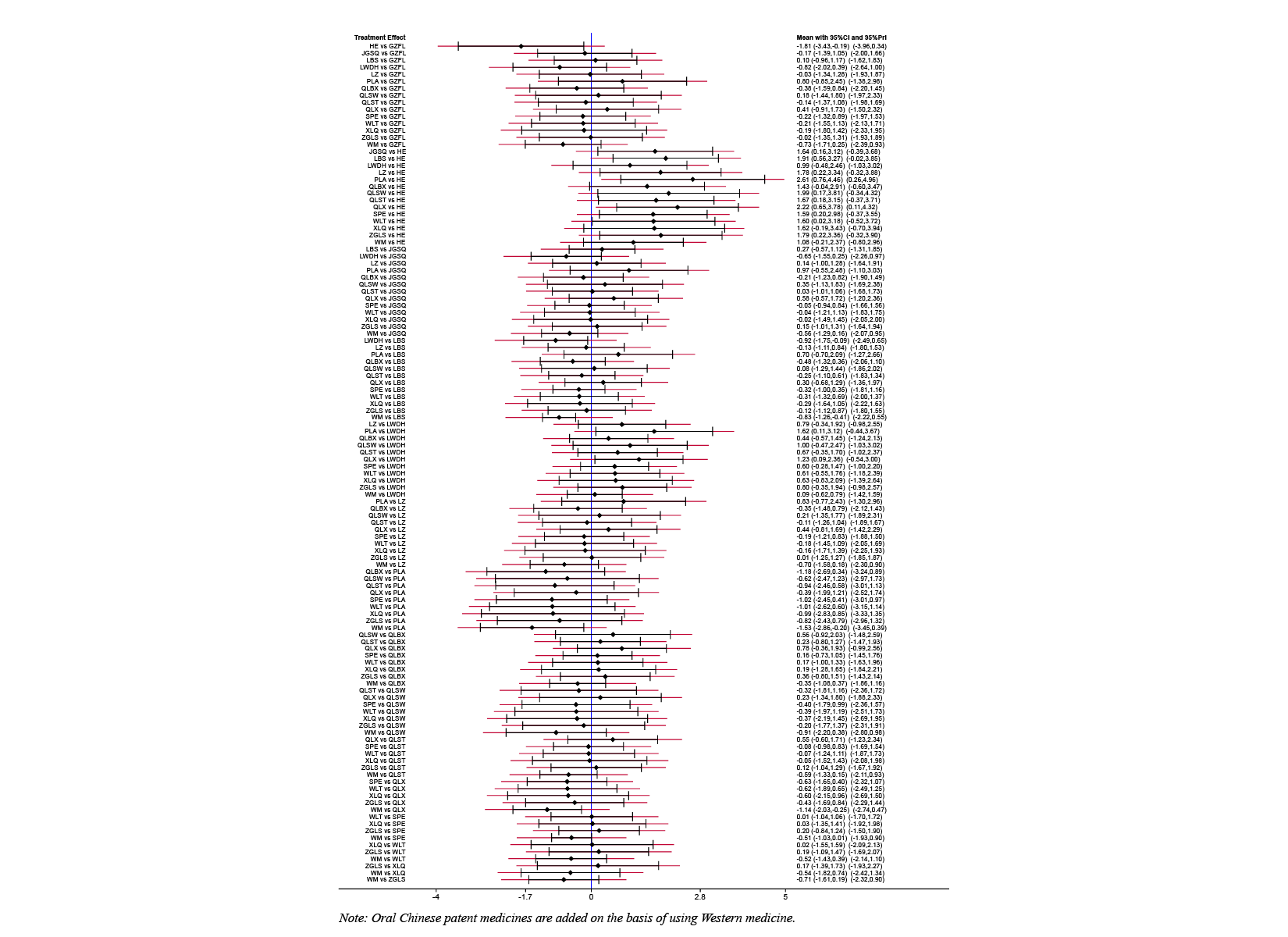


Note: Poly-herbal TCM formulations are added on the basis of using Western medicine. WM, Western medicine; GZFL, Guizhi Fuling capsule; HE, Huange capsule; JGSQ, Jingui Shenqi pill; LBS, Longbishu capsule; LWDH, Liuwei Dihuang pill; LZ, Lingze tablet; PLA, Pulean tablet; QLBX, Qianlie Beixi capsule; QLSW, Qianlieshu pill; QLST, Qianlie Shutong capsule; QLX, Qianliexin capsule; SPE, Saw Palmetto Extract capsule; WLT, Wenglitong capsule; XLQ, Xialiqi capsule; ZGLS, Zegui Longshuang Capsule.

## Predictive interval plot for maximum flow rate of urine.


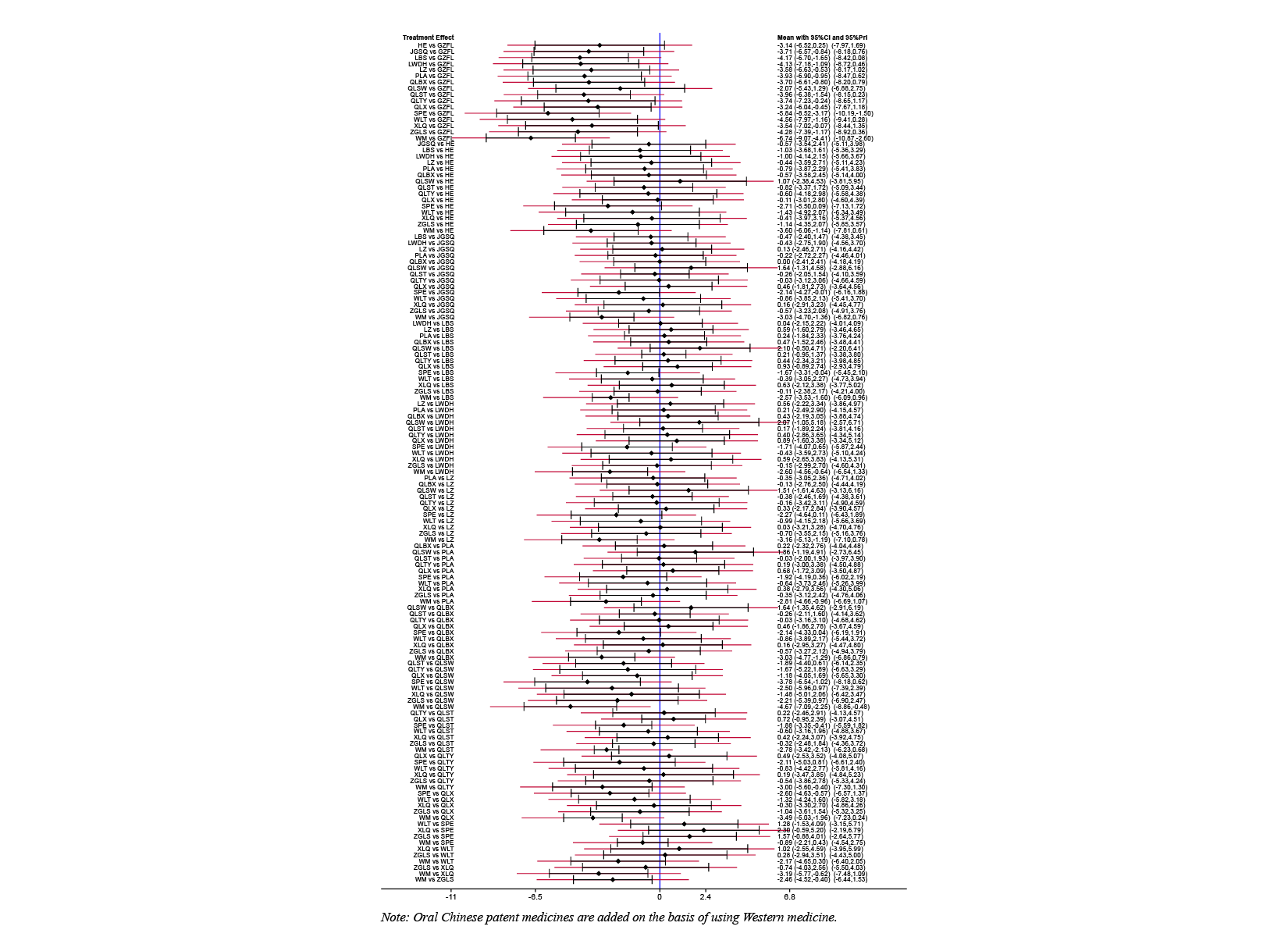


Note: Poly-herbal TCM formulations are added on the basis of using Western medicine. WM, Western medicine; GZFL, Guizhi Fuling capsule; HE, Huange capsule; JGSQ, Jingui Shenqi pill; LBS, Longbishu capsule; LWDH, Liuwei Dihuang pill; LZ, Lingze tablet; PLA, Pulean tablet; QLBX, Qianlie Beixi capsule; QLSW, Qianlieshu pill; QLST, Qianlie Shutong capsule; QLTY, Qianlie Tongyu capsule; QLX, Qianliexin capsule; SPE, Saw Palmetto Extract capsule; WLT, Wenglitong capsule; XLQ, Xialiqi capsule; ZGLS, Zegui Longshuang Capsule.

## Predictive interval plot for prostate volume.


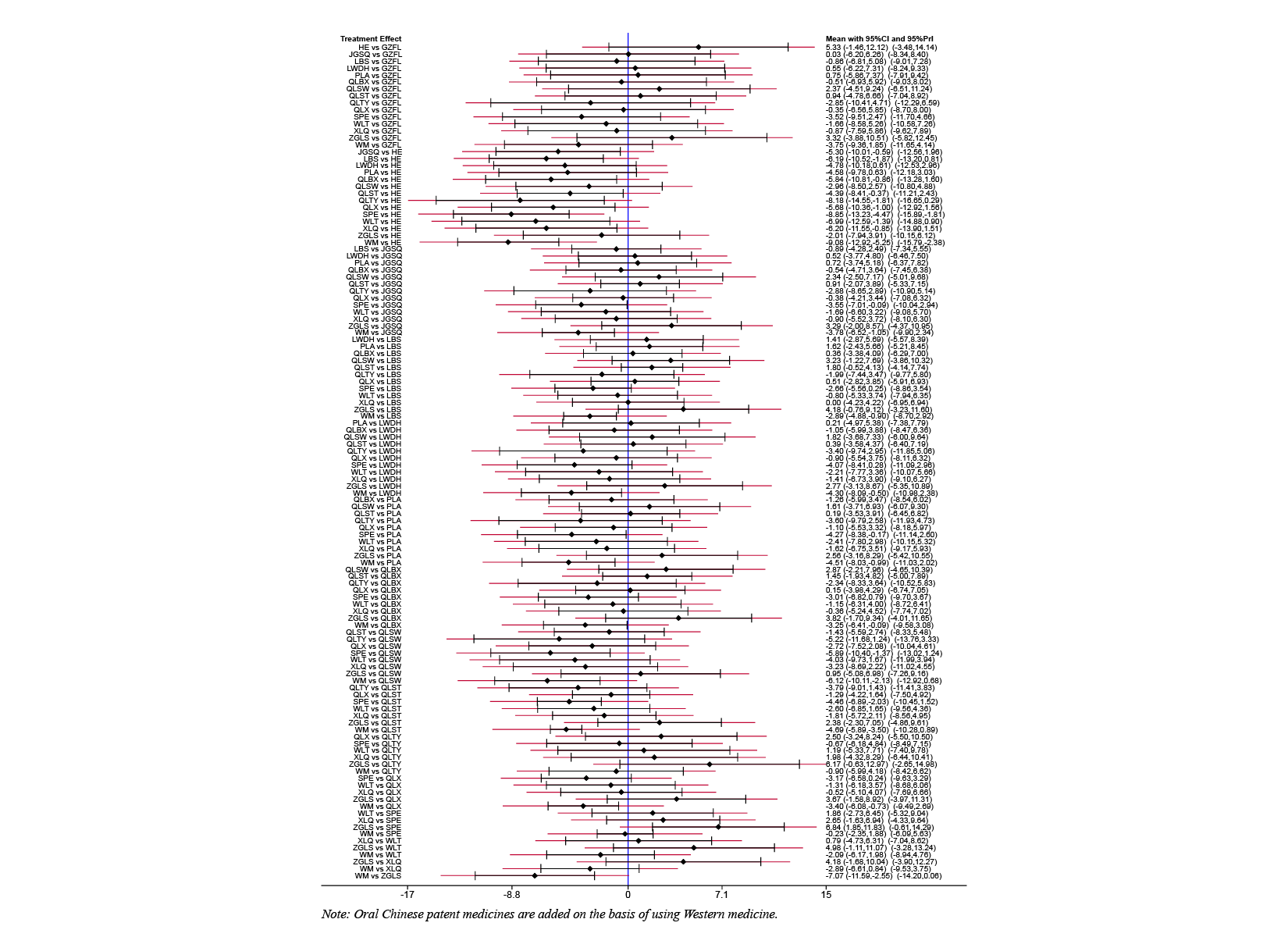


Note: Poly-herbal TCM formulations are added on the basis of using Western medicine. WM, Western medicine; GZFL, Guizhi Fuling capsule; HE, Huange capsule; JGSQ, Jingui Shenqi pill; LBS, Longbishu capsule; LWDH, Liuwei Dihuang pill; PLA, Pulean tablet; QLBX, Qianlie Beixi capsule; QLSW, Qianlieshu pill; QLST, Qianlie Shutong capsule; QLTY, Qianlie Tongyu capsule; QLX, Qianliexin capsule; SPE, Saw Palmetto Extract capsule; WLT, Wenglitong capsule; XLQ, Xialiqi capsule; ZGLS, Zegui Longshuang Capsule.

## Predictive interval plot for postvoid residual urine.


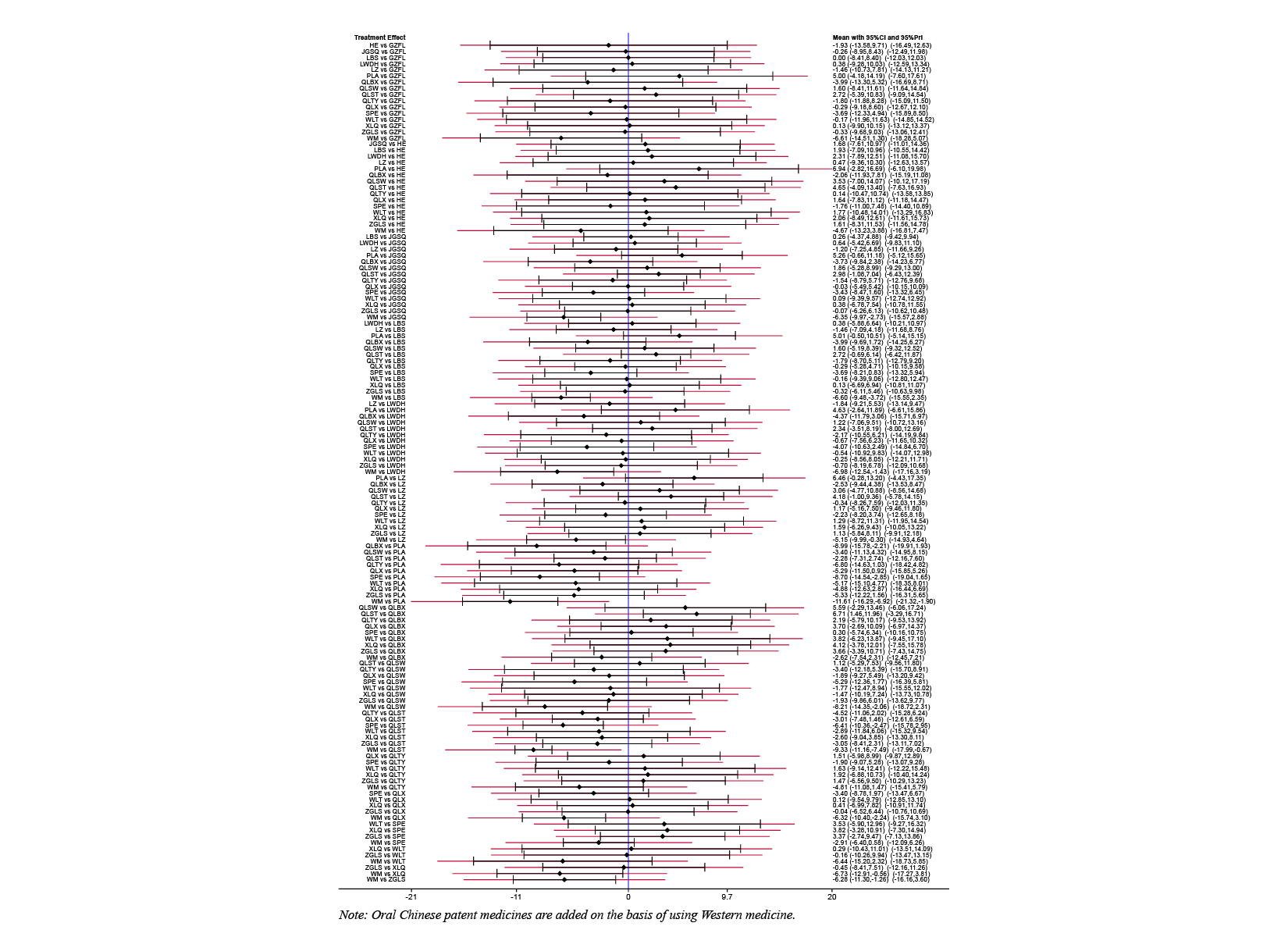


Note: Poly-herbal TCM formulations are added on the basis of using Western medicine. WM, Western medicine; GZFL, Guizhi Fuling capsule; HE, Huange capsule; JGSQ, Jingui Shenqi pill; LBS, Longbishu capsule; LWDH, Liuwei Dihuang pill; LZ, Lingze tablet; PLA, Pulean tablet; QLBX, Qianlie Beixi capsule; QLSW, Qianlieshu pill; QLST, Qianlie Shutong capsule; QLTY, Qianlie Tongyu capsule; QLX, Qianliexin capsule; SPE, Saw Palmetto Extract capsule; WLT, Wenglitong capsule; XLQ, Xialiqi capsule; ZGLS, Zegui Longshuang Capsule.

# File S11: Funnel plots for publication bias.


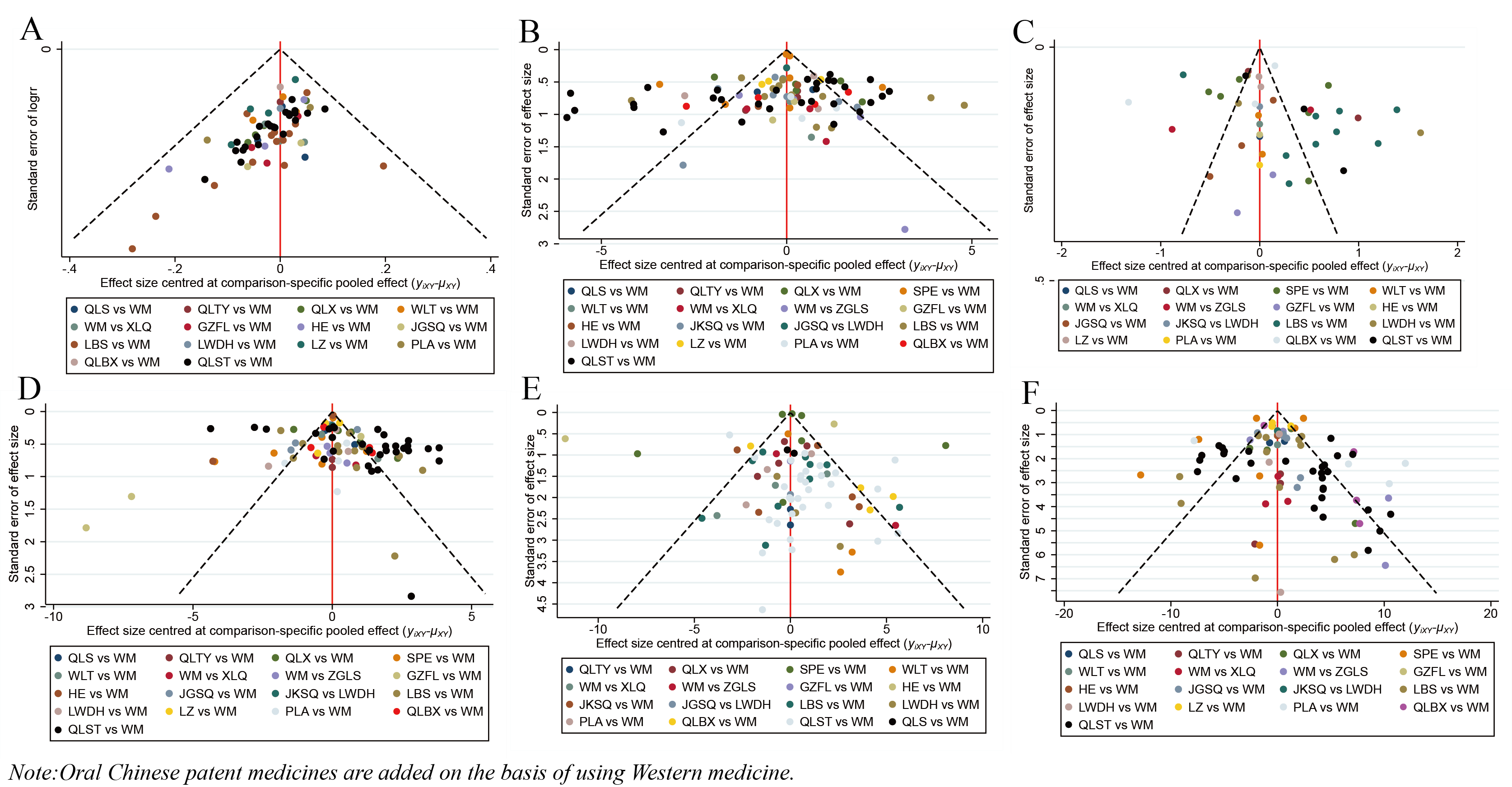


Note: Poly-herbal TCM formulations are added on the basis of using Western medicine. (A) Clinical effective rate; (B) International Prostate Symptom Score; (C) QoL score; (D) Maximum flow rate of urine; (E) Prostate volume; (F) Postvoid residual urine; WM, Western medicine; GZFL, Guizhi Fuling capsule; HE, Huange capsule; JGSQ, Jingui Shenqi pill; LBS, Longbishu capsule; LWDH, Liuwei Dihuang pill; LZ, Lingze tablet; PLA, Pulean tablet; QLBX, Qianlie Beixi capsule; QLS, Qianlieshu pill; QLST, Qianlie Shutong capsule; QLTY, Qianlie Tongyu capsule; QLX, Qianliexin capsule; SPE, Saw Palmetto Extract capsule; WLT, Wenglitong capsule; XLQ, Xialiqi capsule; ZGLS, Zegui Longshuang Capsule.

# File S12: Sensitivity analyses.

## Sensitivity analysis within a network framework focusing on studies published in the past decade.


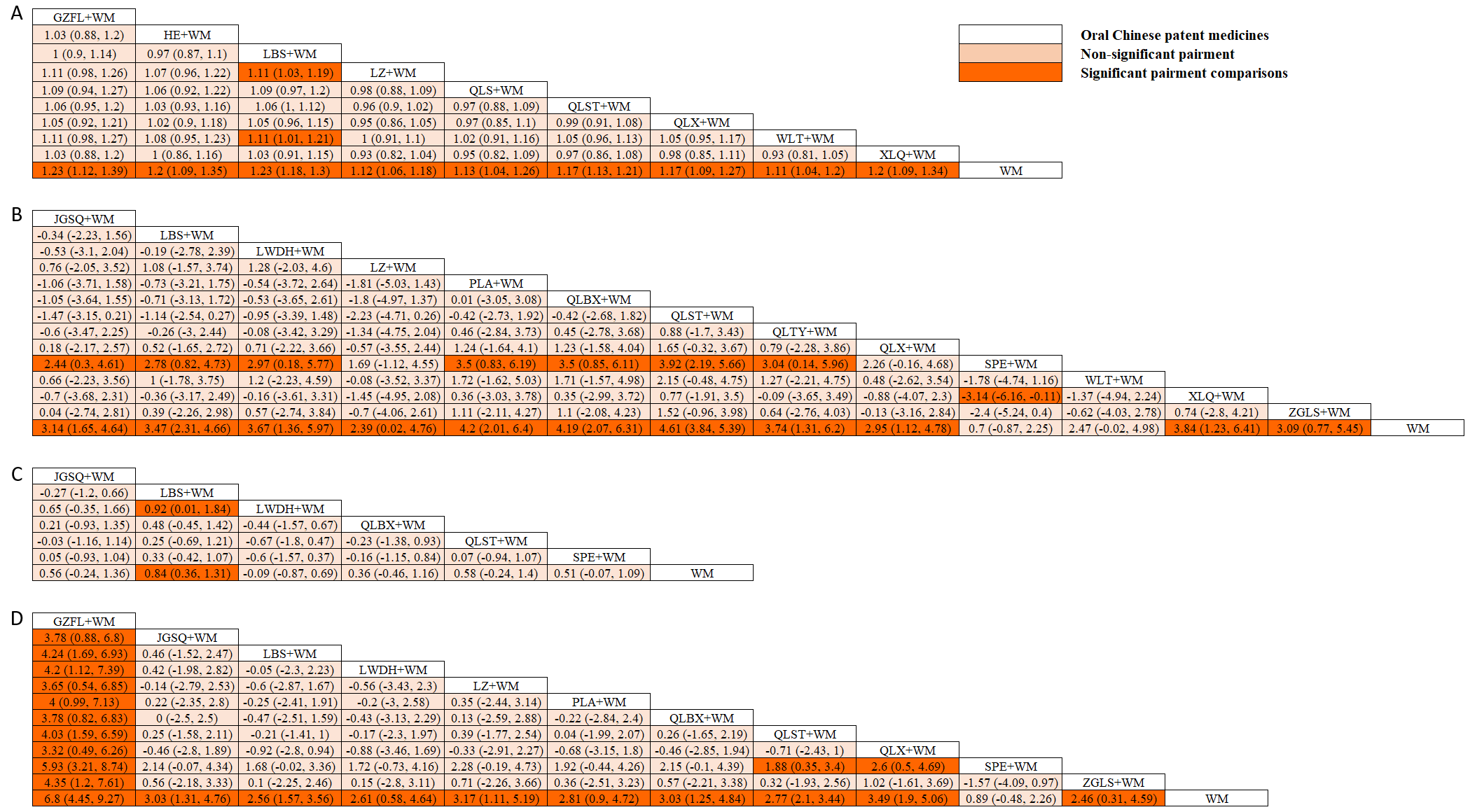


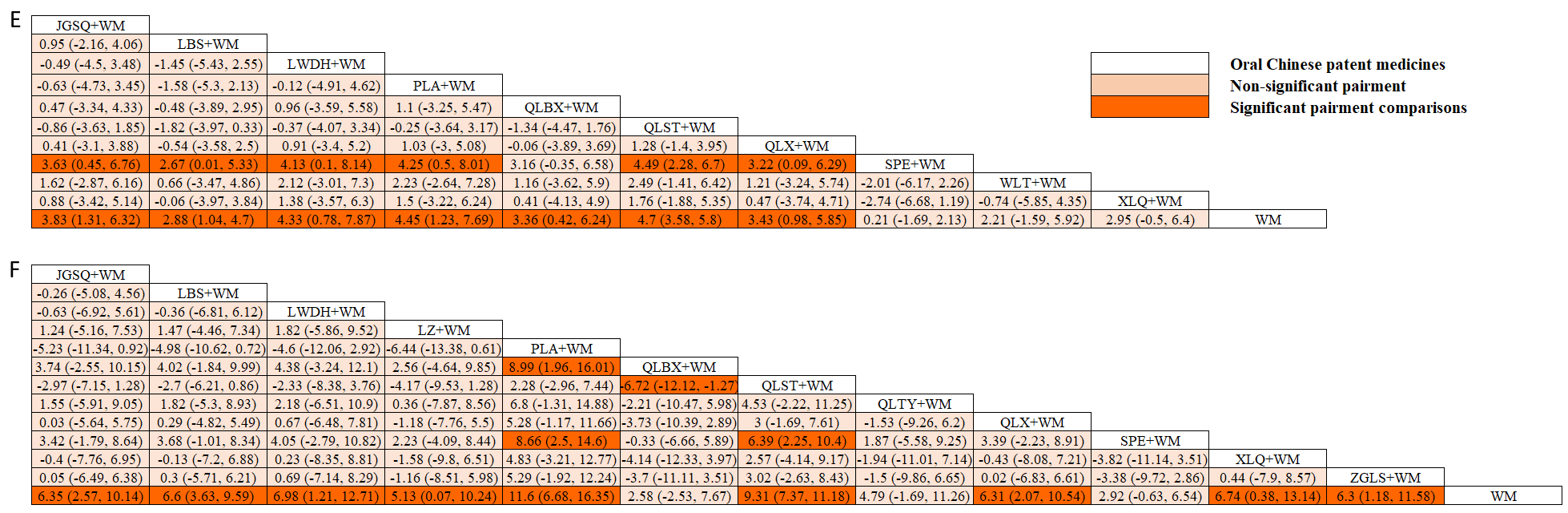


Note: (A) Clinical effective rate; (B) International Prostate Symptom Score; (C) QoL score; (D) Maximum flow rate of urine; (E) Prostate volume; (F) Postvoid residual urine; WM, Western medicine; GZFL, Guizhi Fuling capsule; HE, Huange capsule; JGSQ, Jingui Shenqi pill; LBS, Longbishu capsule; LWDH, Liuwei Dihuang pill; LZ, Lingze tablet; PLA, Pulean tablet; QLBX, Qianlie Beixi capsule; QLS, Qianlieshu pill; QLST, Qianlie Shutong capsule; QLTY, Qianlie Tongyu capsule; QLX, Qianliexin capsule; SPE, Saw Palmetto Extract capsule; WLT, Wenglitong capsule; XLQ, Xialiqi capsule; ZGLS, Zegui Longshuang Capsule.

## Sensitivity analysis within a network framework considering studies that exclude poly-herbal TCM formulations with fewer than three selected RCTs in each outcome.


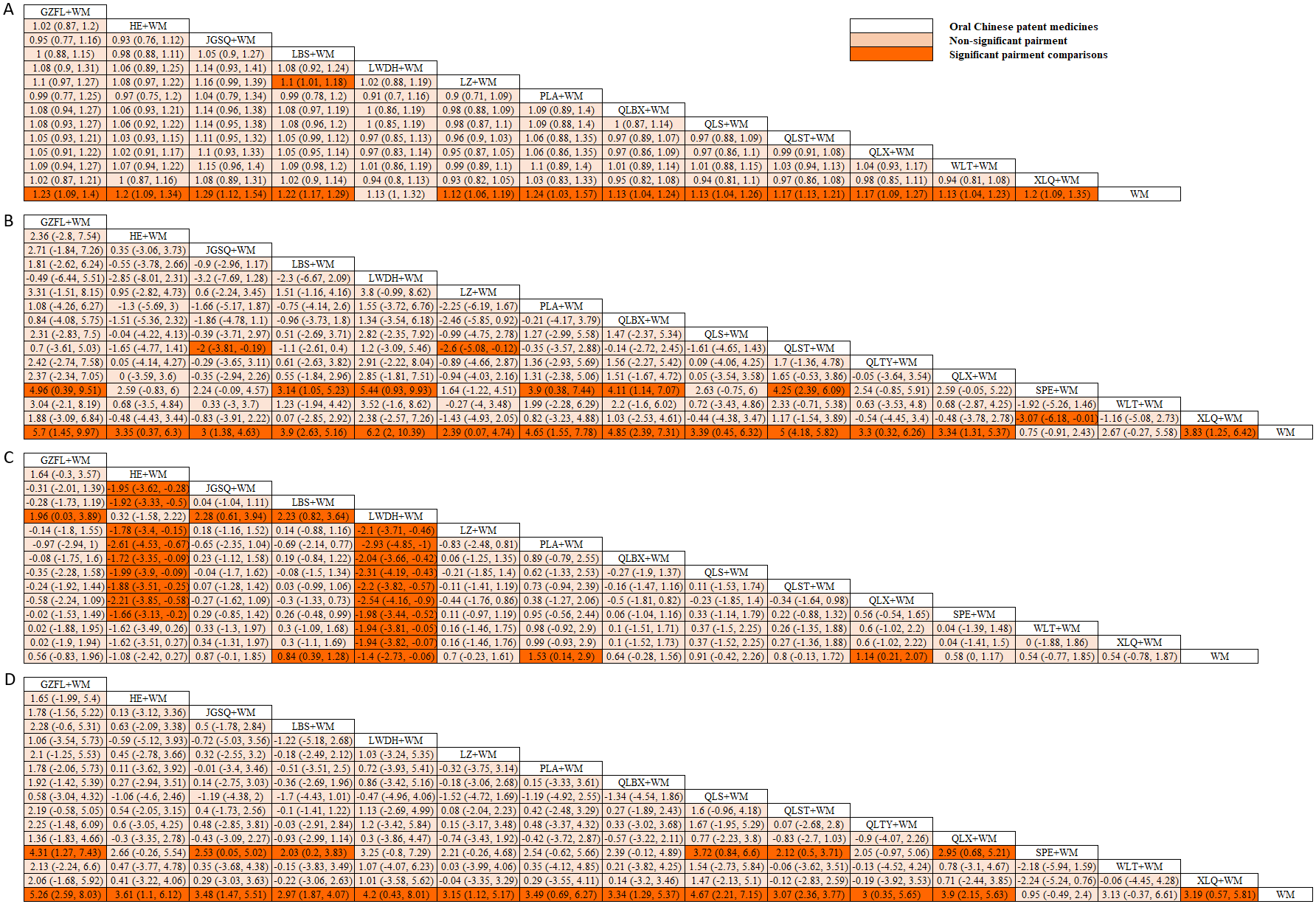


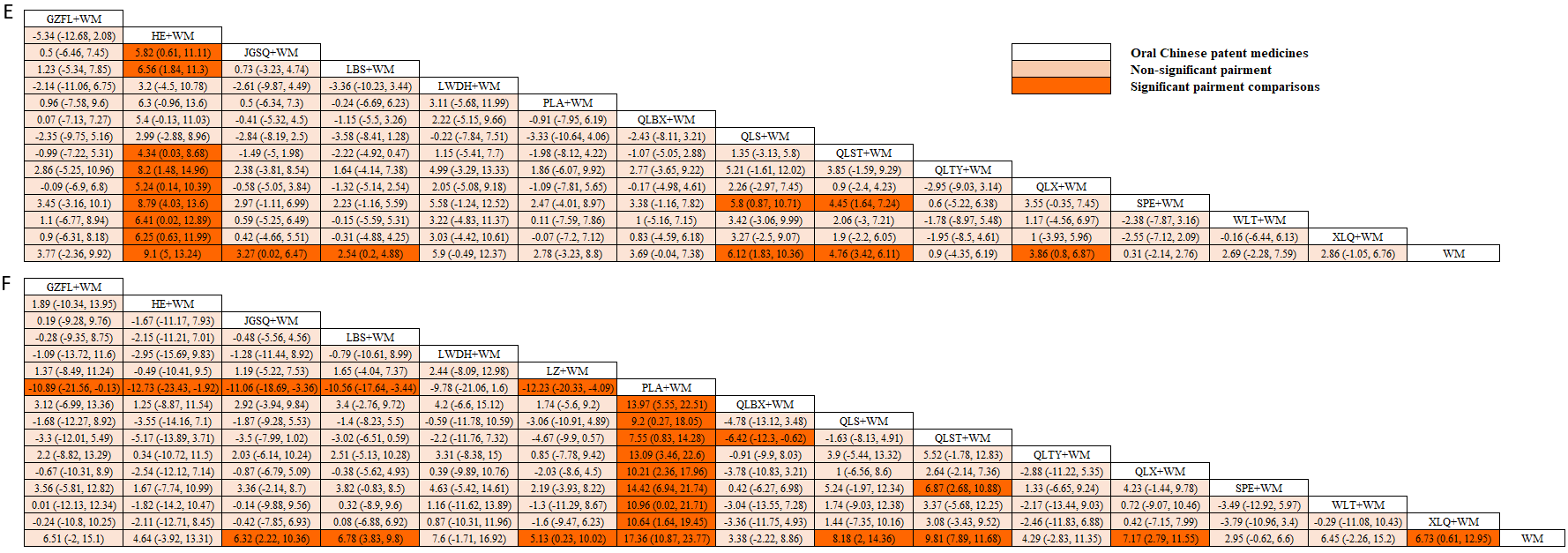


Note: (A) Clinical effective rate; (B) International Prostate Symptom Score; (C) QoL score; (D) Maximum flow rate of urine; (E) Prostate volume; (F) Postvoid residual urine; WM, Western medicine; GZFL, Guizhi Fuling capsule; HE, Huange capsule; JGSQ, Jingui Shenqi pill; LBS, Longbishu capsule; LWDH, Liuwei Dihuang pill; LZ, Lingze tablet; PLA, Pulean tablet; QLBX, Qianlie Beixi capsule; QLS, Qianlieshu pill; QLST, Qianlie Shutong capsule; QLTY, Qianlie Tongyu capsule; QLX, Qianliexin capsule; SPE, Saw Palmetto Extract capsule; WLT, Wenglitong capsule; XLQ, Xialiqi capsule; ZGLS, Zegui Longshuang Capsule.

## Sensitivity analysis within a network framework considering studies of poly-herbal TCM formulations with consistent dosage and usage.


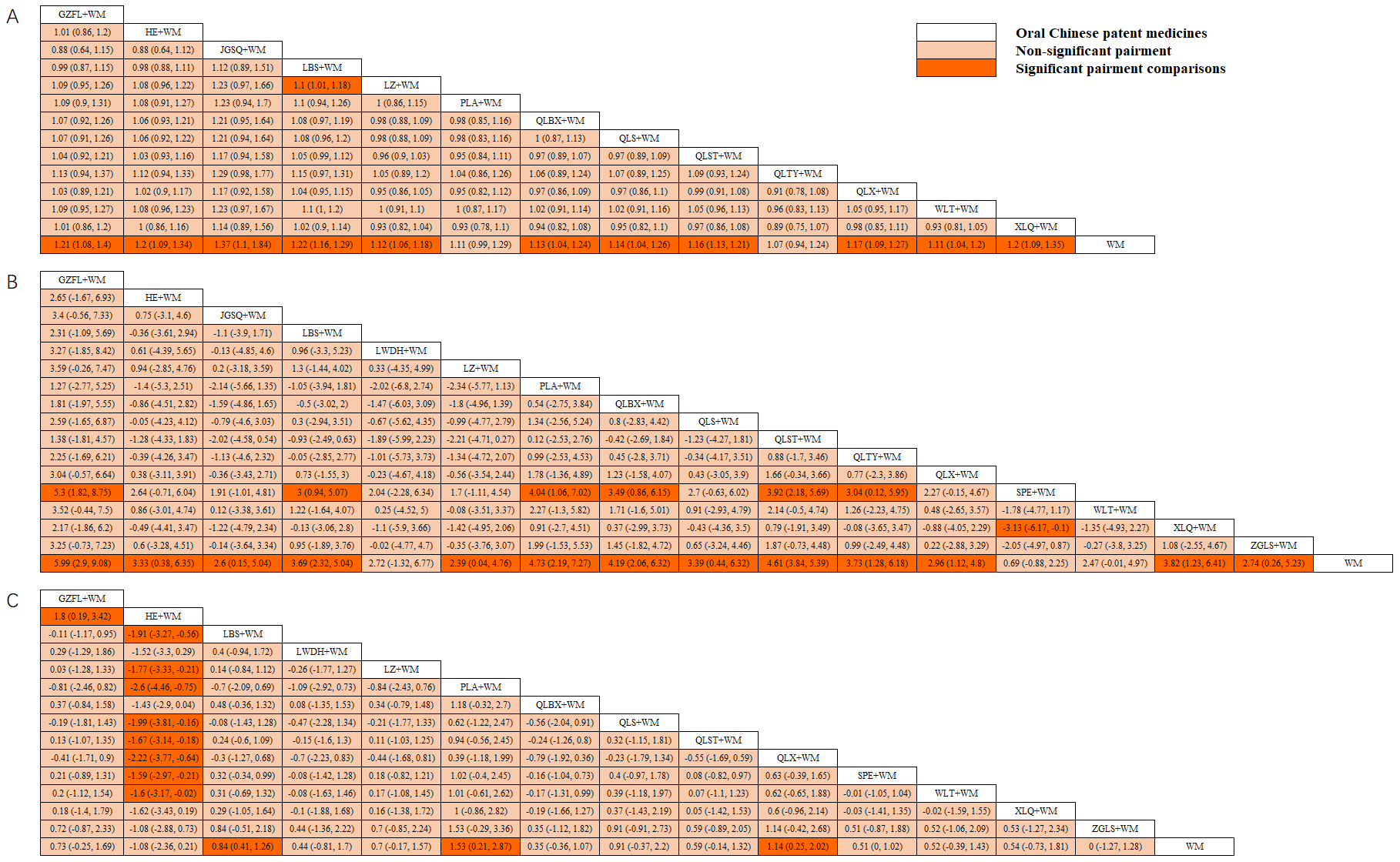


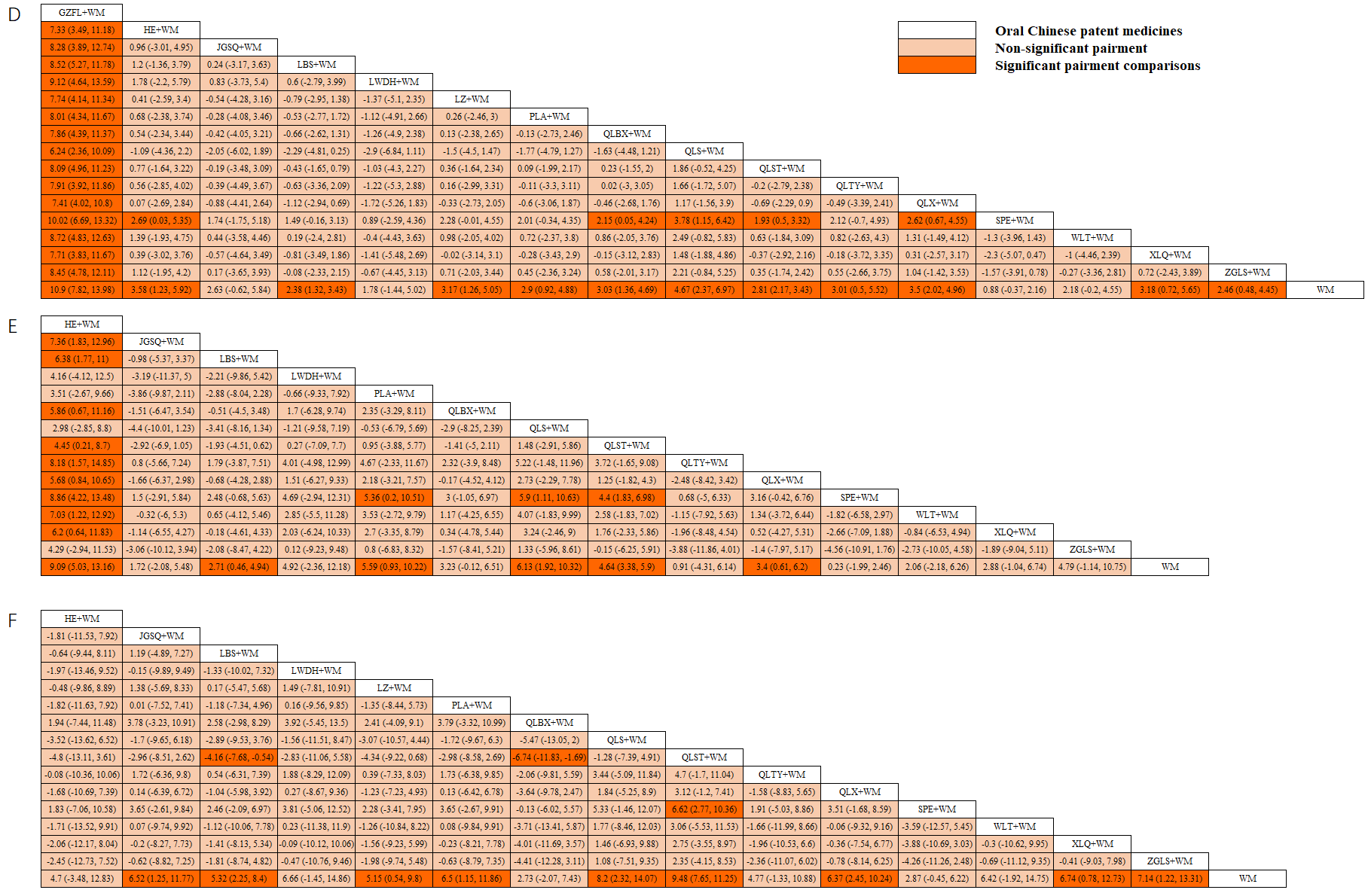


Note: (A) Clinical effective rate; (B) International Prostate Symptom Score; (C) QoL score; (D) Maximum flow rate of urine; (E) Prostate volume; (F) Postvoid residual urine; WM, Western medicine; GZFL, Guizhi Fuling capsule; HE, Huange capsule; JGSQ, Jingui Shenqi pill; LBS, Longbishu capsule; LWDH, Liuwei Dihuang pill; LZ, Lingze tablet; PLA, Pulean tablet; QLBX, Qianlie Beixi capsule; QLS, Qianlieshu pill; QLST, Qianlie Shutong capsule; QLTY, Qianlie Tongyu capsule; QLX, Qianliexin capsule; SPE, Saw Palmetto Extract capsule; WLT, Wenglitong capsule; XLQ, Xialiqi capsule; ZGLS, Zegui Longshuang Capsule.

# File S13: Subgroup network meta-analysis.

## Subgroup network meta-analysis for clinical effective rate

| **Characteristics** | **GZFL+WM** | **HE+WM** | **JGSQ+WM** | **LBS+WM** | **LWDH+WM** | **LZ+WM** | **PLA+WM** | **QLBX+WM** | **QLS+WM** | **QLST+WM** | **QLTY+WM** | **QLX+WM** | **WLT+WM** | **XLQ+WM** |
| --- | --- | --- | --- | --- | --- | --- | --- | --- | --- | --- | --- | --- | --- | --- |
| All trials | **1.23 (1.12, 1.39)** | **1.2 (1.09, 1.34)** | **1.29 (1.11, 1.53)** | **1.23 (1.18, 1.3)** | 1.13 (1, 1.31) | **1.12 (1.06, 1.18)** | 1.11 (0.98, 1.28) | **1.13 (1.04, 1.24)** | **1.14 (1.04, 1.26)** | **1.17 (1.13, 1.21)** | 1.07 (0.95, 1.25) | **1.17 (1.09, 1.27)** | **1.11 (1.04, 1.2)** | **1.2 (1.08, 1.35)** |
| Mean age |  |  |  |  |  |  |  |  |  |  |  |  |  |  |
| ＞65 years | **1.21 (1.08, 1.4)** | **1.2 (1.07, 1.37)** | **1.29 (1.11, 1.54)** | **1.22 (1.15, 1.3)** | 1.13 (0.99, 1.32) | 1.14 (0.98, 1.35) | 1.03 (0.89, 1.23) | NA | **1.12 (1.01, 1.28)** | **1.2 (1.13, 1.28)** | 1.06 (0.93, 1.25) | **1.17 (1.09, 1.28)** | **1.1 (1.01, 1.2)** | **1.2 (1.08, 1.35)** |
| ≤65 years | **1.3 (1.07, 1.67)** | 1.22 (0.99, 1.53) | NA | **1.28 (1.16, 1.41)** | NA | **1.12 (1.05, 1.2)** | **1.24 (1.03, 1.55)** | **1.13 (1.04, 1.25)** | 1.19 (0.98, 1.49) | **1.15 (1.11, 1.2)** | NA | NA | **1.17 (1.01, 1.42)** | NA |
| Trial duration |  |  |  |  |  |  |  |  |  |  |  |  |  |  |
| ≥3 months | **1.21 (1.08, 1.4)** | NA | **1.25 (1.03, 1.56)** | **1.23 (1.17, 1.29)** | 1.13 (0.99, 1.31) | **1.18 (1.04, 1.35)** | 1.03 (0.89, 1.22) | **1.13 (1.04, 1.25)** | **1.12 (1.01, 1.27)** | **1.17 (1.12, 1.22)** | NA | **1.17 (1.07, 1.28)** | **1.12 (1.02, 1.25)** | **1.2 (1.09, 1.34)** |
| ＜3 months | **1.31 (1.06, 1.69)** | **1.21 (1.09, 1.36)** | **1.37 (1.08, 1.88)** | **1.33 (1.12, 1.61)** | NA | **1.11 (1.03, 1.21)** | **1.24 (1.02, 1.57)** | NA | 1.19 (0.97, 1.51) | **1.17 (1.11, 1.25)** | 1.07 (0.92, 1.26) | **1.21 (1.02, 1.49)** | 1.11 (0.98, 1.27) | NA |
| Sample size |  |  |  |  |  |  |  |  |  |  |  |  |  |  |
| ≥100 | **1.2 (1.04, 1.42)** | **1.2 (1.09, 1.35)** | NA | **1.23 (1.16, 1.3)** | 1.13 (0.99, 1.32) | **1.11 (1.03, 1.19)** | NA | **1.13 (1.04, 1.25)** | 1.14 (1, 1.33) | **1.15 (1.1, 1.21)** | NA | 1.12 (0.99, 1.28) | **1.1 (1.01, 1.2)** | 1.17 (1, 1.42) |
| ＜100 | **1.28 (1.1, 1.53)** | NA | **1.29 (1.12, 1.53)** | **1.26 (1.15, 1.39)** | NA | **1.16 (1.04, 1.33)** | 1.11 (0.98, 1.28) | NA | 1.14 (0.99, 1.35) | **1.19 (1.13, 1.25)** | 1.07 (0.94, 1.26) | **1.22 (1.1, 1.37)** | **1.17 (1.01, 1.42)** | **1.23 (1.07, 1.44)** |

Note: Bold and underlined fonts indicate statistical differences; WM, Western medicine; GZFL, Guizhi Fuling capsule; HE, Huange capsule; JGSQ, Jingui Shenqi pill; LBS, Longbishu capsule; LWDH, Liuwei Dihuang pill; LZ, Lingze tablet; PLA, Pulean tablet; QLBX, Qianlie Beixi capsule; QLS, Qianlieshu pill; QLST, Qianlie Shutong capsule; QLTY, Qianlie Tongyu capsule; QLX, Qianliexin capsule; WLT, Wenglitong capsule; XLQ, Xialiqi capsule.

## Subgroup network meta-analysis for IPSS

| **Characteristics** | **GZFL+WM** | **HE+WM** | **JGSQ+WM** | **LBS+WM** | **LWDH+WM** | **LZ+WM** | **PLA+WM** | **QLBX+WM** | **QLS+WM** | **QLST+WM** | **QLTY+WM** | **QLX+WM** | **SPE+WM** | **WLT+WM** | **XLQ+WM** | **ZGLS+WM** |
| --- | --- | --- | --- | --- | --- | --- | --- | --- | --- | --- | --- | --- | --- | --- | --- | --- |
| All trials | **5.99 (2.95, 9.02)** | **3.34 (0.44, 6.27)** | **3.14 (1.67, 4.61)** | **3.47 (2.31, 4.63)** | **3.67 (1.41, 5.95)** | **2.39 (0.09, 4.71)** | **4.21 (2.04, 6.39)** | **4.19 (2.11, 6.28)** | **3.39 (0.51, 6.26)** | **4.61 (3.85, 5.37)** | **3.73 (1.32, 6.12)** | **2.96 (1.14, 4.77)** | 0.69 (-0.84, 2.23) | **2.48 (0.02, 4.94)** | **3.83 (1.28, 6.39)** | **3.08 (0.79, 5.39)** |
| Mean age |  |  |  |  |  |  |  |  |  |  |  |  |  |  |  |  |
| ＞65 years | **5.98 (3.12, 8.82)** | 2.83 (-0.93, 6.55) | **3.14 (1.44, 4.81)** | **3.54 (2.13, 4.93)** | **4.05 (1.57, 6.54)** | 1.41 (-2.29, 5.09) | **3.9 (0.02, 7.78)** | 2.31 (-1.49, 6.12) | **4.14 (0.32, 7.95)** | **3.74 (2.62, 4.87)** | **3.73 (1.49, 6)** | **2.96 (1.28, 4.65)** | 0.35 (-2.23, 2.93) | 2.31 (-0.64, 5.26) | **3.82 (1.43, 6.21)** | 1.23 (-2.61, 5.1) |
| ≤65 years | NA | 3.9 (-1.21, 8.92) | **3.36 (0.39, 6.35)** | **3.38 (1.16, 5.57)** | 2.6 (-2.49, 7.7) | 2.89 (-0.55, 6.32) | **4.33 (1.35, 7.3)** | **4.86 (1.97, 7.76)** | 2.64 (-2.26, 7.56) | **5.2 (4.04, 6.37)** | NA | NA | 0.84 (-1.37, 3.04) | 2.75 (-2.09, 7.58) | NA | **3.97 (0.78, 7.2)** |
| Trial duration |  |  |  |  |  |  |  |  |  |  |  |  |  |  |  |  |
| ≥3 months | **5.99 (2.83, 9.17)** | NA | **2.93 (1.29, 4.59)** | **3.41 (2.15, 4.66)** | **4.02 (1.21, 6.82)** | 2.8 (-1.39, 6.98) | NA | **4.2 (2.01, 6.37)** | 4.12 (-0.16, 8.39) | **5.19 (4.14, 6.25)** | NA | **3.41 (0.99, 5.84)** | 1.17 (-0.54, 2.89) | 2.43 (-0.75, 5.57) | **3.84 (1.18, 6.51)** | 1.22 (-3.11, 5.52) |
| ＜3 months | NA | **3.33 (0.29, 6.41)** | **4.88 (0.59, 9.11)** | 4.16 (-0.1, 8.4) | 2.61 (-1.91, 7.07) | 2.19 (-0.79, 5.17) | **4.21 (1.94, 6.47)** | NA | 2.64 (-1.65, 6.96) | **3.86 (2.65, 5.08)** | **3.74 (1.23, 6.29)** | 2.24 (-0.82, 5.29) | -2.19 (-6.44, 2.06) | 2.55 (-1.88, 6.96) | NA | **3.93 (1.07, 6.82)** |
| Sample size |  |  |  |  |  |  |  |  |  |  |  |  |  |  |  |  |
| ≥100 | **5.7 (0.96, 10.45)** | **3.34 (0.03, 6.67)** | 2.32 (-1.03, 5.64) | **3.51 (1.85, 5.15)** | **6.19 (1.52, 10.85)** | 2.9 (-0.34, 6.13) | 3.88 (-0.82, 8.57) | **3.49 (0.17, 6.82)** | 4.14 (-0.56, 8.76) | **5.26 (3.95, 6.57)** | 3.31 (-1.33, 7.93) | 1.87 (-2.7, 6.43) | 0.34 (-2.26, 2.94) | 2.28 (-1.22, 5.75) | 2.6 (-2.23, 7.4) | 2.71 (-2.04, 7.44) |
| ＜100 | **6.3 (1.59, 10.97)** | NA | **3.28 (1.47, 5.12)** | **3.42 (1.34, 5.49)** | 2.66 (-0.25, 5.61) | 1.4 (-2.89, 5.75) | **4.32 (1.65, 7.02)** | **4.92 (1.75, 8.15)** | 2.63 (-1.75, 7.02) | **4.12 (3.05, 5.21)** | **3.97 (0.79, 7.17)** | **3.24 (1.05, 5.41)** | 1 (-1.23, 3.22) | 2.78 (-1.56, 7.04) | **4.51 (1.12, 7.9)** | **3.27 (0.39, 6.19)** |

Note: Bold and underlined fonts indicate statistical differences; WM, Western medicine; GZFL, Guizhi Fuling capsule; HE, Huange capsule; JGSQ, Jingui Shenqi pill; LBS, Longbishu capsule; LWDH, Liuwei Dihuang pill; LZ, Lingze tablet; PLA, Pulean tablet; QLBX, Qianlie Beixi capsule; QLS, Qianlieshu pill; QLST, Qianlie Shutong capsule; QLTY, Qianlie Tongyu capsule; QLX, Qianliexin capsule; SPE, Saw Palmetto Extract capsule; WLT, Wenglitong capsule; XLQ, Xialiqi capsule; ZGLS, Zegui Longshuang Capsule.

## Subgroup network meta-analysis for QoL score

| **Characteristics** | **GZFL+WM** | **HE+WM** | **JGSQ+WM** | **LBS+WM** | **LWDH+WM** | **LZ+WM** | **PLA+WM** | **QLBX+WM** | **QLS+WM** | **QLST+WM** | **QLX+WM** | **SPE+WM** | **WLT+WM** | **XLQ+WM** | **ZGLS+WM** |
| --- | --- | --- | --- | --- | --- | --- | --- | --- | --- | --- | --- | --- | --- | --- | --- |
| All trials | 0.73 (-0.3, 1.76) | -1.08 (-2.45, 0.3) | 0.56 (-0.21, 1.35) | **0.84 (0.37, 1.3)** | -0.09 (-0.85, 0.67) | 0.7 (-0.24, 1.65) | **1.53 (0.12, 2.95)** | 0.35 (-0.42, 1.13) | 0.91 (-0.47, 2.27) | 0.59 (-0.21, 1.38) | **1.14 (0.18, 2.08)** | 0.51 (-0.05, 1.06) | 0.52 (-0.46, 1.5) | 0.54 (-0.83, 1.89) | 0.71 (-0.26, 1.67) |
| Mean age |  |  |  |  |  |  |  |  |  |  |  |  |  |  |  |
| ＞65 years | 0.73 (-0.49, 1.96) | -1.07 (-2.73, 0.6) | 0.3 (-0.83, 1.43) | **0.9 (0.28, 1.54)** | -0.36 (-1.49, 0.76) | NA | NA | -0.2 (-1.82, 1.42) | 0.91 (-0.74, 2.56) | 1.09 (-0.54, 2.71) | 1.13 (-0.01, 2.28) | 0.45 (-0.7, 1.6) | 0.52 (-0.65, 1.7) | 0.54 (-1.11, 2.19) | NA |
| ≤65 years | NA | NA | 1.04 (-0.53, 2.62) | 0.6 (-0.49, 1.69) | 0.35 (-1.14, 1.86) | 0.7 (-0.36, 1.77) | 1.53 (-0.06, 3.11) | 0.64 (-0.43, 1.71) | NA | 0.31 (-0.8, 1.39) | NA | 0.54 (-0.24, 1.29) | NA | NA | 0.71 (-0.38, 1.77) |
| Trial duration |  |  |  |  |  |  |  |  |  |  |  |  |  |  |  |
| ≥3 months | 0.73 (-0.36, 1.83) | NA | 0.52 (-0.31, 1.36) | **0.86 (0.34, 1.39)** | -0.3 (-1.29, 0.68) | 0.69 (-0.76, 2.12) | NA | 0.35 (-0.48, 1.18) | 0.92 (-0.55, 2.38) | 0.8 (-0.21, 1.82) | 0.57 (-0.88, 2.02) | 0.51 (-0.09, 1.1) | 0.5 (-1, 1.98) | 0.54 (-0.91, 2) | NA |
| ＜3 months | NA | -1.07 (-3.39, 1.24) | NA | 0.62 (-1.71, 2.94) | 0.36 (-1.94, 2.65) | 0.7 (-1.61, 3.01) | 1.53 (-0.83, 3.86) | NA | NA | 0.1 (-2.26, 2.47) | 1.67 (-0.66, 3.98) | NA | 0.55 (-1.77, 2.87) | NA | 0.71 (-0.94, 2.34) |
| Sample size |  |  |  |  |  |  |  |  |  |  |  |  |  |  |  |
| ≥100 | 0.56 (-0.65, 1.78) | -1.08 (-2.24, 0.06) | NA | **0.71 (0.28, 1.15)** | **-1.4 (-2.55, -0.25)** | 0.7 (-0.06, 1.47) | NA | 0.52 (-0.25, 1.3) | 0.91 (-0.23, 2.07) | 0.5 (-0.61, 1.62) | 0.57 (-0.56, 1.69) | 0.27 (-0.37, 0.91) | 0.52 (-0.29, 1.33) | 0.54 (-0.59, 1.68) | 0 (-1.15, 1.15) |
| ＜100 | 0.92 (-0.93, 2.79) | NA | 0.68 (-0.32, 1.7) | 1.23 (-0.02, 2.45) | 0.48 (-0.69, 1.65) | NA | 1.53 (-0.27, 3.32) | 0 (-1.75, 1.76) | NA | 0.63 (-0.64, 1.86) | 1.68 (-0.05, 3.41) | 0.77 (-0.28, 1.76) | NA | NA | 1.4 (-0.35, 3.16) |

Note: Bold and underlined fonts indicate statistical differences; WM, Western medicine; GZFL, Guizhi Fuling capsule; HE, Huange capsule; JGSQ, Jingui Shenqi pill; LBS, Longbishu capsule; LWDH, Liuwei Dihuang pill; LZ, Lingze tablet; PLA, Pulean tablet; QLBX, Qianlie Beixi capsule; QLS, Qianlieshu pill; QLST, Qianlie Shutong capsule; QLX, Qianliexin capsule; SPE, Saw Palmetto Extract capsule; WLT, Wenglitong capsule; XLQ, Xialiqi capsule; ZGLS, Zegui Longshuang Capsule.

## Subgroup network meta-analysis for maximum flow rate of urine

| **Characteristics** | **GZFL+WM** | **HE+WM** | **JGSQ+WM** | **LBS+WM** | **LWDH+WM** | **LZ+WM** | **PLA+WM** | **QLBX+WM** | **QLS+WM** | **QLST+WM** | **QLTY+WM** | **QLX+WM** | **SPE+WM** | **WLT+WM** | **XLQ+WM** | **ZGLS+WM** |
| --- | --- | --- | --- | --- | --- | --- | --- | --- | --- | --- | --- | --- | --- | --- | --- | --- |
| All trials | **6.8 (4.46, 9.23)** | **3.59 (1.1, 6.15)** | **3.03 (1.32, 4.74)** | **2.56 (1.57, 3.54)** | **2.61 (0.58, 4.62)** | **3.16 (1.14, 5.17)** | **2.8 (0.93, 4.7)** | **3.02 (1.25, 4.8)** | **4.68 (2.17, 7.16)** | **2.77 (2.11, 3.44)** | **3.01 (0.35, 5.65)** | **3.5 (1.92, 5.07)** | 0.89 (-0.46, 2.25) | 2.17 (-0.38, 4.7) | **3.2 (0.55, 5.82)** | **2.45 (0.34, 4.57)** |
| Mean age |  |  |  |  |  |  |  |  |  |  |  |  |  |  |  |  |
| ＞65 years | **10.89 (8.06, 13.72)** | 1.64 (-1.05, 4.32) | **2.86 (1.3, 4.44)** | **2.13 (1.1, 3.13)** | **3.04 (1.1, 5)** | NA | 2.09 (-0.73, 4.92) | 2.1 (-0.79, 4.99) | **5.19 (2.45, 7.92)** | **2.4 (1.52, 3.26)** | **2.99 (0.81, 5.18)** | **3.52 (2.26, 4.79)** | 0.29 (-1.65, 2.22) | 1.09 (-1.92, 4.13) | **3.18 (1.01, 5.35)** | 2.92 (-0.19, 5.98) |
| ≤65 years | 1.98 (-1.83, 5.86) | **5.96 (1.91, 10.03)** | 3.87 (-0.02, 7.76) | **3.31 (1.55, 5.07)** | 1.34 (-2.59, 5.32) | **3.15 (0.93, 5.36)** | **3.1 (0.7, 5.51)** | **3.34 (1.07, 5.57)** | **4.12 (0.26, 8)** | **3.02 (2.09, 3.93)** | NA | NA | 1.16 (-0.62, 2.94) | 3.13 (-0.71, 6.93) | NA | 2.25 (-0.52, 5.02) |
| Trial duration |  |  |  |  |  |  |  |  |  |  |  |  |  |  |  |  |
| ≥3 months | **10.9 (7.94, 13.85)** | NA | **3.27 (1.54, 5)** | **2.46 (1.52, 3.37)** | **3.18 (1.07, 5.3)** | **3.25 (0.3, 6.18)** | NA | **3.04 (1.49, 4.58)** | **5.18 (2.21, 8.16)** | **3.47 (2.67, 4.25)** | NA | **4.17 (2.46, 5.9)** | 1.18 (-0.08, 2.45) | 2.2 (-0.02, 4.4) | **3.19 (0.86, 5.52)** | 2.92 (-0.38, 6.21) |
| ＜3 months | 1.98 (-1.78, 5.77) | **3.6 (0.92, 6.34)** | 2.62 (-1.09, 6.34) | **3.45 (0.6, 6.28)** | 1.34 (-2.58, 5.22) | **3.11 (0.43, 5.83)** | **2.81 (0.8, 4.83)** | NA | **4.12 (0.3, 7.98)** | **2.02 (0.97, 3.05)** | **3.01 (0.15, 5.84)** | 2.37 (-0.38, 5.11) | -1.1 (-4.96, 2.79) | NA | NA | 2.25 (-0.5, 5.02) |
| Sample size |  |  |  |  |  |  |  |  |  |  |  |  |  |  |  |  |
| ≥100 | **10.2 (5.58, 14.82)** | **3.62 (0.83, 6.48)** | NA | **3.31 (1.92, 4.66)** | **4.2 (0.02, 8.35)** | **3 (0.26, 5.73)** | 2.09 (-1.84, 6.05) | **2.96 (0.17, 5.72)** | **5.19 (1.3, 9.11)** | **3.37 (2.19, 4.52)** | 3 (-1.11, 7.08) | 3.79 (-0.1, 7.7) | 0.24 (-2.01, 2.48) | 1.09 (-2.98, 5.17) | 2.49 (-1.56, 6.56) | 2.2 (-1.8, 6.18) |
| ＜100 | **5.16 (2.47, 8.13)** | NA | **2.92 (1.25, 4.62)** | 1.16 (-0.46, 2.78) | 1.99 (-0.32, 4.28) | 3.53 (-0.04, 7.08) | **3.1 (0.91, 5.29)** | **3.11 (0.63, 5.62)** | **4.11 (0.67, 7.59)** | **2.38 (1.54, 3.21)** | 3 (-0.74, 6.74) | **3.42 (1.68, 5.14)** | 1.23 (-0.59, 3.02) | 3.12 (-0.31, 6.51) | **3.92 (0.23, 7.61)** | **2.6 (0.05, 5.16)** |

Note: Bold and underlined fonts indicate statistical differences; WM, Western medicine; GZFL, Guizhi Fuling capsule; HE, Huange capsule; JGSQ, Jingui Shenqi pill; LBS, Longbishu capsule; LWDH, Liuwei Dihuang pill; LZ, Lingze tablet; PLA, Pulean tablet; QLBX, Qianlie Beixi capsule; QLS, Qianlieshu pill; QLST, Qianlie Shutong capsule; QLTY, Qianlie Tongyu capsule; QLX, Qianliexin capsule; SPE, Saw Palmetto Extract capsule; WLT, Wenglitong capsule; XLQ, Xialiqi capsule; ZGLS, Zegui Longshuang Capsule.

## Subgroup network meta-analysis for prostate volume

| **Characteristics** | **GZFL+WM** | **HE+WM** | **JGSQ+WM** | **LBS+WM** | **LWDH+WM** | **PLA+WM** | **QLBX+WM** | **QLS+WM** | **QLST+WM** | **QLTY+WM** | **QLX+WM** | **SPE+WM** | **WLT+WM** | **XLQ+WM** | **ZGLS+WM** |
| --- | --- | --- | --- | --- | --- | --- | --- | --- | --- | --- | --- | --- | --- | --- | --- |
| All trials | 3.76 (-2.19, 9.73) | **9.09 (5.13, 13.03)** | **3.77 (0.97, 6.57)** | **2.89 (0.84, 4.91)** | **4.3 (0.41, 8.16)** | **4.51 (0.9, 8.12)** | **3.25 (0.01, 6.45)** | **6.12 (2, 10.24)** | **4.69 (3.46, 5.92)** | 0.91 (-4.26, 6.06) | **3.39 (0.65, 6.12)** | 0.23 (-1.93, 2.41) | 2.09 (-2.11, 6.21) | 2.88 (-0.92, 6.67) | **7.07 (2.47, 11.73)** |
| Mean age |  |  |  |  |  |  |  |  |  |  |  |  |  |  |  |
| ＞65 years | NA | **2.23 (0.15, 4.3)** | **2.86 (1.14, 4.59)** | **2.95 (1.71, 4.13)** | **4.59 (1.93, 7.21)** | **5.09 (1.85, 8.3)** | 1.43 (-3.46, 6.29) | **6.31 (3.72, 8.86)** | **4.46 (3.23, 5.69)** | 0.9 (-2.79, 4.53) | **3.48 (2.18, 4.79)** | 0.06 (-1.37, 1.47) | 0.35 (-4.73, 5.38) | **3.33 (1.02, 5.58)** | NA |
| ≤65 years | 3.74 (-3.14, 10.6) | **16.2 (9.61, 22.77)** | **7.02 (0.31, 13.73)** | 3.15 (-1.37, 7.63) | 2.56 (-6.3, 11.5) | 4.23 (-0.81, 9.31) | 3.62 (-0.54, 7.72) | 5.92 (-0.81, 12.71) | **4.78 (3.05, 6.48)** | NA | NA | 0.32 (-2.72, 3.36) | 3.45 (-3.05, 9.95) | NA | **7.21 (2.09, 12.52)** |
| Trial duration |  |  |  |  |  |  |  |  |  |  |  |  |  |  |  |
| ≥3 months | NA | NA | **4.36 (1.7, 6.97)** | **2.99 (1.25, 4.73)** | **4.95 (1.3, 8.53)** | NA | **3.58 (0.88, 6.15)** | **6.3 (1.89, 10.65)** | **4.87 (3.55, 6.24)** | NA | **3.46 (0.9, 6.01)** | 1.5 (-0.26, 3.3) | 2.69 (-1, 6.29) | 3.04 (-0.15, 6.11) | NA |
| ＜3 months | 3.75 (-4.1, 11.69) | **9.14 (3.75, 14.63)** | 2.87 (-4.83, 10.58) | 2.19 (-5.71, 10.13) | 2.65 (-7.19, 12.38) | 4.57 (-0.12, 9.28) | NA | 5.92 (-1.93, 13.78) | **3.92 (1.52, 6.33)** | 0.9 (-5.5, 7.26) | 3.12 (-3.02, 9.12) | -7.69 (-15.43, 0.06) | 0.68 (-9.77, 11.23) | NA | **7.32 (1.4, 13.34)** |
| Sample size |  |  |  |  |  |  |  |  |  |  |  |  |  |  |  |
| ≥100 | NA | **9.09 (4.58, 13.67)** | 1.07 (-6.42, 8.53) | 2.12 (-0.63, 4.87) | 5.89 (-1.07, 12.93) | 5.09 (-1.86, 11.89) | **5.09 (0.01, 10.03)** | 6.29 (-0.3, 12.92) | **4.93 (2.88, 6.93)** | 0.9 (-6.88, 8.63) | 4.07 (-2.66, 10.82) | 0.31 (-3.35, 4) | 0.37 (-6.21, 7.02) | 5.36 (-1.59, 12.38) | 4.83 (-1.82, 11.41) |
| ＜100 | 3.77 (-2.5, 10.08) | NA | **4.2 (0.94, 7.43)** | **4.57 (0.82, 8.43)** | 3.47 (-1.63, 8.65) | 4.21 (-0.48, 8.95) | 1.08 (-3.86, 5.98) | 5.95 (-0.19, 12.1) | **4.48 (2.75, 6.22)** | 0.91 (-6.88, 8.66) | 3.21 (-0.07, 6.45) | 0.17 (-2.98, 3.36) | 3.44 (-2.51, 9.39) | 1.34 (-3.65, 6.37) | **10.97 (3.19, 18.86)** |

Note: Bold and underlined fonts indicate statistical differences; WM, Western medicine; GZFL, Guizhi Fuling capsule; HE, Huange capsule; JGSQ, Jingui Shenqi pill; LBS, Longbishu capsule; LWDH, Liuwei Dihuang pill; PLA, Pulean tablet; QLBX, Qianlie Beixi capsule; QLS, Qianlieshu pill; QLST, Qianlie Shutong capsule; QLTY, Qianlie Tongyu capsule; QLX, Qianliexin capsule; SPE, Saw Palmetto Extract capsule; WLT, Wenglitong capsule; XLQ, Xialiqi capsule; ZGLS, Zegui Longshuang Capsule.

## Subgroup network meta-analysis for postvoid residual urine

| **Characteristics** | **GZFL+WM** | **HE+WM** | **JGSQ+WM** | **LBS+WM** | **LWDH+WM** | **LZ+WM** | **PLA+WM** | **QLBX+WM** | **QLS+WM** | **QLST+WM** | **QLTY+WM** | **QLX+WM** | **SPE+WM** | **WLT+WM** | **XLQ+WM** | **ZGLS+WM** |
| --- | --- | --- | --- | --- | --- | --- | --- | --- | --- | --- | --- | --- | --- | --- | --- | --- |
| All trials | 6.54 (-2.25, 15.27) | 4.64 (-4.13, 13.54) | **6.35 (2.61, 10.06)** | **6.6 (3.64, 9.58)** | **6.98 (1.26, 12.66)** | **5.16 (0.12, 10.13)** | **11.62 (6.78, 16.4)** | 2.62 (-2.49, 7.6) | **8.22 (1.84, 14.53)** | **9.32 (7.43, 11.18)** | 4.78 (-1.61, 11.21) | **6.32 (2.07, 10.51)** | 2.9 (-0.64, 6.51) | 6.42 (-2.55, 15.49) | **6.73 (0.42, 13.02)** | **6.28 (1.26, 11.5)** |
| Mean age |  |  |  |  |  |  |  |  |  |  |  |  |  |  |  |  |
| ＞65 years | NA | NA | **6.64 (3.94, 9.23)** | **5.67 (3.25, 8.02)** | **7.08 (3.24, 10.9)** | NA | 4.2 (-2.35, 10.84) | -0.42 (-9.2, 8.37) | **8.91 (3.28, 14.49)** | **8.69 (6.46, 10.74)** | 4.66 (-0.13, 9.47) | **6.62 (3.91, 9.2)** | 2.01 (-2.08, 6.09) | NA | **6.74 (1.97, 11.53)** | 1.14 (-4.42, 6.63) |
| ≤65 years | 6.51 (-5.09, 18.13) | 4.66 (-7.1, 16.39) | 6.01 (-5.78, 17.7) | **8.42 (2.4, 14.42)** | 6.6 (-12.09, 25.3) | 5.14 (-1.63, 11.79) | **13.73 (6.5, 20.81)** | 3.1 (-4.23, 10.24) | 7.47 (-4.28, 19.34) | **9.96 (6.88, 12.96)** | NA | NA | 3.5 (-2.03, 9.09) | 6.45 (-5.46, 18.28) | NA | **9.42 (1.69, 17.42)** |
| Trial duration |  |  |  |  |  |  |  |  |  |  |  |  |  |  |  |  |
| ≥3 months | NA | NA | **5.84 (1.87, 9.83)** | **6.61 (3.59, 9.63)** | **6.88 (1.02, 12.68)** | 4.01 (-4.35, 12.32) | NA | 2.7 (-2.28, 7.48) | **8.91 (0.32, 17.6)** | **11.05 (8.61, 13.37)** | NA | **7.06 (2.18, 12)** | 3.78 (0, 7.62) | 6.45 (-2.32, 15.25) | **6.74 (0.63, 12.86)** | 1.11 (-7.47, 9.64) |
| ＜3 months | 6.5 (-4.35, 17.15) | 4.67 (-6.12, 15.44) | 8.73 (-2.08, 19.45) | 6.52 (-3.47, 16.5) | 6.56 (-11.55, 24.82) | 5.74 (-1.8, 13.23) | **11.45 (5.65, 17.13)** | NA | 7.46 (-3.37, 18.46) | **6.88 (3.43, 10.18)** | 4.85 (-2.48, 12.28) | 4.44 (-4.33, 12.96) | -1.51 (-12.19, 9.2) | NA | NA | **9.17 (2.05, 16.65)** |
| Sample size |  |  |  |  |  |  |  |  |  |  |  |  |  |  |  |  |
| ≥100 | NA | 4.69 (-3.14, 12.44) | 4.68 (-4.57, 13.99) | **6.66 (3.81, 9.68)** | 7.56 (-1.01, 16.19) | 4.85 (-0.54, 10.21) | 4.17 (-4.49, 12.84) | 5.51 (-0.92, 11.41) | **8.94 (0.91, 16.83)** | **12.87 (10.05, 15.5)** | 4.29 (-4.78, 13.38) | **9.12 (0.95, 17.21)** | 0.35 (-4.19, 4.99) | NA | 6.8 (-2.36, 16.02) | 13.72 (-0.98, 28.5) |
| ＜100 | 6.51 (-2.79, 15.85) | NA | **6.54 (2.33, 10.8)** | 5.74 (-1.62, 12.98) | 6.75 (-0.74, 14.15) | 5.76 (-3.38, 14.88) | **14.06 (8.11, 19.79)** | -0.34 (-8.09, 7.37) | 7.5 (-1.93, 16.91) | **7.16 (4.63, 9.59)** | 5.17 (-3.45, 13.81) | **5.53 (0.46, 10.46)** | **5.18 (0.02, 10.36)** | 6.45 (-3.01, 16.01) | 6.67 (-1.61, 14.96) | 5.39 (-0.21, 11.24) |

Note: Bold and underlined fonts indicate statistical differences; WM, Western medicine; GZFL, Guizhi Fuling capsule; HE, Huange capsule; JGSQ, Jingui Shenqi pill; LBS, Longbishu capsule; LWDH, Liuwei Dihuang pill; LZ, Lingze tablet; PLA, Pulean tablet; QLBX, Qianlie Beixi capsule; QLS, Qianlieshu pill; QLST, Qianlie Shutong capsule; QLTY, Qianlie Tongyu capsule; QLX, Qianliexin capsule; SPE, Saw Palmetto Extract capsule; WLT, Wenglitong capsule; XLQ, Xialiqi capsule; ZGLS, Zegui Longshuang Capsule.
